# LINC01287 facilitates proliferation, migration, invasion and EMT of colon cancer cells via miR-4500/MAP3K13 pathway

**DOI:** 10.1186/s12885-021-08528-7

**Published:** 2021-07-06

**Authors:** Dazhi Fu, Yongjun Ren, Chunxiao Wang, Lei Yu, Rui Yu

**Affiliations:** 1 Department of General Surgery, First Affiliated Hospital of China Medical University, NO. 155 Nanjingbei Road, Heping District, Shenyang, 110001 Liaoning China; 2 Department of Interventional Radiology, Sichuan Key Laboratory of Medical Imaging, Affiliated Hospital of North Sichuan Medical College, Nanchong, 637000 Sichuan China; 3 Department of General Surgery, Liaoning Health Industry Group, Benxi Iron & Steel Industry Group General Hospital, Benxi, 117000 Liaoning China

**Keywords:** LINC01287, miR-4500, MAP3K13, Colon cancer

## Abstract

**Background:**

Accumulated studies indicate that aberrant expression of long noncoding RNAs (lncRNAs) is associated with tumorigenesis and progression of colon cancer. In the present study, long intergenic non-protein coding RNA 1287 (LINC01287) was identified to up-regulate in colon cancer by transcriptome RNA-sequencing, but the exact function remained unclear.

**Methods:**

Transcriptome RNA-sequencing was conducted to identify dysregulated lncRNAs. Expression of LINC01287 was evaluated by real-time quantitative PCR. The downstream targets of LINC01287 and miR-4500 were verified by luciferase reporter assay, pull down assay and western blot. The potential functions of LINC01287 were evaluated by cell viability assay, colony formation assay, soft agar assay, flow cytometry, transwell migration and invasion assay, and tumor xenograft growth in colon cancer cells.

**Results:**

Our results indicated that LINC01287 was up-regulated in colon cancer patients. High LINC01287 expression was associated with advanced TNM stage, lymph node metastasis, distant metastasis and shorter overall survival. Knockdown of LINC01287 inhibited cell growth, colony formation in plates and soft agar, transwell cell migration and invasion, and epithelial-mesenchymal transition (EMT) of colon cancer cells, while LINC01287 overexpression had contrary effects. In addition, LINC01287 mediated MAP3K13 expression by sponging miR-4500, thus promoted NF-κB p65 phosphorylation. Restored MAP3K13 expression or miR-4500 knockdown partially abrogated the effects of silencing LINC01287 in colon cancer cells.

**Conclusion:**

Our findings demonstrated that the LINC01287/miR-4500/MAP3K13 axis promoted progression of colon cancer. Therefore, LINC01287 might be a potential therapeutic target and prognostic marker for colon cancer patients.

**Supplementary Information:**

The online version contains supplementary material available at 10.1186/s12885-021-08528-7.

## Background

Colon cancer is a highly aggressive malignancy. There are 1.8 million new colon cancer cases and 900,000 colon cancer related deaths all over the world each year [1]. Dietary habits, age, obesity, smoking and lack of physical exercise are risk factors for colon cancer [2]. Noteworthily, colon cancer cases are rapidly increased in Asian-Pacific region, mainly ascribed to the growing industrialization and urbanization [3]. Although current therapeutic strategies such as chemotherapy, targeted therapy, and immunotherapy achieve marked improvements in the past decades, the prognosis for colon cancer patients remains poor due to late diagnosis and lack of screening methods [4, 5]. Moreover, the molecular pathogenesis of colon cancer is not fully understood, which limits the strategy for improving the survival of colon cancer patients. Thus, efforts in resolving these issues would obtain a better understanding of this lethal disease and facilitate development of new effective prognostic biomarkers or therapeutic targets for colon cancer.

Long non-coding RNAs (lncRNAs) are transcripts longer than 200 nucleotides in length but with little or no protein-coding potential [6]. LncRNAs are involved in many cellular processes, such as proliferation, apoptosis, differentiation, stem cell pluripotency and epithelial-mesenchymal transition (EMT) [7, 8]. Notably, recent studies show that many lncRNAs play vital roles in cancer development and progression. For example, lincRNA-p21, BCAR4, MEG3 GLCC1 and H19 are involved in the tumorigenesis of many cancers [9]. Besides, there are growing number of studies indicating that lncRNAs also act a vital role in the tumorigenesis and chemoresistance of colon cancer. Transcriptome RNA-sequencing of colon cancer tissues in our study identified long intergenic non-protein coding RNA 1287 (LINC01287) as one of the top 50 dysregulated lncRNAs. LINC01287, which locates on 7q36.2 of human genome, is a newly discovered and highly conserved lncRNA among mammalian tissues. Recent studies indicate that LINC01287 is involved in the carcinogenesis of hepatocellular carcinoma and breast cancer [10–12]. Moreover, other studies also suggest that dysregulated LINC01287 has prognostic value in gastric cancer and lung cancer [13, 14]. However, the biological function of LINC01287 in colon cancer remains unclear.

MAP3K13, also known as Leucine Zipper-bearing Kinase (LZK), belongs to the mitogen-activated protein kinase kinase kinase (MAPKKK) family. It has high sequence identity to Dual Leucine Zipper Kinase (DLK/MAP3K12). Previous studies indicate that MAP3K13 is a critical regulator of astrocyte reactivity and promotes axon growth in mammalian central nervous system [15, 16]. Moreover, MAP3K13 is reported to regulate JNK and NF-κB pathways, which can be pro-tumorigenic [17, 18]. Indeed, MAP3K13 exerts oncogenic activity in various cancers. In head and neck cancer, MAP3K13 is amplified and silencing MAP3K13 causes reducing in colony formation and tumor growth and destabilizing of mutant p53 [19]. In breast cancer, high MAP3K13 expression is associated with poor patient survival. Enforced MAP3K13 expression in breast cancer cells stabilizes MYC oncogene and promotes its transcriptional activity [20].

In our study, LINC01287 was identified as one of the top 50 up-regulated lncRNAs in colon cancer tissues by transcriptome RNA-sequencing of four paired colon cancer samples. Silencing LINC01287 suppressed cell growth, colony formation in plates and soft agar, transwell migration and invasion, and EMT of colon cancer cells, while enforced LINC01287 expression had converse effects. In addition, we found that LINC01287 mediated MAP3K13 expression by sponging miR-4500. Our data suggested that LINC01287 might be a potential biomarker and promising therapeutic target for colon cancer.

## Methods

### Patient samples

Sixty-four colon cancer patients were enrolled in our study from May 2014 to October 2015. All these patients knew the study concept, agreed to participate and signed the informed consents. This study was reviewed and approved by the Ethics Committee of First Affiliated Hospital of China Medical University. Sixty-four colon cancer samples and paired adjacent normal tissues were collected from the First Affiliated Hospital of China Medical University. Tissue samples were snap frozen and stored at − 80 °C. Patient characteristics were obtained from the hospital database. To evaluate patient survival, the patients were followed up for as long as 48 months post-surgery.

### Transcriptome RNA-sequencing

Total RNAs of colon cancer samples and paired normal tissues were extracted using TRIzol reagent (Invitrogen, Carlsbad, California, USA) as the protocol indicated. The complementary DNA library was prepared and sequenced at Beijing Novel Bioinformatics Co., Ltd. (https://en.novogene.com/) according to the Illumina standard protocol. Fastp and Fastqc (v0.11.5) (http://www.bioinformatics.babraham.ac.uk/projects/fastqc/) were employed to conduct adapter trimming and quality filtering. Gene expression quantification was evaluated by HTSeq V0.6.1. Dysregulated lncRNAs and mRNAs were analyzed by the DESeq R package (1.10.1). The differentially expressed lncRNAs and mRNAs were defined as adjusted *p* value < 0.05 and |log_2_ Fold Change| ≥ 2.

### Cell culture

The colon cancer cell lines (HCT116, COLO320, T24, HT29, SW480, SW948 and SW1417) used in our study were purchased from American Type Culture Collection (ATCC). The colon cancer cell line COLO678 was obtained from Deutsche Sammlung von Mikroorganismen und Zellkulturen (DSMZ). Human HEK293T was purchased from ATCC. The culture medium for SW480 and HEK293T was RPMI-1640 medium (Invitrogen, Carlsbad, California, USA) supplemented with 10% fetal bovine serum (Hyclone, Logan, Utah, USA), 100 U/mL Penicillium and 100 μg/mL Streptomycin. The culture medium for HCT116, COLO320, T24, HT29, SW480, SW948 and SW1417 were DMEM: F12 (1:1) medium (Thermo Fisher Scientific, Waltham, Massachusetts, USA) supplemented with 10% fetal bovine serum (Hyclone, Logan, Utah, USA), 100 U/mL Penicillium and 100 μg/mL Streptomycin. Cells were maintained at 37 °C in a humidified atmosphere with 5% CO_2_. Replaced the culture medium 2–3 times every week.

### Plasmid constructs, lentivirus package and transfection

Lentiviral vector for LINC01287 or MAP3K13 was built by cloning the coding sequences for LINC01287 or MAP3K13 into the pCDH lentiviral vector (System Biosciences #CD510B-1, Palo Alto, California, USA). Empty vector (EV) control was the unmodified pCDH lentiviral vector. To knock down LINC01287 in colon cancer cells, two Short hairpin RNAs (shRNAs) specifically targeting LINC01287 (sh-LINC01287–1 and sh-LINC01287–2) were designed and inserted into the pLKO.1 plasmid. The control was unmodified pLKO.1 plasmid (sh-ctrl). To overexpress miR-4500 in colon cancer cells, the mature sequences for miR-4500 were cloned into the pCMV-MIR lentiviral vector (OriGene #PCMVMIR, Rockville, Maryland, USA). The miR-ctrl was unmodified pCMV-MIR lentiviral vector. To knock down miR-4500 in colon cancer cells, the anti-sense sequences of miR-4500 were cloned into the miRZip™ lentivector-based vector (System biosciences# MZIP1-PA-1, Palo Alto, California, USA) to construct the anti-miR-4500 expression plasmid. The anti-miR-ctrl was unmodified miRZip™ lentivector-based vector. Recombinant lentiviral particles were produced in HEK293T cells by co-transfecting with lentiviral vector and helper plasmids pCMV-VSV-G, pRSV-REV, and pMDL using lipofectamine 3000 (Invitrogen, Carlsbad, California, USA). Collected the virus-containing medium at 24, 48 and 72 h post-transfection. The virus-containing medium was filtered by 0.22 μm filter and stored at − 80 °C. Lipofectamine 3000 (Invitrogen, Carlsbad, California, USA) was used for transient transfection according to the manufacture’s instruction. LINC01287 specific shRNA sequences were designed as: sh-LINC01287–1, 5′-CTGAT CAACT GAGAT GCAAA ACC-3′; sh-LINC01287–2, 5′-AAGGT TGATA CATAC GATAT TAA-3′.

### Cell viability assay

The viability of colon cancer cells was measured by the CellTiter-Glo Luminescent Cell Viability Assay kit (Promega #G7572, Madison, Wisconsin, USA) according to the manufacturer’s instruction. In brief, colon cancer cells were seeded in 96-well plates at a density of 2500 cells/well. At the indicated time points, the cells and CellTiter-Glo reagents were equilibrated to room temperature for 30 min. Then removed the culture medium and added 100 μL CellTiter-Glo reagents into each well. Mixed thoroughly on an orbital shaker and incubated for 10 min avoiding light to stable the luminescence signal. The strength of the luminescence signal was measured by microplate reader. Each sample had three repeats.

### Colony formation assay

Colon cancer cells were dispersed as single cells, then seeded in 6-well plates (1500 cells/well). Colonies were allow to grow for 2 weeks without disturbance. Then, removed the culture medium and washed cells with PBS for twice. Next, fixed the cells with 4% paraformaldehyde for 15 min at room temperature. Washed the cells with PBS for twice, then stained the cells with crystal violet solution (0.5%) for 30 to 60 min at room temperature. Then, washed cells with flow water and dried at room temperature. The colonies were scanned by a high-resolution scanner. Relative cell confluence was calculated by Image J. Each sample was done in triplicates.

### Soft agar assay

Soft agar colony formation assay was performed according to previous report [21]. Briefly, colon cancer cells were digested as single cells, then seeded in 0.35% top agar at 10000 cells/well in 6-well plates. The bottom agar is 0.6%. Cells were allowed to grow in soft agar for 2 weeks until colonies were visible under microscope. Then, images were taken using the LSM 5 Pa Laser Scanning Microscope (Zeiss Germany, Oberkochen, Baden-Wurttemberg, Germany). All samples had three repeats.

### Flow cytometry

Cell cycle of colon cancer cells was evaluated by flow cytometry. Briefly, cells were dispersed as single cell suspension by 0.05% trypsin. 1 × 10^6^ cells were collected and fixed by ice-cold 70% ethanol overnight. Next, cells were washed with ice-cold PBS for twice, then incubated with 10 μL propidium iodide (PI) at 4 °C for 15 min in a dark room. The residual RNA was digested with 100 μg/ml RNaseA for 30 min at room temperature. The cell cycle of colon cancer cells was then evaluated by Gallios Flow Cytometer (Beckman Coulter).

### Transwell cell migration and invasion assay

To evaluate cell migration, colon cancer cells were dispersed as single cells by 0.05% trypsin. Then, a total of 1× 10^6^ cells were suspended in 0.5 mL culture medium without serum and added into the transwell upper chamber (Costar Corp, Stamford, Connecticut, USA). Filled the lower chamber with culture medium containing 20% FBS. Next, cells were cultured for 48 h without disturbance. The cells migrated towards the lower chamber were fixed by 4% paraformaldehyde for 15 min at room temperature. Washed the cells twice with PBS, then incubated the cells with 0.5% crystal violet for 30 min at room temperature. Washed away the residual crystal violet solution with PBS for several times, then cells were photographed by a microscope. To evaluate cell invasion, the filter of the upper chamber was pre-coated with Matrigel (BD Biosciences, San Jose, California, USA). The other procedures for transwell invasion assay were same as these in transwell migration assay. Each sample in the transwell migration or invasion assay had three repeats.

### Western blot

Protein lysates were extracted from culture cells or tissue samples using RIPA buffer (Beyotime, Beijing, China) supplemented with protease inhibitors (Sigma-Aldrich, St. Louis, Missouri, USA). The protein concentration in cell lysates was quantified by BCA kit (Thermo Fisher Scientific, Waltham, Massachusetts, USA) according to the manufacturer’s instruction. Next, proteins (30 μg) were separated by 10% or 15% SDS-PAGE, and transferred onto nitrocellulose membranes. The membranes were blocked with 5% non-fat milk for 1 h at room temperature, then incubated with specific first antibodies at 4 °C overnight. The membranes were washed with ice-cold Tris buffered saline Tween (TBST) for five times, then incubated with corresponding second antibodies for 1 h at room temperature. The specific antibodies were listed below: MAP3K13 (Abcam # ab230467, 1: 1000, Cambridge, Massachusetts, USA), E-cadherin (Santa Cruz, Cat# sc-8426, 1: 1000, Dallas, Texas, USA), N-cadherin (Santa Cruz, Cat# sc-393,933, 1: 1000, Dallas, Texas, USA), Vimentin (Santa Cruz, Cat# sc-6260, 1: 1000, Dallas, Texas, USA), NF-κB p65 (Cell signaling, Cat# 8242, 1: 1000, Danvers, Massachusetts, USA), Phospho-NF-κB p65 (Ser536) (Cell signaling, Cat# 3033, 1: 1000, Danvers, Massachusetts, USA), JNK Antibody (Cell signaling, Cat# 9252, 1: 1000, Danvers, Massachusetts, USA), Phospho-SAPK/JNK (Thr183/Tyr185) Antibody (Cell signaling, Cat# 9251, 1: 1000, Danvers, Massachusetts, USA), GAPDH (Cell signaling, Cat# 5174, 1: 1000, Danvers, Massachusetts, USA). The second antibodies were goat anti-rabbit IgG HRPlinked antibody (Cell signaling #7074; 1: 4000, Danvers, Massachusetts, USA) and sheep anti-mouse IgG-HRP (GE/Amershan #NXA931; 1: 5000, Marlborough, Massachusetts, USA). Enhanced chemiluminescence kit (Amersham, USA) was used to determine the protein bands on the Amersham Imager 600 imagers (GE Healthcare, USA).

### Real-time quantitative PCR

Total RNAs were isolated by TRIzol Reagent (Thermo Fisher Scientific, Waltham, Massachusetts, USA) as protocol indicated. NanoDrop 2000 was used to quantify RNA concentration. Complementary DNAs were synthesized by cDNA Reverse Transcription kit (Takara Biotechnology, Kusatsu, Shiga, Japan). Real-time quantitative PCR was performed on the Applied Biosystems StepOne Plus Real-Time PCR Systems using the SYBR Green reagent (Thermo Fisher Scientific, Waltham, Massachusetts, USA). GAPDH or U6 was used as internal control. The PCR primers were listed as follows: LINCO1287 forwards: 5′-GGTTG ATGTA AGGAC CTCGT-3′, and reverse: 5′-GAGAC CTTGT TTCAT GTGTCG-3′. MiR-4500 forwards: 5′-TGAGG TAGTA GTTTC TTGCG CC-3′, and reverse: 5′-CTCTA CAGCT ATATTG CCAGC CAC-3′. MAP3K13 forwards: 5′-AGCAG CAGTT GGTAG TGAGG TT-3′, and reverse: 5′-GGAAG TGGTC AGCAG GCAGAA-3′. GAPDH forwards: 5′-TGACA ACTTT GGTAT CGTGG AAGG-3′, and reverse: 5′-AGGCAG GGATG ATGTT CTGGA GAG-3′. U6 forwards: 5′- ATACAG AGAAA GTTAG CACGG-3′, and reverse: 5′- GGAAT GCTTC AAAGA GTTGTG-3′. Oct4 forwards: 5′- CGCCG TATGA GTTCT GTG − 3′, and reverse: 5′- GGTGA TCCTC TTCTG CTTC − 3′. Lin28 forwards: 5′- AAAGG AGACA GGTGC TAC − 3′, and reverse: 5′- ATATG GCTGA TGCTC TGG − 3′. Nanog forwards: 5′- AAGAA CTCTC CAACA TCCTG AAC − 3′, and reverse: 5′- CCTTC TGCGT CACAC CATT − 3′. Sox2 forwards: 5′- AGTTG GACAG GGAG ATGGC − 3′, and reverse: 5′- AACCT TCCTT GCTTC CACG − 3′. Relative gene expression was calculated by the 2^−ΔΔCq^ method. Each sample had three repeats.

### Luciferase reporter assay

The 3′-UTR of LINC01287 or MAP3K13 containing the putative binding sites for miR-4500 was amplified and cloned into the pMIRREPORT vector (Promega, Madison, Wisconsin, USA). GeneTailor™ Site-Directed Mutagenesis System (Invitrogen, Carlsbad, California, USA) was used to establish 3-bp mutations at the putative miR-4500 binding sites for LINC01287 or MAP3K13. The pMIRREPORT plasmid containing the 3′ UTR of LINC01287 or MAP3K13, the miR-4500 expression plasmid or miR-ctrl, and a renilla luciferase plasmid (2: 2: 1) were transient transfected into HEK293T cells by lipofectamine 3000 (Invitrogen, Carlsbad, California, USA). Luciferase activity was quantified by the Dual Luciferase Reporter Assay System ((Promega, Madison, Wisconsin, USA) at 48 h post-transfection. Each sample in luciferase reporter assay had three repeats at the same time.

### Tumor xenograft model

Animal studies were reviewed and approved by the Animal Care and Experimental Committee of First Affiliated Hospital of China Medical University, and performed in accordance with relevant protocols. Before cell injection, T84 cells (2 × 10^6^) were infected with LINC01287 expression lentivirus or EV control. Next, these cells were subcutaneously injected into 6-week old BALB/c nude mice (Jackson Laboratories, Shanghai, China). The length and width of tumor xenograft were measured by caliper every 3 days. The formula (length × width^2^)/2 was used to calculate tumor volume. All mice were anaesthetized by inhalation of 3% isoflourane and sacrificed by breaking the neck at 5 weeks after tumor implantation. Tumor xenografts were fetched out, photographed and weighed.

### Statistical analysis

GraphPad Prism 8.0 was used to draw graphs and analyze the data in our study. Overall survival of colon cancer patients were evaluated by the Kaplan–Meier method (Logrank test) of the GraphPad Prism 8.0 software. The data was depicted as mean ± standard deviation (SD). Difference of two groups was evaluated by the two-tailed Student’s t-test. Difference of multiple groups was evaluated by One-way ANOVA (LSD post-hoc test). *p* < 0.05 indicated a statically significant difference.

## Results

### LINC01287 is up-regulated in colon cancer tissues and closely associated with poor prognosis

**Fig. 1:**
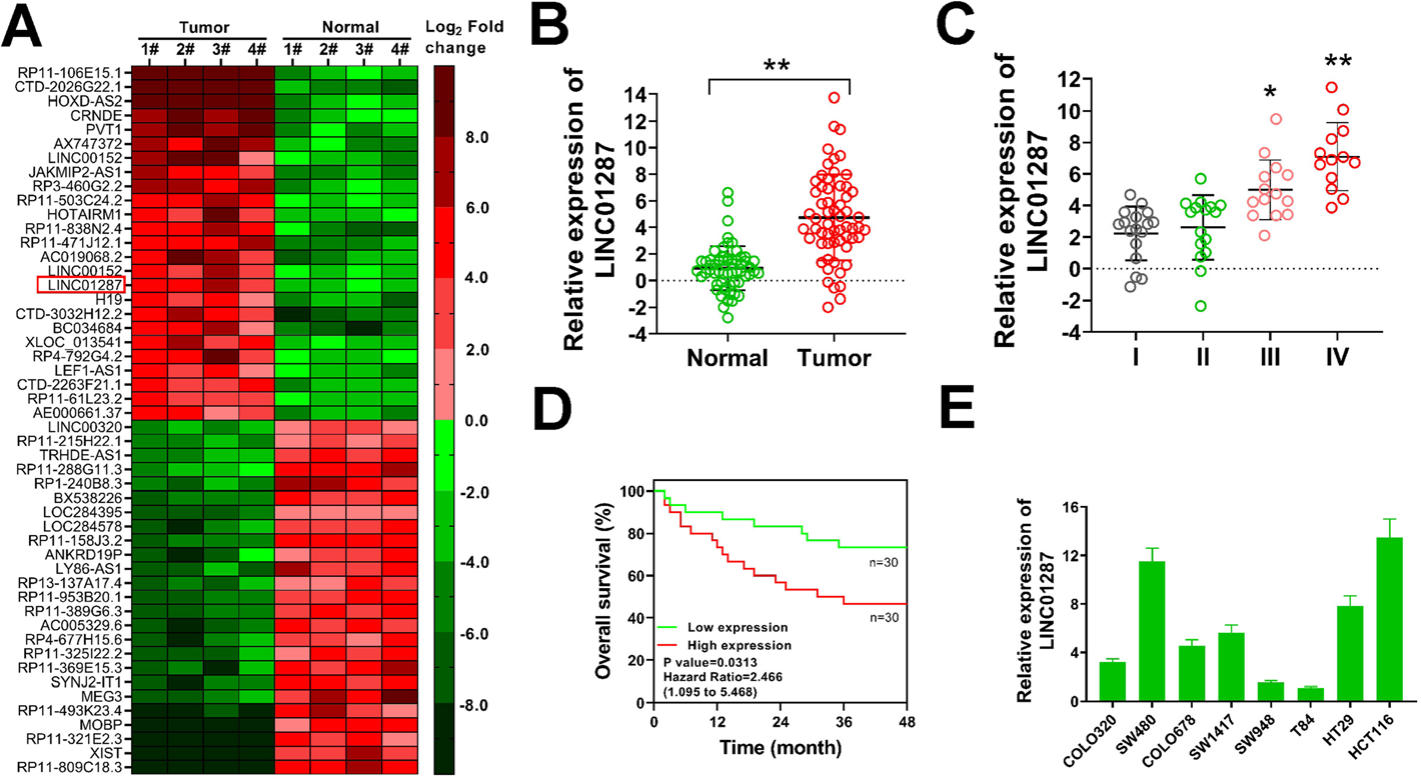
LINC01287 is up-regulated in colon cancer tissues and closely associated with poor prognosis. **A**, heat map showed the top 50 dyregulated lncRNAs in 4 colon cancer tissues and paired normal samples identified by transcriptome sequencing. **B**, relative LINC01287 expression in 60 colon cancer tissues and paired normal samples was tested by qRT-PCR. Compared with paired normal samples using two-tailed Student’s t-test, **p ≤ 0.001. **C**, relative level of LINC01287 in colon cancer patients according to tumor stage. Compared with tumor stage I using two-tailed Student’s t-test, *p ≤ 0.05, **p ≤ 0.001. **D**, overall survival of colon cancer patients with high or low LINC01287 expression was plotted by Kaplan-Meier survival method. **E**, expression of LINC01287 in colon cancer cells was tested by qRT-PCR

In order to explore novel genes associated with colon cancer progression, we performed transcriptome RNA-sequencing analysis of 4 colon cancer tissues and paired adjacent normal samples. The genes with a *p* value < 0.05 and |log_2_ Fold Change| ≥ 2 were considered as differentially expressed. In our study, 929 lncRNAs were identified to differentially express in colon cancer tissues compared with normal tissues, including 328 significantly up-regulated lncRNAs and 601 down-regulated lncRNAs (Supplementary Table 1). The top 25 up-regulated and down-regulated lncRNAs were depicted in Fig. 1A. Among them, dysregulation of CRNDE, PVT1, LINC00152, H19, LEF1-AS1, MEG3 and XIST were proved to involve in the initiation, progression, metastasis and chemoresistance of colon cancer cells, indicating that our transcriptome RNA-sequencing was successful and feasible to explore lncRNAs playing a role in colon cancer tumorigenesis [22–25]. Using the same cut-off criteria as lncRNAs, a total of 1069 different expressed mRNAs (DEmRNAs) were identified, including 662 up-regulated and 407 down-regulated DEmRNAs (Supplementary Table 2).

We focused on LINC01287 as it ranked the top 25 up-regulated lncRNAs in our study, and previous studies report that LINC01287 acts a role in the tumorigenesis of hepatocellular carcinoma and breast cancer [10–12]. However, whether LINC01287 was oncogenic in colon cancer was unknown. To validate the RNA-seq results, expression of LINC01287 was analyzed in another enlarged cohort of 60 colon patients by qRT-PCR. Our results proved that LINC01287 was apparently up-regulated in colon cancer tissues (Fig. 1B). Then, we performed a correlation analysis between LINC01287 and clinicopathological features of colon cancer patients. The median expression of LINC01287 was used to divide colon cancer patients into high or low LINC01287 expression group. In the present study, high expression of LINC01287 was found to correlate with advanced TNM stage of colon cancer patients (Fig. 1C and Table 1). Moreover, up-regulated LINC01287 was closely associated with lymph node metastasis and distant metastasis, too (Table 1). High expression of LINC01287 also predicted a shorter overall survival (Fig. 1D). The expression of LINC01287 in colon cancer cell lines was examined by qRT-PCR. Our data indicated that SW480, HT29 and HCT116 cells had high endogenous LINC01287 levels while SW948 and T84 cells had low endogenous LINC01287 levels (Fig. 1E). Taken together, our results indicated that LINC01287 was obviously up-regulated and connected with advanced TNM stage, lymph node metastasis and distant metastasis of colon cancer patients, suggesting that it might contribute to the carcinogenesis of colon cancer.

**Table 1:** Correlation between LINC01287 expression and clinicopathological characteristics in colon cancer patients

Characteristics	n	LINC01287 expression		*P* value
		Low	High	
Age (Years)				0.851
≤ 60	34	20	14	
>60	26	10	16	
Sex				0.575
Male	27	12	15	
Female	33	18	15	
Tumor size (mm)				0.193
≥ 30	41	17	24	
<30	19	13	6	
TNM stage				**0.001**
I-II	33	24	9	
III-IV	27	6	21	
Histological grade				0.158
Well	29	17	12	
Moderate-Poor	31	13	18	
Lymph node metastasis				**0.004**
Positive	43	16	27	
Negative	17	14	3	
Distant metastasis				**0.001**
Positive	29	9	20	
Negative	31	21	10	

### Silencing LINC01287 suppresses proliferation, migration, invasion and EMT of colon cancer cells

**Fig. 2:**
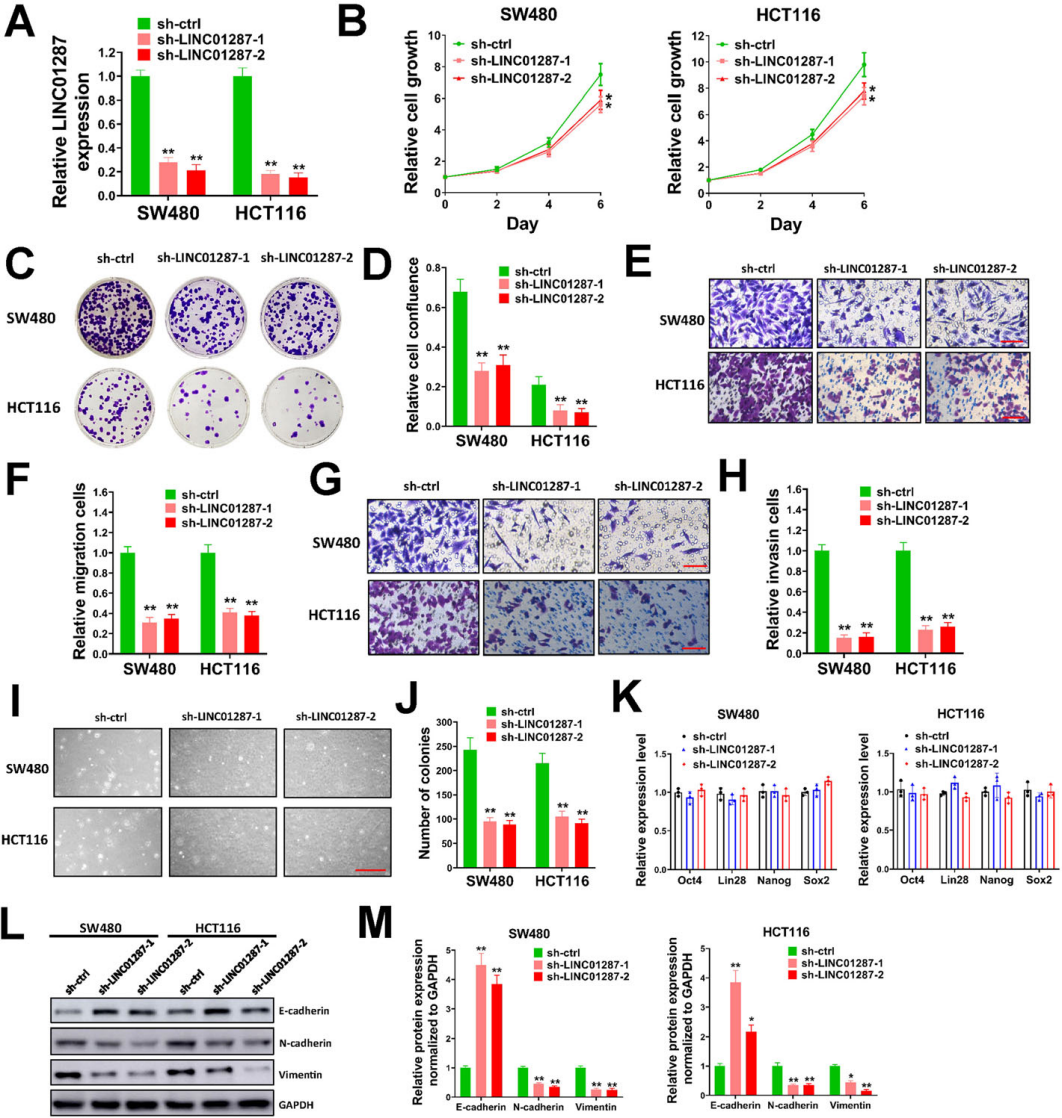
Silencing LINC01287 suppressed proliferation, migration, invasion and EMT of colon cancer cells. **A**, LINC01287 expression in SW480 and HCT116 cells infected with sh-LINC01287–1, sh-LINC01287–2 or sh-ctrl lentivirus was evaluated by qRT-PCR. **B**, SW480 and HCT116 cells infected with sh-LINC01287–1, sh-LINC01287–2 or sh-ctrl lentiviral particles were seeded in 96-well plates (2500/well), then cell viability was evaluated at day 0, 2, 4 and 6 post-infection. **C**-**D**, SW480 and HCT116 cells infected with sh-LINC01287–1, sh-LINC01287–2 or sh-ctrl lentiviral particles were seeded in 6-well plates (1500/well) and grew for 2 weeks for colony formation assay. Representative images for colonies (**C**) and relative cell confluence of all colonies were shown (**D**). E-H, SW480 and HCT116 cells infected with sh-LINC01287–1, sh-LINC01287–2 or sh-ctrl lentiviral particles were used for transwell cell migration (**E**-**F**) or invasion (**G**-**H**) assay. Representative images for migration cells (**E**) or invasion cells (**G**), and relative migration (**F**) and invasion (**H**) cells were shown. Scale bar = 50 μm. I-J, SW480 and HCT116 cells infected with sh-LINC01287–1, sh-LINC01287–2 or sh-ctrl lentiviral particles were used for soft agar assay. Representative images (**I**) and colony numbers per well (**J**) were shown. Scale bar = 500 μm. K, SW480 and HCT116 cells were infected with sh-LINC01287–1, sh-LINC01287–2 or sh-ctrl lentiviral particles, then expression levels of indicated genes were measured by qRT-PCR. L-M. SW480 and HCT116 cells were infected with sh-LINC01287–1, sh-LINC01287–2 or sh-ctrl lentiviral particles, then protein expression levels of indicated genes were tested by western blot (**L**). Uncropped gels were in Supplementary Fig. 1. Relative protein expression compared with GAPDH was shown (**M**). Compared with sh-ctrl group using two-tailed Student’s t-test, *p ≤ 0.05, **p ≤ 0.001

To explore the potential function of LINC01287 in colon cancer, we constructed two LINC01287 shRNA lentivirus (sh-LINC01287–1 and sh-LINC01287–2). Then, LINC01287 was knocked down in SW480 and HCT-116 cells which had high endogenous levels of LINC01287. The knockdown efficiency was confirmed by qRT-PCR. We demonstrated that LINC01287 was depleted by sh-LINC01287–1 or sh-LINC01287–2 in SW480 and HCT-116 cells (Fig. 2A). The effect of LINC01287 knockdown on cell proliferation was evaluated. Our results indicated that silencing LINC01287 caused a decrease in cell growth of SW480 and HCT116 cells compared with the sh-ctrl group (Fig. 2B). In colony formation assay, silencing LINC01287 in SW480 and HCT116 cells suppressed colony formation (Fig. 2C and D). In transwell cell migration and invasion assays, we found that silencing LINC01287 caused an obviously decreased in migration and invasion cells of SW480 and HCT116 compared with sh-ctrl group (Fig. 2E-H). The influence of LINC01287 on spheroid formation of colon cancer cells was evaluated by soft agar assay. We found that knockdown of LINC01287 obviously reduced the number of colonies formed by SW480 and HCT116 cells in soft agar, indicating that knockdown of LINC01287 suppressed anchorage-independent growth (Fig. 2I and J). We also tested the impact of LINC01287 on stemness of colon cancer cells, but unfortunately LINC01287 knockdown showed no influence on the expression levels of stemness markers such as Oct4, Lin28, Nanog and Sox2 (Fig. 2K). The levels of EMT makers (E-cadherin, N-cadherin and Vimentin) were examined by western blot. The expression of E-cadherin was increased while N-cadherin and Vimentin levels decreased after LINC01287 knockdown, indicating that silencing LINC01287 inhibited EMT process (Fig. 2L and M). Collectively, our data demonstrated that LINC01287 knockdown restrained proliferation, migration, invasion and EMT of colon cancer cells.

### Enforced LINC01287 expression promotes proliferation, migration, invasion, EMT and xenograft formation of colon cancer cells

**Fig. 3:**
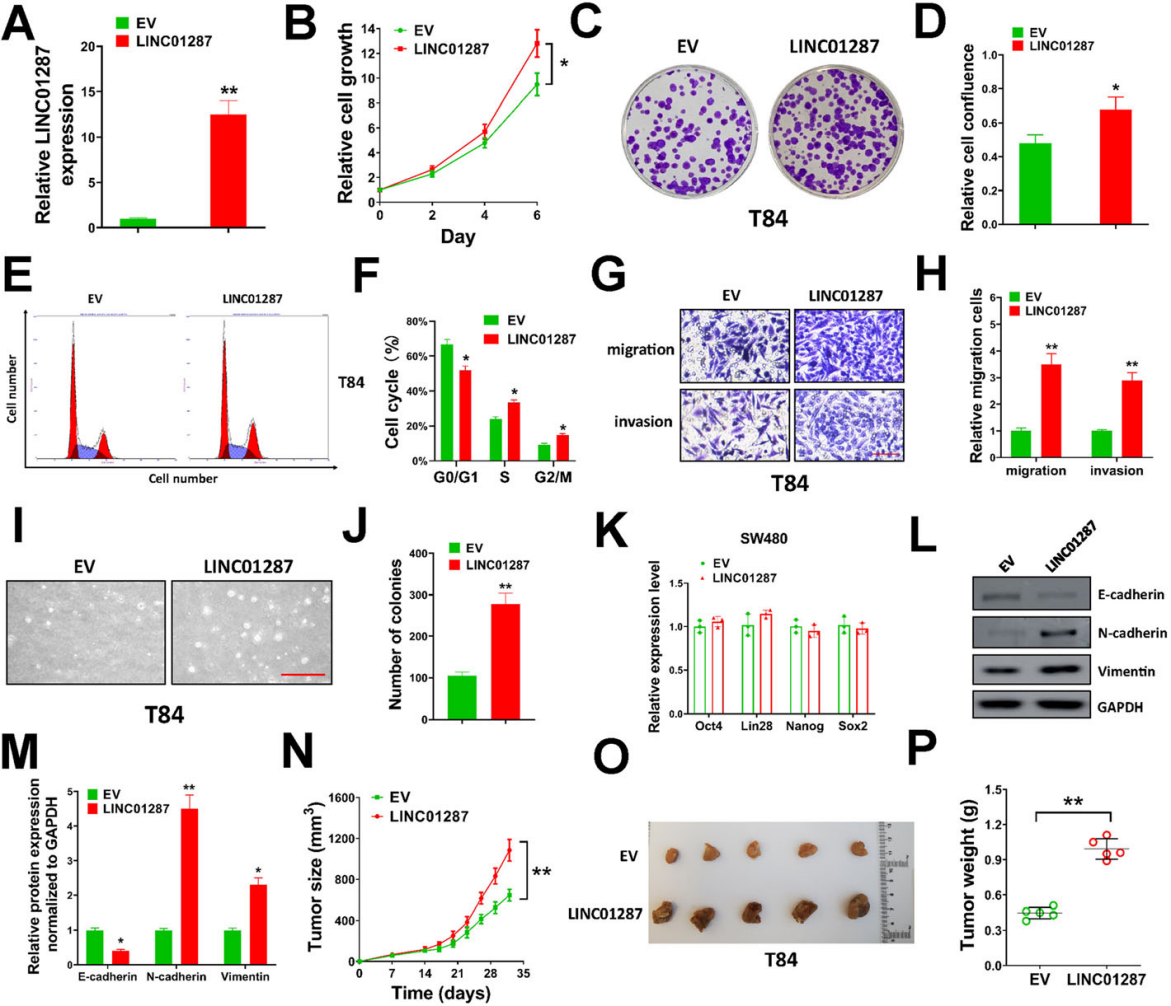
Enforced LINC01287 expression promotes proliferation, migration, invasion, EMT and xenograft formation of colon cancer cells. **A**, T84 cells were infected with LINC01287 or empty vector (EV) lentiviral particles, then LINC01287 levels were detected by qRT-PCR. **B**, T84 cells infected with LINC01287 or empty vector (EV) lentiviral particles were seeded in 96-well plates (2500/well), then cell viability was measured at indicated time points. **C**-**D**, T84 cells infected with LINC01287 or EV lentiviral particles were seeded in 6-well plates (1500/well) and grew for 2 weeks for colony formation assay. Representative images of colonies (**C**) and relative cell confluence of all colonies (**D**) were exhibited. E-F, T84 cells infected with LINC01287 or EV lentiviral particles were used for cell cycle analysis by flow cytometry. Representative images (**E**) and percentage of cells in each cell cycle stage were shown (**F**). **G**-**H**, T84 cells infected with LINC01287 or EV lentiviral particles were used for transwell cell migration or invasion assay. Representative images for migration cells or invasion cells (**G**), and relative migration and invasion (**H**) cells were shown. Scale bar = 50 μm. I-J, T84 cells infected with LINC01287 or EV lentiviral particles were used for soft agar assay. Representative images (**I**) and number of colonies per well (**J**) were shown. Scale bar = 500 μm. K, T84 cells were infected with LINC01287 or EV lentiviral particles, then expression levels of indicated genes were tested by qRT-PCR. L-M. T84 cells were infected with LINC01287 or EV lentiviral particles, then protein expression levels of indicated genes were tested by western blot (**L**). Uncropped gels were in Supplementary Fig. 1. Relative protein expression compared with GAPDH was shown (**M**). N-P, T84 cells (2 × 10^6^) infected with LINC01287 or EV lentiviral particles were subcutaneously injected into nude mice, then tumor xenografts were allowed to grow for 5 weeks. Tumor growth (**N**), images for tumor xenografts (**O**) and weight of tumors (**P**) were shown. Compared with EV group using two-tailed Student’s t-test, *p ≤ 0.05, **p ≤ 0.001

To further validate the effect of LINC01287, we enforced LINC01287 expression in T84 cells which had low endogenous LINC01287 expression level. The expression of LINC01287 was verified by qRT-PCR, and our results indicated that we successfully overexpressed LINC01287 in T84 cells (Fig. 3A). Furthermore, enforced LINC01287 expression facilitated cell proliferation of T84 cells (Fig. 3B). In colony formation assay, T84 cells transduced with LINC01287 expression lentivirus formed more colonies than EV group (Fig. 3C and D). In flow cytometry, T84 cells transduced with LINC01287 expression vector showed increase percentages of cells in S and G2 phase, indicating increased cell proliferation (Fig. 3E and F). In transwell migration and invasion assays, enforced LINC01287 expression in T84 cells significantly increased the number of migration and invasion cells (Fig. 3G and H). In soft agar assay, LINC01287 overepxression promoted anchorage-independent growth of T84 cells (Fig. 3I and J). Besides, we found that LINC01287 overexpression had no influence on the expression of Oct4, Lin28, Nanog and Sox2 (Fig. 3K). Furthermore, LINC01287 overexpression suppressed E-cadherin expression, and up-regulated N-cadherin and Vimentin levels in T84 cells (Fig. 3L and M). For in vivo studies, we generated a tumor xenograft model by subcutaneously injecting T84 cells into nude mice. We found that LINC01287 overexpression markedly increased the tumor growth rate, tumor volume and weight compared with EV group (Fig. 3N-P). Above all, our results confirmed that up-regulated LINC01287 promoted proliferation, migration, invasion, EMT and tumor xenograft formation of colon cancer cells in vitro and in vivo.

### LINC01287 serves as a molecular sponge for miR-4500 in colon cancer

**Fig. 4:**
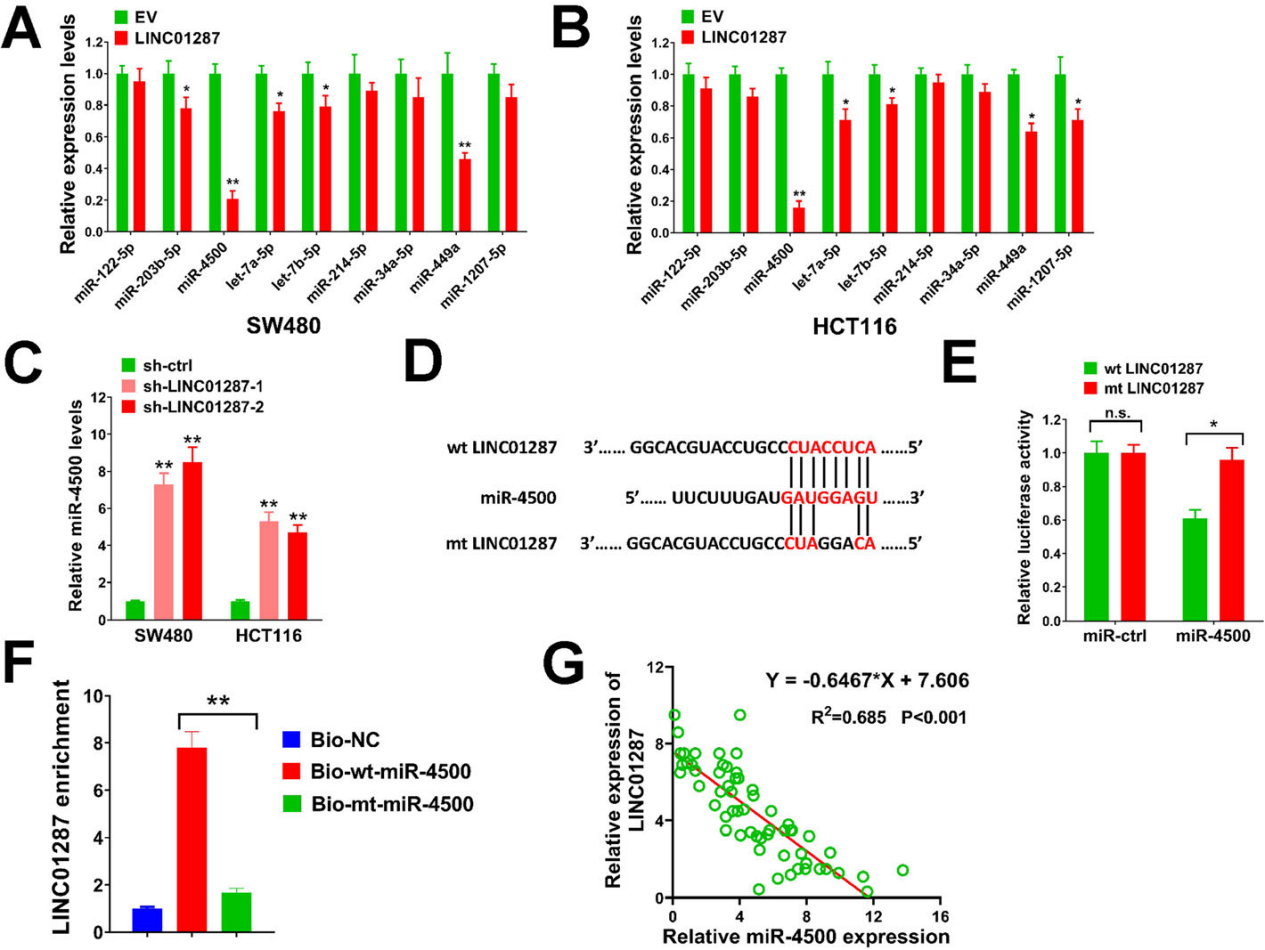
LINC01287 serves as a molecular sponge for miR-4500 in colon cancer. **A**-**B**, SW480 and HCT116 cells were infected with LINC01287 or EV lentiviral particles, then expression levels of indicated microRNAs were tested by qRT-PCR. **C**, SW480 and HCT116 cells were infected with sh-LINC01287–1, sh-LINC01287–2 or sh-ctrl lentiviral particles, then miR-4500 level was measured by qRT-PCR. **D**, the predicted binding sites of LINC01287 and miR-4500. **E**, The pMIRREPORT plasmid containing the wt or mt 3′ UTR of LINC01287, the miR-4500 expression plasmid or miR-ctrl, and a renilla luciferase plasmid (2: 2: 1) were transient transfected into HEK293T cells for luciferase reporter assay. **F**, the levels of LINC01287 pulled down by biotinylated wt or mt miR-4500, or non-targeting control (NC) were tested by qRT-PCR. **G**, spearman analysis of correlation between LINC01287 and miR-4500 expression in 60 colon cancer patients. Compared with EV or Bio-NC group using two-tailed Student’s t-test, *p ≤ 0.05, **p ≤ 0.001. n.s. = not significant

Accumulated studies prove that lncRNAs can regulate downstream gene expression via the competing endogenous RNAs (ceRNAs) network [26]. In the present study, DIANA-LncBase v.2 tool was used to identify possible microRNAs that interacted with LINC01287 in colon cancer [27]. A series of microRNAs were selected according to our RNA-seq results. We overexpressed LINC01287 in SW480 and HCT116 cells, then evaluated the expression change of these microRNAs. As shown in Fig. 4A and B, the expression level of miR-4500 was dramatically influenced by LINC01287 overexpression. In addition, previous studies prove that miR-4500 acts a role in the initiation and progression of human cancers including colon cancer [28–30]. Besides, we found that silencing LINC01287 in SW480 and HCT116 cells aggrandized the expression of miR-4500 (Fig. 4C). Thus we speculated that LINC01287 might sponge miR-4500 in colon cancer cells. The potential binding sites of LINC01287 and miR-4500 was depicted in Fig. 4D. In luciferase reporter assay, forced miR-4500 expression reduced the luciferase activity of wild type (wt) LINC01287, but this was failed in the mt LINC01287 which had a 3-bp mutation in the predicted binding sites (Fig. 4E). In the pull down assay, the level of LINC01287 was significantly higher in biotin-labeled wt-miR-4500 group than biotin-labeled mt-miR-4500 group (Fig. 4F). Besides, LINC01287 level was negatively connected with miR-4500 expression in colon cancer patients (Fig. 4G). These results indicated that LINC01287 acted as a molecular sponge for miR-4500 in colon cancer.

### MAP3K13 is a target of miR-4500 and indirectly regulated by LINC01287

**Fig. 5:**
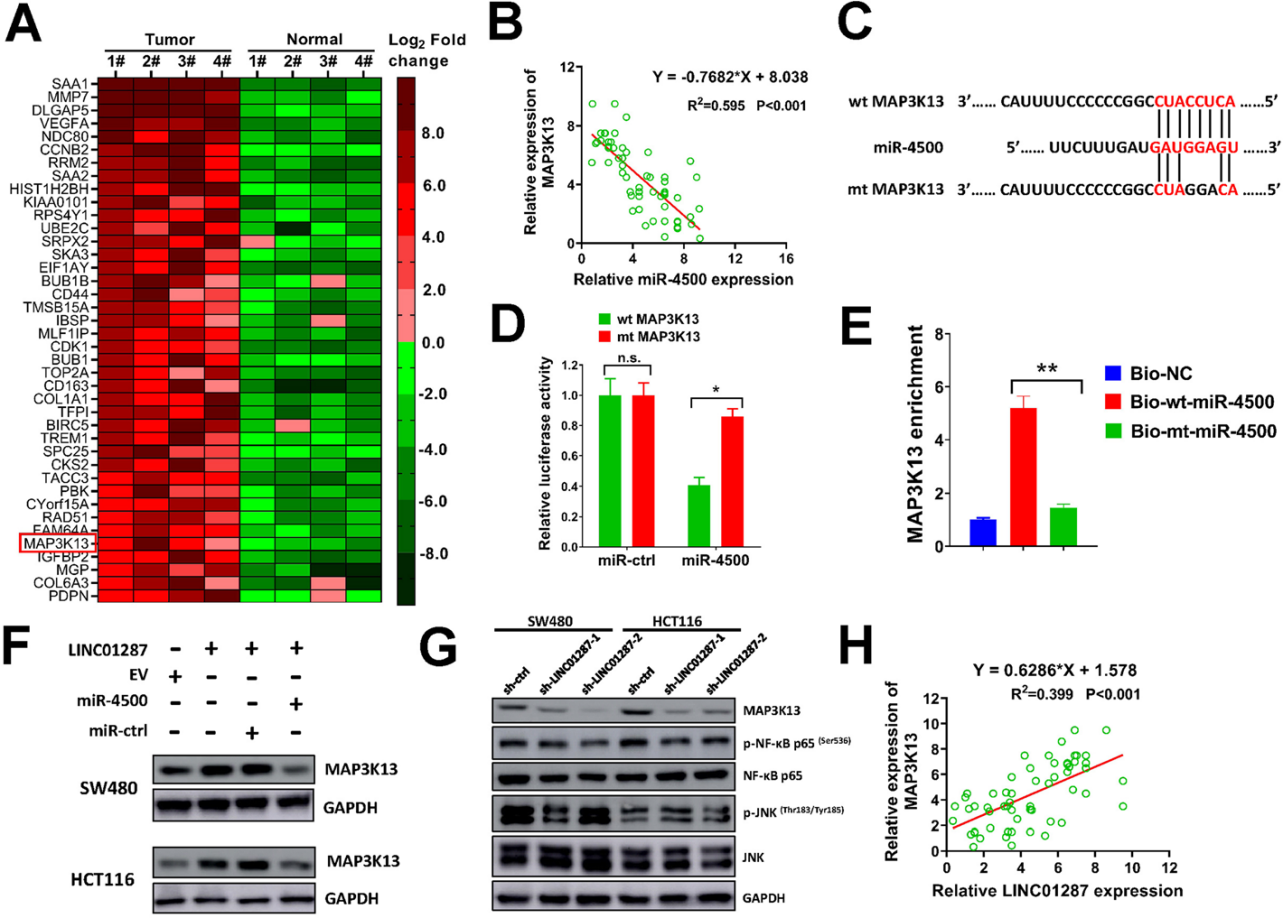
MAP3K13 is a target of miR-4500 and is indirectly regulated by LINC01287. **A**, heat map showed the top 40 upregulated mRNAs in 4 colon cancer tissues and paired normal samples identified by transcriptome sequencing. **B**, spearman analysis of correlation between MAP3K13 and miR-4500 expression in 60 colon cancer patients. **C**, the predicted binding sites of MAP3K13 and miR-4500. **D**, The pMIRREPORT plasmid containing the wt or mt 3′ UTR of MAP3K13, the miR-4500 expression plasmid or miR-ctrl, and a renilla luciferase plasmid (2: 2: 1) were transient transfected into HEK293T cells for luciferase reporter assay. E, the levels of MAP3K13 pulled down by biotinylated wt or mt miR-4500, or non-targeting control (NC) were tested by qRT-PCR. F, SW480 and HCT116 cells were infected with LINC01287, EV, miR-4500 or miR-ctrl lentiviral particles as indicated, then protein expression of MAP3K13 was tested by western blot. Uncropped gels were in Supplementary Fig. 1. **G**, SW480 and HCT116 cells were infected with sh-LINC01287–1, sh-LINC01287–2 or sh-ctrl lentiviral particles, then protein levels of indicated genes were tested by western blot. Uncropped gels were in Supplementary Fig. 2. **H**, spearman analysis of correlation between LINC01287 and MAP3K13 expression in 60 colon cancer patients. Compared with wt MAP3K13 or Bio-NC group using two-tailed Student’s t-test, *p ≤ 0.05, **p ≤ 0.001. n.s. = not significant

Accumulated studies demonstrate that microRNAs exert their functions by regulating downstream target genes. In our study, TargetScanHuman 7.2 was used to search potential target genes for miR-4500 [31]. Among all candidates, MAP3K13 drew our attention as it ranked the top 50 up-regulated mRNAs in the RNA-seq result of our study (Fig. 5A, Supplementary Table 2). Furthermore, previous reports suggest MAP3K13 exerts oncogenic roles in human cancers [15, 19, 20]. Notably, the expression of MAP3K13 was conversely correlated with miR-4500 expression in colon cancer patients (Fig. 5B). The putative binding sites for MAP3K13 and miR-4500 were depicted in Fig. 5C. In luciferase reporter assay, miR-4500 overexpression dramatically reduced the luciferase activity of wt MAP3K13, but this was abrogated in mt MAP3K13 which had a 3-bp mutation in the predicted binding sites (Fig. 5D). In RNA pull down assay, the level of MAP3K13 was significantly higher in biotin-labeled wt-miR-4500 group than biotin-labeled mt-miR-4500 group which had a 3-bp mutation in the predicted binding sites of MAP3K13 and miR-4500 (Fig. 5E). The interaction between LINC01287, miR-4500 and MAP3K13 was further validated by western blot. Our data indicated that LINC01287 overexpression upregulated MAP3K13 protein level, but this effect was attenuated by miR-4500 overexpression (Fig. 5F). MAP3K13 has been reported to activate both the NF-кB and JUN pathways [17, 18]. Our data proved that silencing LINC01287 inhibited MAP3K13 expression in SW480 and HCT116 cells, and subsequently reduced the phosphorylation of NF-кB p65 but not JNK (Fig. 5G). The correlation of LINC01287 and MAP3K13 was evaluated. Our results suggested that LINC01287 had a positively association with MAP3K13 level in colon cancer patients (Fig. 5H). These results indicated that LINC01287 activated NF-кB signaling but not JUN signaling in colon cancer cells by mediating MAP3K13 expression. Collectively, our data proved that MAP3K13 was a downstream target of miR-4500 and indirectly regulated by LINC01287.

### Restore MAP3K13 expression or miR-4500 knockdown partially reverses the effects caused by LINC01287 depletion in colon cancer cells

**Fig. 6:**
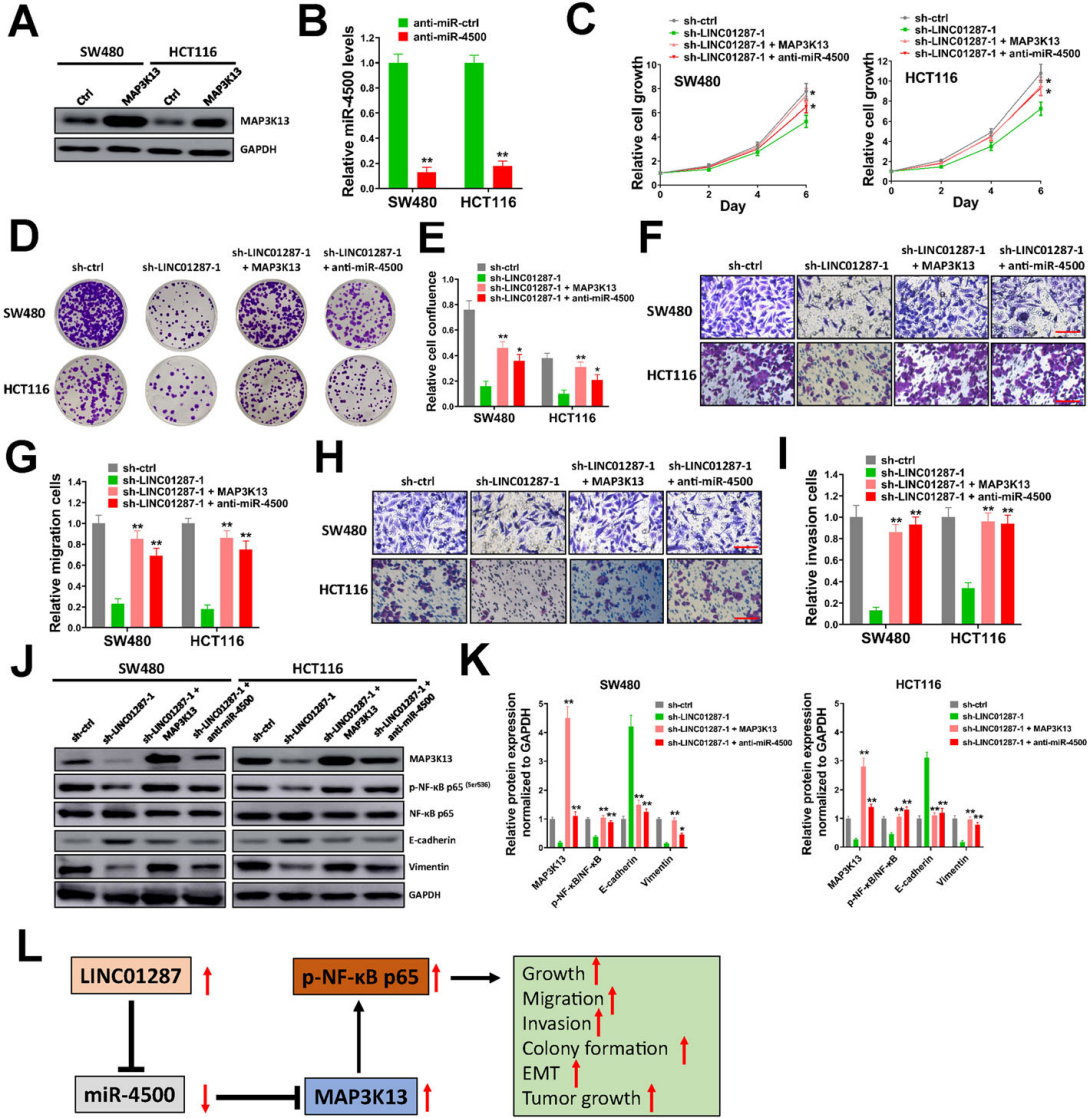
Restore MAP3K13 overexpression or miR-4500 knockdown partially reverses the effects caused by LINC01287 depletion in colon cancer cells. **A**, SW480 and HCT116 cells were infected with MAP3K13 or empty vector ctrl (Ctrl) lentiviral particles, then protein level of MAP3K13 was tested by western blot. Uncropped gels were in Supplementary Fig. 2. **B**, SW480 and HCT116 cells were infected with anti-miR-4500 or anti-miR-ctrl lentviral particles, then miR-4500 level was measured by qRT-PCR. **C**, SW480 and HCT116 cells were infected with sh-ctrl, sh-LINC01287–1, MAPK3K13 or anti-miR-4500 lentiviral particles as indicated, then seeded into 96-well plates (2500/well). Cell viability was tested at indicated time points. **D**-**E**, SW480 and HCT116 cells were infected with sh-ctrl, sh-LINC01287–1, MAPK3K13 or anti-miR-4500 lentiviral particles as indicated, then seeded into 6-well plates (1500/well) and grew for 2 weeks for colony formation assay. Representative images for colonies (**D**) and relative cell confluence of all colonies (**E**) were shown. **F**-**I**, SW480 and HCT116 cells were infected with sh-ctrl, sh-LINC01287–1, MAPK3K13 or anti-miR-4500 lentiviral particles as indicated, then used for transwell migration (**F**-**G**) or invasion assay (**H**-**I**). Representative images for migration cells (**F**) or invasion cells (**H**), and relative migration (**G**) and invasion (**I**) cells were shown. Scale bar = 50 μm. **J**-**K**. SW480 and HCT116 cells were infected with sh-ctrl, sh-LINC01287–1, MAPK3K13 or anti-miR-4500 lentiviral particles as indicated, then protein expression of indicated genes was tested by western blot (**J**). Uncropped gels were in Supplementary Fig. 2. Relative protein expression compared with GAPDH was shown (**K**). **L**, the potential mode of action of LINC01287 in colon cancer cells. Compared with anti-miR-ctrl or sh-LINC01287–1 group using two-tailed Student’s t-test, *p ≤ 0.05, **p ≤ 0.001

To prove whether LINC01287 exerted its oncogenic effects in colon cancer through miR-4500/MAP3K13 axis, MAP3K13 and anti-miR-4500 expression lentivirus vectors were constructed and rescue assays were performed in SW480 and HCT116 cells. In our study, transducing with MAP3K13 expression lentivirus significantly increased MAP3K13 expression (Fig. 6A). In addition, anti-miR-4500 successfully depleted miR-4500 expression in SW480 and HCT116 cells (Fig. 6B). Cell proliferation assay demonstrated that the decreased cell proliferation caused by LINC01287 knockdown was partially rescued by restoring MAP3K13 expression or miR-4500 knockdown (Fig. 6C). Similarly, colony formation assay revealed that silencing LINC01287 reduced colony formation of SW480 and HCT116 cells, however this effect was diminished by MAP3K13 overexpression or miR-4500 knockdown (Fig. 6D and E). In transwell migration and invasion assays, silencing LINC01287 evidently decreased the number of migration and invasion cells, however this was rescued by MAP3K13 overexpression or silencing miR-4500 (Fig. 6F-I). In addition, data from western blot analysis suggested that silencing LINC01287 reduced p-NF-кB p65 and Vimentin levels, and upregulated E-cadherin level in SW480 and HCT116 cells, but MAP3K13 overexpression or silencing miR-4500 countervailed these effects (Fig. 6J and K). The potential mode of action of LINC01287 in colon cancer cells was shown (Fig. 6L). Taken together, our results indicated that restored MAP3K13 expression or miR-4500 knockdown partially reversed the effects caused by LINC01287 depletion in colon cancer cells.

## Discussion

In the present study, LINC01287 was proved to up-regulate in colon cancer patients and high LINC01287 expression was connected with advanced TNM stage, lymph node metastasis and distant metastasis. Biological functional studies revealed that LINC01287 promoted proliferation, migration, invasion and EMT of colon cancer cells. Indeed, transcriptome sequencing of colon cancer samples in our study identified many dysregulated lncRNAs, some of which were proved to involve in the carcinogenesis of colon cancer. For example, CRNDE ranked the top 5 up-regulated lncRNAs in our study, and previous studies suggest that CRNDE overexpression in colon cancer cells promotes cell growth and chemoresistance of colon cancer cells by medicating Wnt/ß-catenin activation [22]. PVT1 is reported to facilitate cell growth and invasion of colon cancer cells via Lin28/let-7 axis [23]. LINC00152 is up-regulated and predicts poor prognosis of colon cancer patients [24]. Other examples, such as LEF1-AS1, H19, MEG3 and XIST were also identified by our sequencing and reported in other studies [32]. In our study, we focused on LINC01287 because this newly found lncRNA ranked the top 50 dyregulated lncRNAs and displayed biological activities in several other human malignancies. LINC01287 is increased in *H.pylori* positive gastric cancer patients and correlates with poor overall survival [13]. In lung cancer, LINC01287 is correlated with metastasis and can be prognostic maker for lung cancer patients [14]. Moreover, up-regulated LINC01287 contributes to growth and invasion of hepatocellular carcinoma cells through miR-298/MYB signaling [11]. LINC01287 is up-regulated in breast cancer patients and correlated with advanced TNM stage, lymph node metastasis and shorter overall survival [12]. Knockdown of LINC01287 in breast cancer cells inhibits proliferation and metastasis by regulating Wnt/ß-catenin signaling. These studies indicate that LINC01287 may act like oncogene in cancers. In our study, we demonstrated that LINC01287 overexpression facilitated proliferation, migration, invasion and EMT of colon cancer cells, and this might due to sponging miR-4500 and mediating MAP3K13 expression. Our results also demonstrated an oncogenic role of LINC01287 in colon cancer. In our study, we found that LINC01287 knockdown up-regulated the E-cadherin level and reduced the N-cadherin and Vimentin levels, while enforced LINC01287 expression showed contrary effects. These results indicated that LINC01287 might also facilitate EMT phenotype in colon cancer cells, thus promoted migration and invasion. Virtually, knockdown of LINC01287 upregulates E-cadherin level and reduces N-cadherin and Vimentin levels in hepatocellular carcinoma cells, indicating that LINC01287 is also involved in the EMT of hepatocellular carcinoma [10].

LncRNAs may act as competitive ceRNAs and negatively regulate microRNA expression. LINC01287 is demonstrated to mediate gene expression via sponging microRNAs [11, 33]. In our study, several microRNAs were predicted to interact with LINC01287 by DIANA-LncBase v.2 database. Among the identified microRNAs, miR-4500 was demonstrated to interact with LINC01287. In addition, miR-4500 was positively connected with LINC01287 expression in colon cancer patients. Previous studies have uncovered the tumor-suppressor role of miR-4500 in cancers. For example, miR-4500 overexpression inhibits growth, metastasis and EMT of hepatocellular cancer cells [34]. MiR-4500 is down-regulated in papillary thyroid cancer patients and cell lines, and associated with shorter survival, advanced tumor stage, and lymphatic metastasis [30]. Forced miR-4500 expression suppresses proliferation, colony formation, and invasion of papillary thyroid cancer cells. In non-small cell lung cancer, miR-4500 is decreased in patient samples and cell lines, while miR-4500 overexpression suppresses cell growth and metastasis, and facilitates cell apoptosis of non-small cell lung cancer cells [35, 36]. In breast cancer, miR-4500 expression is down-regulated, and exerts anti-cancer effects by attenuating cancer cell migration, invasion and capillary-like tube formation of endothelial cells through regulating RRM2-dependent MAPK signaling pathway [37]. The tumor suppressor function of miR-4500 is also demonstrated in bladder cancer and glioma [28, 38]. More importantly, previous studies demonstrate that miR-4500 is down-regulated in colon cancer patients and associated with higher tumor stage and shorter survival, while overexpression of miR-4500 suppresses cell proliferation, cell cycle progression and metastasis of colon cancer [29, 39]. Consistently, we found that LINC01287 promoted proliferation, migration and invasion by regulating miR-4500 in colon cancer cells, while miR-4500 knockdown reversed the effects caused by silencing LINC01287.

MAP3K13, a gene in MAPK signaling pathway, is proved to be oncogenic in human cancers [15, 20, 40]. In our study, we found that MAP3K13 ranked the top 50 up-regulated genes by transcriptome sequencing. Moreover, restoring MAP3K13 expression abolished the effects caused by LINC01287 knockdown, indicating the oncogenic functions of MAP3K13 in colon cancer cells. MAP3K13 plays a role in cancer, too. MAP3K13 is proved to be an amplified driver gene in head and neck squamous cell carcinoma (HNSCC). Silencing MAP3K13 suppresses proliferation, colony formation and tumor growth of HNSCC cells by stabilization of mutant p53 [19]. Moreover, MAP3K13 is demonstrate as a positive regulator of Myc oncogene, and promotes tumor development by stabilizing Myc through RIM25-FBXW7α signaling axis [40]. Previous studies suggest that MAP3K13 is participated in the activating of NF-кB and JUN pathways [17, 18]. Similarly, our results revealed that up-regulated LINC01287 increased MAP3K13 expression, and subsequently increased the phosphorylation of NF-кB p65, but not phosphorylated JNK, indicating that LINC01287 activated NF-кB signaling though regulating MAP3K13. More importantly, accumulated studies demonstrate that NF-кB signaling takes a part in regulating metastasis and EMT of cancer cells [41–43], thus we speculate that LINC01287 might regulate migration, invasion and EMT through NF-кB activation in colon cancer, but this need to be further investigated.

Potential limitations for our study include that the function of LINC01287 in colon cancer were only evaluated in cell lines and nude mice, and there is needing for study this in patient-derived xenografts or transgenic mice. In our study, the empty plasmid of pLKO.1 was used as sh-ctrl, but this was not as perfect for knockdown experiments. Moreover, we demonstrated that LINC01287 might regulate migration, invasion and EMT via NF-кB activation, but more evidences for this were needed to prove this in future study.

## Conclusions

In summary, up-regulated LINC01287 predicted poor prognosis of colon cancer patients. Silencing LINC01287 suppressed proliferation, migration, invasion and EMT of colon cancer cells, while enforced LINC01287 expression showed contrary effects. LINC01287 mediated MAP3K13 expression by sponging miR-4500, thus activated NF-кB signaling. Our results disclosed the oncogenic role of LINC01287 in colon cancer. Therefore, LINC01287 might be a potential therapeutic target and prognostic marker for colon cancer patients.

## Supplementary Information

**Additional file 1: Supplementary Table 1.** Differentially regulated lncRNAs between colon cancer tissues (CC) vs paired normal samples.

**Additional file 2: Supplementary Table 2.** Differentially regulated mRNAs between colon cancer tissues (CC) vs paired normal samples.

**Additional file 3.**

**Additional file 4.**

## Data Availability

The datasets used and/or analysed during the current study are available from the corresponding author on reasonable request.

## Abbreviations

lncRNAs: long noncoding RNAs
LINC01287: long intergenic non-protein coding RNA 1287
EMT: epithelial-mesenchymal transition
LZK: leucine Zipper-bearing Kinase
MAPKKK: mitogen-activated protein kinase kinase kinase
DLK/MAP3K12: Dual Leucine Zipper Kinase
ATCC: American Type Culture Collection
DSMZ: Deutsche Sammlung von Mikroorganismen und Zellkulturen
DEmRNAs: different expressed mRNAs
ceRNAs: competing endogenous RNAs
shRNA: short hairpin RNA
sgRNA: small guide RNA
