# Supplementary material for: LINC01287 facilitates proliferation, migration, invasion and EMT of colon cancer cells via miR-4500/MAP3K13 pathway
Source: BMC Cancer. 2021 Jul 6;21:782. doi: 10.1186/s12885-021-08528-7 (PMC8259379; doi:10.1186/s12885-021-08528-7)
Supplement: Supplementary file 1 — Additional file 1: Supplementary Table 1. Differentially regulated lncRNAs between colon cancer tissues (CC) vs paired normal samples. [file 12885_2021_8528_MOESM1_ESM.docx]

**Supplementary Table 1. Differentially regulated lncRNAs between colon cancer tissues (CC) vs paired normal samples.**

| **Probe Name** | **type** | **seqname** | **Gene Symbol** | **Log2 fold change** | **p-value** |
| --- | --- | --- | --- | --- | --- |
| ASHGA5P033644 | noncoding | ENST00000579923 | RP11-106E15.1 | 11.7076427 | 0.000000474 |
| ASHGA5P049223 | noncoding | ENST00000558952 | CRNDE | 10.3641529 | 2.31E-12 |
| ASHGA5P026381 | noncoding | ENST00000529081 | CTD-2026G22.1 | 9.9073429 | 0.00000626 |
| ASHGA5P035445 | noncoding | NR_038435 | HOXD-AS2 | 8.4634686 | 9.86E-16 |
| ASHGA5P049224 | noncoding | ENST00000558031 | CRNDE | 7.7599627 | 2.24E-11 |
| ASHGA5P045033 | noncoding | ENST00000522414 | PVT1 | 7.6204496 | 0.000000577 |
| ASHGA5P035444 | noncoding | uc002ukl.1 | AX747372 | 6.8861941 | 3.11E-18 |
| ASHGA5P007375 | noncoding | NR_024206 | LINC00152 | 6.4646244 | 0.00000178 |
| ASHGA5P041455 | noncoding | NR_038902 | JAKMIP2-AS1 | 6.1298285 | 0.000000681 |
| ASHGA5P042096 | noncoding | ENST00000455011 | RP3-460G2.2 | 6.0626322 | 0.000000924 |
| ASHGA5P042187 | noncoding | ENST00000424343 | RP11-503C24.2 | 5.9056615 | 0.000000245 |
| ASHGA5P016768 | noncoding | NR_038367 | HOTAIRM1 | 5.8484097 | 3.8E-09 |
| ASHGA5P023461 | noncoding | ENST00000581442 | RP11-838N2.4 | 5.7147819 | 5.59E-08 |
| ASHGA5P040239 | noncoding | ENST00000502941 | RP11-471J12.1 | 5.6722572 | 0.000003 |
| ASHGA5P036552 | noncoding | ENST00000413353 | AC019068.2 | 5.5345334 | 0.0000416 |
| ASHGA5P022599 | noncoding | ENST00000559432 | CRNDE | 5.5000462 | 6.52E-12 |
| ASHGA5P014767 | noncoding | ENST00000413202 | LINC00152 | 5.4520316 | 0.00000269 |
| ASHGA5P043787 | noncoding | ENST00000669109 | LINC01287 | 5.4253063 | 0.00000174 |
| ASHGA5P019706 | noncoding | uc010vhb.2 | CRNDE | 5.2414287 | 3.49E-12 |
| ASHGA5P054665 | noncoding | uc021qbz.1 | H19 | 5.1360087 | 0.00257 |
| ASHGA5P020249 | noncoding | ENST00000510339 | RP11-471J12.1 | 5.0946002 | 0.000000588 |
| ASHGA5P031786 | noncoding | ENST00000560487 | CTD-3032H12.2 | 5.0688009 | 2.62E-08 |
| ASHGA5P058334 | noncoding | uc001gzl.3 | BC034684 | 4.8467371 | 0.00000632 |
| ASHGA5P033084 | noncoding | TCONS_00028182 | XLOC_013541 | 4.7851695 | 2.44E-10 |
| ASHGA5P018095 | noncoding | ENST00000449386 | RP4-792G4.2 | 4.6941142 | 1.78E-12 |
| ASHGA5P020105 | noncoding | ENST00000508286 | LEF1-AS1 | 4.6592811 | 0.000000465 |
| ASHGA5P040408 | noncoding | ENST00000510137 | CTD-2263F21.1 | 4.6361353 | 0.000000275 |
| ASHGA5P043394 | noncoding | ENST00000517565 | RP11-61L23.2 | 4.5132295 | 6.56E-08 |
| ASHGA5P055907 | noncoding | ENST00000537850 | AE000661.37 | 4.5058338 | 0.00000563 |
| ASHGA5P040947 | noncoding | ENST00000506335 | CTD-2194D22.3 | 4.4847439 | 0.000697 |
| ASHGA5P019383 | noncoding | NR_046371 | CP | 4.3922022 | 0.0000532 |
| ASHGA5P021779 | noncoding | NR_037793 | LRR1 | 4.3102435 | 4.24E-10 |
| ASHGA5P026306 | noncoding | ENST00000510619 | RP1-68D18.2 | 4.2504366 | 0.000000552 |
| ASHGA5P050239 | noncoding | ENST00000426393 | RP4-792G4.2 | 4.2279539 | 9.06E-08 |
| ASHGA5P014724 | noncoding | uc001lva.4 | H19 | 4.2130266 | 0.0309 |
| ASHGA5P046821 | noncoding | NR_001545 | TTTY15 | 4.2119128 | 0.0158 |
| ASHGA5P046603 | noncoding | ENST00000568809 | RP11-524D16__A.3 | 4.1997486 | 0.000000313 |
| ASHGA5P045185 | noncoding | ENST00000437601 | RP4-781K5.8 | 4.1530927 | 0.00000025 |
| ASHGA5P042295 | noncoding | uc003nhj.3 | BC079832 | 4.1175995 | 8.28E-08 |
| ASHGA5P035263 | noncoding | ENST00000584273 | RP11-19E11.1 | 4.0845371 | 0.00000795 |
| ASHGA5P020639 | noncoding | ENST00000517838 | PVT1 | 4.0605522 | 2.46E-08 |
| ASHGA5P037416 | noncoding | NR_001458 | MIR155HG | 4.0228434 | 0.000000498 |
| ASHGA5P053704 | noncoding | NR_046175 | LOC286297 | 3.9932007 | 0.0000641 |
| ASHGA5P049942 | noncoding | uc002kmd.2 | AX721193 | 3.9811705 | 0.00000652 |
| ASHGA5P039177 | noncoding | ENST00000472856 | SOX2-OT | 3.9739477 | 0.00000256 |
| ASHGA5P018851 | noncoding | ENST00000458282 | RP11-189B4.6 | 3.9651273 | 0.000074 |
| ASHGA5P043890 | noncoding | ENST00000478818 | RP11-155G14.5 | 3.9171757 | 1.72E-08 |
| ASHGA5P049029 | noncoding | ENST00000573315 | LINC00514 | 3.8650923 | 0.0214 |
| ASHGA5P028584 | noncoding | ENST00000437267 | BX571672.2 | 3.8609779 | 0.000251 |
| ASHGA5P015153 | noncoding | ENST00000417355 | AC114803.3 | 3.8484791 | 0.0000246 |
| ASHGA5P043538 | noncoding | ENST00000417460 | AC003986.7 | 3.8380397 | 0.00000238 |
| ASHGA5P035083 | noncoding | ENST00000457448 | AC010987.6 | 3.6342762 | 0.0000122 |
| ASHGA5P052859 | noncoding | ENST00000466706 | TAAR3 | 3.6312179 | 0.0000474 |
| ASHGA5P029626 | noncoding | ENST00000553944 | ACTN1-AS1 | 3.6009382 | 0.000000156 |
| ASHGA5P057808 | noncoding | TCONS_00022112 | XLOC_010719 | 3.5926824 | 0.000355 |
| ASHGA5P017469 | noncoding | ENST00000442037 | H19 | 3.5858932 | 0.000434 |
| ASHGA5P039016 | noncoding | NR_002811 | NPHP3-AS1 | 3.5374937 | 0.00000128 |
| ASHGA5P015368 | noncoding | ENST00000419668 | AC002454.1 | 3.5258778 | 0.00021 |
| ASHGA5P032454 | noncoding | ENST00000441312 | AC015936.3 | 3.5249932 | 0.000000257 |
| ASHGA5P029269 | noncoding | ENST00000555001 | RP11-299L17.3 | 3.5169798 | 0.0000238 |
| ASHGA5P051762 | noncoding | ENST00000488647 | EGFEM1P | 3.5086279 | 2.13E-08 |
| ASHGA5P037636 | noncoding | ENST00000444114 | RP5-1172A22.1 | 3.5009106 | 0.00113 |
| ASHGA5P051474 | noncoding | ENST00000452738 | BX571672.2 | 3.4976777 | 0.0000197 |
| ASHGA5P030667 | noncoding | ENST00000560590 | RP11-307C19.2 | 3.4817134 | 0.00000136 |
| ASHGA5P054569 | noncoding | NR_029380 | FLJ41350 | 3.4803519 | 0.000409 |
| ASHGA5P018571 | noncoding | ENST00000455131 | LINC00152 | 3.4765243 | 0.00000282 |
| ASHGA5P018550 | noncoding | ENST00000454875 | TTTY14 | 3.4669098 | 0.000728 |
| ASHGA5P044535 | noncoding | NR_033916 | LOC728724 | 3.4554964 | 4.55E-09 |
| ASHGA5P055580 | noncoding | ENST00000547946 | RMST | 3.4486178 | 0.00000064 |
| ASHGA5P017086 | noncoding | ENST00000437561 | LINC00152 | 3.4439015 | 0.00000252 |
| ASHGA5P027793 | noncoding | ENST00000535078 | ABC12-49244600F4.3 | 3.4406495 | 0.000019 |
| ASHGA5P025851 | noncoding | NR_038975 | MIR181A2HG | 3.4395845 | 0.000000485 |
| ASHGA5P029134 | noncoding | TCONS_00010961 | XLOC_004881 | 3.4267523 | 2.93E-09 |
| ASHGA5P014771 | noncoding | uc003wog.3 | LOC154822 | 3.4234039 | 0.00234 |
| ASHGA5P040324 | noncoding | ENST00000569381 | RP11-315A16.1 | 3.4136564 | 0.000125 |
| ASHGA5P033885 | noncoding | ENST00000579651 | RP11-674P19.2 | 3.4029852 | 0.000000336 |
| ASHGA5P056422 | noncoding | TCONS_00004149 | XLOC_001991 | 3.3786519 | 0.000159 |
| ASHGA5P021698 | noncoding | uc001tfa.1 | RMST | 3.3724548 | 0.000000611 |
| ASHGA5P014500 | noncoding | NR_024373 | LOC541471 | 3.3717149 | 0.00000222 |
| ASHGA5P036245 | noncoding | ENST00000430320 | RP1-65J11.1 | 3.3580272 | 2.72E-12 |
| ASHGA5P054421 | noncoding | TCONS_00010962 | XLOC_004881 | 3.3148878 | 1.56E-08 |
| ASHGA5P056005 | noncoding | NR_026797 | LINC00520 | 3.3139218 | 0.0000312 |
| ASHGA5P029479 | noncoding | ENST00000547786 | NOVA1-AS1 | 3.2995265 | 0.000000784 |
| ASHGA5P053820 | noncoding | uc004bos.3 | AB074162 | 3.2974249 | 0.000000148 |
| ASHGA5P036094 | noncoding | ENST00000568143 | RP11-84D1.2 | 3.2862215 | 0.000086 |
| ASHGA5P027418 | noncoding | ENST00000548900 | RP11-1143G9.4 | 3.2834765 | 0.0000335 |
| ASHGA5P058362 | noncoding | uc001vih.1 | AK098560 | 3.27916 | 0.00119 |
| ASHGA5P035999 | noncoding | uc002suw.1 | BC016831 | 3.2456274 | 0.000486 |
| ASHGA5P046755 | noncoding | uc022cjh.1 | IGL@ | 3.2332281 | 0.0000759 |
| ASHGA5P044793 | noncoding | TCONS_00001093 | XLOC_000340 | 3.231176 | 5.27E-09 |
| ASHGA5P053422 | noncoding | ENST00000458220 | GAS5 | 3.2072846 | 3.94E-08 |
| ASHGA5P039698 | noncoding | ENST00000503140 | RP11-148L24.1 | 3.191048 | 0.000912 |
| ASHGA5P016522 | noncoding | ENST00000431294 | RP4-792G4.2 | 3.1850285 | 9.86E-09 |
| ASHGA5P043573 | noncoding | ENST00000519935 | HOXA-AS4 | 3.1813061 | 0.00515 |
| ASHGA5P034812 | noncoding | uc021vcr.1 | BC011243 | 3.1760513 | 1.7E-09 |
| ASHGA5P058482 | noncoding | uc004aep.1 | AQP7P1 | 3.1516503 | 0.000185 |
| ASHGA5P055971 | noncoding | TCONS_00025721 | XLOC_012542 | 3.1505702 | 6.2E-09 |
| ASHGA5P037293 | noncoding | uc002yup.1 | AX746823 | 3.1439561 | 0.000000906 |
| ASHGA5P015898 | noncoding | ENST00000424523 | AC002454.1 | 3.1298743 | 0.000161 |
| ASHGA5P018683 | noncoding | ENST00000456460 | BX004987.5 | 3.1290119 | 0.00125 |
| ASHGA5P034213 | noncoding | TCONS_00016514 | XLOC_007896 | 3.114652 | 0.0000243 |
| ASHGA5P014710 | noncoding | NR_034128 | LOC440900 | 3.1142685 | 0.000000413 |
| ASHGA5P031206 | noncoding | ENST00000416416 | RP11-576I22.2 | 3.1101393 | 8.4E-09 |
| ASHGA5P033672 | noncoding | TCONS_00028340 | XLOC_013688 | 3.1034323 | 3.23E-09 |
| ASHGA5P040412 | noncoding | ENST00000512519 | CTD-2127H9.1 | 3.0995686 | 0.0000001 |
| ASHGA5P020931 | noncoding | ENST00000522197 | HAS2-AS1 | 3.0952009 | 5.89E-08 |
| ASHGA5P016309 | noncoding | ENST00000429139 | MIR181A2HG | 3.0720962 | 0.000000518 |
| ASHGA5P030691 | noncoding | ENST00000561560 | RP11-358L4.1 | 3.0684348 | 0.00000351 |
| ASHGA5P037474 | noncoding | ENST00000428667 | AP000695.4 | 3.0594298 | 0.00000039 |
| ASHGA5P014708 | noncoding | ENST00000412669 | AC002456.2 | 3.0587347 | 4.85E-08 |
| ASHGA5P020802 | noncoding | ENST00000520043 | HAS2-AS1 | 3.0566257 | 1.65E-08 |
| ASHGA5P025784 | noncoding | NR_037636 | GJA9-MYCBP | 3.0469651 | 4.56E-10 |
| ASHGA5P020784 | noncoding | ENST00000519762 | RP11-909N17.3 | 3.035977 | 0.000156 |
| ASHGA5P042080 | noncoding | ENST00000448942 | RP11-356I2.4 | 3.032789 | 0.0000073 |
| ASHGA5P032148 | noncoding | NR_026951 | LINC00324 | 3.0201355 | 3.07E-10 |
| ASHGA5P032536 | noncoding | ENST00000508851 | RP11-893F2.9 | 3.020024 | 0.000000382 |
| ASHGA5P043030 | noncoding | NR_038276 | LOC100506895 | 3.0095693 | 0.0000156 |
| ASHGA5P044256 | noncoding | ENST00000522408 | RP11-109P6.2 | 3.0056119 | 0.00236 |
| ASHGA5P040177 | noncoding | ENST00000421640 | BX004987.5 | 3.0018974 | 0.0000214 |
| ASHGA5P037131 | noncoding | ENST00000427794 | RP5-907D15.2 | 2.9980358 | 0.000606 |
| ASHGA5P027792 | noncoding | ENST00000545775 | ABC12-49244600F4.3 | 2.9923479 | 0.000188 |
| ASHGA5P025948 | noncoding | NR_047562 | PDLIM3 | 2.9868211 | 0.00101 |
| ASHGA5P032633 | noncoding | ENST00000581549 | LINC00511 | 2.9865471 | 5.93E-09 |
| ASHGA5P057644 | noncoding | TCONS_00020322 | XLOC_009653 | 2.9851651 | 1.94E-09 |
| ASHGA5P042961 | noncoding | ENST00000523608 | RP1-170O19.17 | 2.9821675 | 0.00286 |
| ASHGA5P040464 | noncoding | ENST00000510261 | CTD-2116N20.1 | 2.9793202 | 0.00000236 |
| ASHGA5P044891 | noncoding | ENST00000565297 | RP11-79H23.3 | 2.9711814 | 0.000565 |
| ASHGA5P037288 | noncoding | NR_038974 | LOC100288432 | 2.7314861 | 0.000000475 |
| ASHGA5P041217 | noncoding | ENST00000504287 | CTD-2316B1.1 | 2.7269961 | 0.00398 |
| ASHGA5P015046 | noncoding | ENST00000415965 | AC002456.2 | 2.7201372 | 0.000000166 |
| ASHGA5P052140 | noncoding | ENST00000509640 | RP11-471J12.1 | 2.7132213 | 0.0000344 |
| ASHGA5P050948 | noncoding | NR_049777 | SRG7 | 2.7052811 | 0.000000637 |
| ASHGA5P044991 | noncoding | NR_002835 | HAS2-AS1 | 2.6977629 | 0.000000703 |
| ASHGA5P053792 | noncoding | ENST00000563434 | RP11-4O1.2 | 2.6977141 | 0.00000741 |
| ASHGA5P058403 | noncoding | uc002qnp.1 | BC036412 | 2.6874 | 5.25E-08 |
| ASHGA5P019446 | noncoding | ENST00000485035 | SOX2-OT | 2.6855349 | 0.000161 |
| ASHGA5P053180 | noncoding | ENST00000449361 | RP11-115N4.1 | 2.6854512 | 0.000000518 |
| ASHGA5P030403 | noncoding | NR_026771 | DKFZP434L187 | 2.6836638 | 0.000393 |
| ASHGA5P045814 | noncoding | ENST00000563268 | RP11-367F23.2 | 2.6795091 | 0.00106 |
| ASHGA5P032355 | noncoding | ENST00000425161 | RP11-343H5.6 | 2.6720863 | 0.000613 |
| ASHGA5P025785 | noncoding | NR_037637 | GJA9-MYCBP | 2.669046 | 1.21E-10 |
| ASHGA5P037804 | noncoding | ENST00000452326 | AC000067.1 | 2.6689351 | 0.00000378 |
| ASHGA5P054138 | noncoding | ENST00000424306 | RP11-308D16.4 | 2.6648956 | 0.000000196 |
| ASHGA5P035690 | noncoding | ENST00000455579 | AC010729.1 | 2.6593456 | 0.00000205 |
| ASHGA5P017713 | noncoding | NR_036693 | CLEC2D | 2.6590445 | 4.73E-09 |
| ASHGA5P028851 | noncoding | ENST00000418943 | RP11-473M10.3 | 2.6493088 | 0.00145 |
| ASHGA5P056008 | noncoding | TCONS_00025513 | XLOC_012324 | 2.6446066 | 0.00000272 |
| ASHGA5P041149 | noncoding | ENST00000457791 | PMCHL2 | 2.6384102 | 0.000219 |
| ASHGA5P054917 | noncoding | ENST00000537024 | SNHG1 | 2.6321929 | 0.000000581 |
| ASHGA5P020435 | noncoding | ENST00000513480 | CTD-2127H9.1 | 2.6214019 | 0.000465 |
| ASHGA5P016936 | noncoding | ENST00000435868 | GUSBP11 | 2.6177216 | 0.000525 |
| ASHGA5P035947 | noncoding | NR_024606 | LBX2-AS1 | 2.6043792 | 0.0000634 |
| ASHGA5P056653 | noncoding | TCONS_00006919 | XLOC_002730 | 2.5918491 | 0.00148 |
| ASHGA5P031297 | noncoding | ENST00000436656 | GAS5 | 2.5843258 | 0.00000942 |
| ASHGA5P026550 | noncoding | ENST00000421455 | RP4-668G5.1 | 2.5789379 | 0.000000344 |
| ASHGA5P033815 | noncoding | ENST00000573479 | AC021224.1 | 2.5680271 | 0.00000851 |
| ASHGA5P041707 | noncoding | TCONS_00020316 | XLOC_009649 | 2.5490302 | 0.00145 |
| ASHGA5P046820 | noncoding | ENST00000417071 | TTTY15 | 2.5481402 | 0.00526 |
| ASHGA5P052599 | noncoding | uc011dkm.2 | BTN2A3P | 2.5437265 | 0.000003 |
| ASHGA5P056667 | noncoding | TCONS_00007030 | XLOC_002900 | 2.5408371 | 0.000136 |
| ASHGA5P040610 | noncoding | ENST00000512693 | CTD-2587M2.1 | 2.5403404 | 0.0000954 |
| ASHGA5P017463 | noncoding | ENST00000441971 | AC002456.2 | 2.5342002 | 0.000000225 |
| ASHGA5P038998 | noncoding | ENST00000418242 | RP11-148B18.3 | 2.5327947 | 0.0000138 |
| ASHGA5P042488 | noncoding | ENST00000449928 | RP11-542F9.1 | 2.5321306 | 0.000185 |
| ASHGA5P048021 | noncoding | ENST00000528366 | RP4-607I7.1 | 2.5257772 | 0.00000656 |
| ASHGA5P035448 | noncoding | NR_040001 | LOC375295 | 2.5186895 | 0.00421 |
| ASHGA5P038524 | noncoding | NR_034007 | LOC339894 | 2.5171604 | 0.000307 |
| ASHGA5P051166 | noncoding | uc010gqe.2 | DKFZp586E1322 | 2.5166843 | 0.000134 |
| ASHGA5P001071 | noncoding | ENST00000566038 | RRN3P2 | 2.5129072 | 6.01E-08 |
| ASHGA5P032579 | noncoding | TCONS_00029064 | XLOC_013986 | 2.5021349 | 0.0000837 |
| ASHGA5P057461 | noncoding | TCONS_00017645 | XLOC_008317 | 2.5016628 | 0.00678 |
| ASHGA5P044770 | noncoding | ENST00000563059 | KB-1836B5.1 | 2.5012181 | 0.0000282 |
| ASHGA5P033793 | noncoding | NR_040033 | LOC729950 | 2.4932175 | 0.000012 |
| ASHGA5P045223 | noncoding | uc004abd.1 | DQ573539 | 2.4909057 | 0.000401 |
| ASHGA5P053918 | noncoding | ENST00000412485 | GS1-600G8.5 | 2.4877642 | 0.00054 |
| ASHGA5P041021 | noncoding | ENST00000423102 | PMCHL1 | 2.4840046 | 0.000188 |
| ASHGA5P023186 | noncoding | uc002kmi.3 | BC094703 | 2.4838099 | 0.00000073 |
| ASHGA5P021090 | noncoding | NR_038952 | LOC100506343 | 2.476506 | 0.00000263 |
| ASHGA5P037534 | noncoding | ENST00000413768 | AP000525.8 | 2.4727618 | 0.000536 |
| ASHGA5P026897 | noncoding | ENST00000531193 | RP11-680F20.6 | 2.4709574 | 0.0021 |
| ASHGA5P046113 | noncoding | ENST00000443965 | GS1-600G8.5 | 2.466521 | 0.000328 |
| ASHGA5P058022 | noncoding | TCONS_00025441 | XLOC_012259 | 2.4648885 | 8.55E-08 |
| ASHGA5P058630 | noncoding | uc011hzu.2 | HLA-DQB | 2.4604617 | 0.00000959 |
| ASHGA5P037133 | noncoding | ENST00000424205 | RP5-907D15.2 | 2.4554159 | 0.000723 |
| ASHGA5P056254 | noncoding | TCONS_00001489 | XLOC_000828 | 2.451708 | 5.53E-08 |
| ASHGA5P046716 | noncoding | ENST00000438239 | LL0XNC01-37G1.1 | 2.4516819 | 0.00000494 |
| ASHGA5P026282 | noncoding | NR_033971 | DKFZp686K1684 | 2.4473516 | 0.000402 |
| ASHGA5P045031 | noncoding | ENST00000522875 | PVT1 | 2.4301624 | 6.92E-08 |
| ASHGA5P053428 | noncoding | ENST00000449589 | GAS5 | 2.4279038 | 9.06E-08 |
| ASHGA5P039144 | noncoding | ENST00000484765 | RP11-368I23.2 | 2.4257896 | 0.0000167 |
| ASHGA5P055877 | noncoding | uc001vvl.4 | AK022914 | 2.4175701 | 0.00146 |
| ASHGA5P029482 | noncoding | ENST00000548335 | RP11-626P14.2 | 2.41562 | 0.00273 |
| ASHGA5P047350 | noncoding | TCONS_00014959 | XLOC_007038 | 2.4111475 | 0.000383 |
| ASHGA5P058481 | noncoding | uc004aem.1 | AQP7P1 | 2.4099385 | 0.00321 |
| ASHGA5P031130 | noncoding | ENST00000567422 | RP11-23E10.4 | 2.408044 | 0.0000515 |
| ASHGA5P043574 | noncoding | ENST00000520395 | HOXA11-AS | 2.4076956 | 0.000482 |
| ASHGA5P036562 | noncoding | TCONS_00025984 | XLOC_012324 | 2.4060683 | 0.000579 |
| ASHGA5P020502 | noncoding | ENST00000514836 | RP11-610O8.1 | 2.4055574 | 0.00141 |
| ASHGA5P019332 | noncoding | NR_045147 | ITGB3BP | 2.3963653 | 2.45E-10 |
| ASHGA5P046915 | noncoding | ENST00000451946 | RP11-174J11.1 | 2.3962849 | 0.000048 |
| ASHGA5P020436 | noncoding | ENST00000513492 | RP11-213H15.3 | 2.3944575 | 0.00018 |
| ASHGA5P015315 | noncoding | ENST00000419211 | AC015987.1 | 2.3937267 | 0.000355 |
| ASHGA5P039027 | noncoding | uc003erl.1 | AX746877 | 2.3875748 | 0.00000678 |
| ASHGA5P042691 | noncoding | TCONS_00014547 | XLOC_006924 | 2.3872767 | 5.56E-09 |
| ASHGA5P027824 | noncoding | ENST00000500682 | RP11-277P12.20 | 2.3868613 | 0.00109 |
| ASHGA5P058353 | noncoding | uc001pen.1 | AF086184 | 2.377795 | 0.0000348 |
| ASHGA5P027749 | noncoding | ENST00000513358 | RP11-253E3.3 | 2.3759143 | 0.000000442 |
| ASHGA5P044266 | noncoding | uc003xrx.3 | BC039537 | 2.3725831 | 1.78E-09 |
| ASHGA5P051935 | noncoding | uc003gwh.3 | BC025350 | 2.3708397 | 0.00746 |
| ASHGA5P026488 | noncoding | ENST00000538355 | RP11-783K16.5 | 2.3688039 | 0.000000126 |
| ASHGA5P036450 | noncoding | TCONS_00026354 | XLOC_012707 | 2.3616013 | 0.000000261 |
| ASHGA5P032913 | noncoding | ENST00000572818 | RP11-160E2.11 | 2.3573588 | 0.00032 |
| ASHGA5P043293 | noncoding | NR_002330 | ST7-AS1 | 2.3521312 | 0.00000994 |
| ASHGA5P037472 | noncoding | ENST00000454980 | AP000695.4 | 2.3429239 | 0.0000332 |
| ASHGA5P027767 | noncoding | ENST00000541782 | SCARNA10 | 2.3399785 | 0.0000187 |
| ASHGA5P035828 | noncoding | ENST00000444629 | AC007254.3 | 2.3332999 | 0.000233 |
| ASHGA5P027278 | noncoding | ENST00000552061 | RP3-405J10.3 | 2.32656 | 0.000261 |
| ASHGA5P045393 | noncoding | ENST00000449258 | RP11-406O23.2 | 2.3264629 | 0.0000424 |
| ASHGA5P030071 | noncoding | TCONS_00008016 | XLOC_003481 | 2.3253061 | 0.000000154 |
| ASHGA5P058458 | noncoding | uc003tda.1 | DPY19L1P2 | 2.3248625 | 0.000821 |
| ASHGA5P019002 | noncoding | NR_026665 | CD58 | 2.3184416 | 0.000307 |
| ASHGA5P016435 | noncoding | NR_038951 | LOC100506343 | 2.3060141 | 0.0000169 |
| ASHGA5P033398 | noncoding | ENST00000570512 | RP11-353N14.1 | 2.3027111 | 0.00000355 |
| ASHGA5P021210 | noncoding | ENST00000528437 | RP11-693N9.2 | 2.2953581 | 0.000000449 |
| ASHGA5P027255 | noncoding | ENST00000548382 | RP11-161H23.11 | 2.2913361 | 0.0000686 |
| ASHGA5P041906 | noncoding | ENST00000423099 | RP11-505P4.6 | 2.2910847 | 0.000103 |
| ASHGA5P040713 | noncoding | TCONS_00022113 | XLOC_010719 | 2.2888325 | 0.00412 |
| ASHGA5P044053 | noncoding | uc003won.1 | BC107568 | 2.2872492 | 0.000014 |
| ASHGA5P056371 | noncoding | TCONS_00003800 | XLOC_001595 | 2.2860105 | 0.0000713 |
| ASHGA5P015152 | noncoding | ENST00000417354 | DNM3OS | 2.2844581 | 0.0032 |
| ASHGA5P021967 | noncoding | ENST00000545800 | RP11-783K16.13 | 2.2799445 | 0.0000125 |
| ASHGA5P056290 | noncoding | TCONS_00001811 | XLOC_001195 | 2.2767781 | 0.0000218 |
| ASHGA5P036553 | noncoding | uc002vwe.1 | AK023507 | 2.2756571 | 7.66E-08 |
| ASHGA5P026546 | noncoding | ENST00000423403 | RP11-436K8.1 | 2.273913 | 0.000264 |
| ASHGA5P014763 | noncoding | ENST00000413121 | AC018730.1 | 2.2717325 | 0.0000852 |
| ASHGA5P031379 | noncoding | ENST00000563280 | RP11-463O9.5 | 2.2688637 | 0.0000851 |
| ASHGA5P017198 | noncoding | ENST00000438790 | RP1-65J11.1 | 2.2656724 | 0.000189 |
| ASHGA5P031766 | noncoding | ENST00000565308 | CTD-2119F7.2 | 2.2568337 | 0.0197 |
| ASHGA5P019521 | noncoding | NR_027783 | SAT1 | 2.2541734 | 0.00605 |
| ASHGA5P005966 | noncoding | NR_047546 | SCFD1 | 2.249688 | 0.00104 |
| ASHGA5P053188 | noncoding | ENST00000435695 | AC002454.1 | 2.2488826 | 0.00296 |
| ASHGA5P038134 | noncoding | ENST00000426702 | UBE2E1-AS1 | 2.2482563 | 0.00000182 |
| ASHGA5P039176 | noncoding | uc003fkv.3 | SOX2-OT | 2.2450562 | 0.00659 |
| ASHGA5P031616 | noncoding | ENST00000565747 | CTD-2270L9.4 | 2.2428537 | 0.000000469 |
| ASHGA5P019948 | noncoding | ENST00000505700 | HOXC-AS1 | 2.2411202 | 0.00153 |
| ASHGA5P043579 | noncoding | ENST00000472494 | HOTTIP | 2.2398531 | 0.00322 |
| ASHGA5P055194 | noncoding | TCONS_00006915 | XLOC_002730 | 2.2372988 | 0.00589 |
| ASHGA5P021195 | noncoding | ENST00000527617 | RP11-693N9.2 | 2.2179372 | 0.000000181 |
| ASHGA5P043717 | noncoding | NR_037776 | WBSCR22 | 2.2177805 | 2.46E-08 |
| ASHGA5P044042 | noncoding | NR_038835 | LOC645249 | 2.2170675 | 0.000454 |
| ASHGA5P047233 | noncoding | ENST00000440436 | RP11-536K7.5 | 2.2145347 | 0.0000163 |
| ASHGA5P044321 | noncoding | ENST00000562490 | RP11-102F4.3 | 2.2136145 | 0.00379 |
| ASHGA5P022359 | noncoding | ENST00000555186 | CHEK2P2 | 2.208799 | 0.00544 |
| ASHGA5P038590 | noncoding | ENST00000489016 | RP11-139K4.2 | 2.2085423 | 0.000426 |
| ASHGA5P045916 | noncoding | ENST00000428292 | RP11-18B16.2 | 2.2023712 | 0.000943 |
| ASHGA5P039491 | noncoding | ENST00000507894 | RP11-742B18.1 | 2.2009181 | 0.000698 |
| ASHGA5P039363 | noncoding | NR_037877 | LOC100505912 | 2.1906867 | 0.00000154 |
| ASHGA5P051081 | noncoding | ENST00000453910 | LINC00478 | 2.1881908 | 0.00000121 |
| ASHGA5P052276 | noncoding | ENST00000510264 | RP11-848G14.2 | 2.1879412 | 0.0000205 |
| ASHGA5P043018 | noncoding | ENST00000415237 | AC004988.1 | 2.1847708 | 0.0002 |
| ASHGA5P043138 | noncoding | uc003ubz.1 | PMS2L14 | 2.1830608 | 0.0197 |
| ASHGA5P039618 | noncoding | ENST00000514727 | PPP1R14BP3 | 2.1768655 | 3.98E-08 |
| ASHGA5P020761 | noncoding | ENST00000519481 | PVT1 | 2.1761243 | 0.0000101 |
| ASHGA5P020340 | noncoding | ENST00000511918 | RP11-213H15.3 | 2.173146 | 0.00191 |
| ASHGA5P034297 | noncoding | ENST00000562112 | NAPSB | 2.1659254 | 0.00032 |
| ASHGA5P019192 | noncoding | NR_028444 | PDIA5 | 2.1634928 | 0.00127 |
| ASHGA5P025673 | noncoding | NR_024515 | APLP2 | 2.1625035 | 0.00573 |
| ASHGA5P037493 | noncoding | TCONS_00024509 | XLOC_011826 | 2.1612025 | 0.00173 |
| ASHGA5P046424 | noncoding | ENST00000430562 | CD99P1 | 2.1587895 | 0.00000161 |
| ASHGA5P036098 | noncoding | NR_026821 | FAM138B | 2.1585258 | 0.0184 |
| ASHGA5P023479 | noncoding | ENST00000581801 | LINC00511 | 2.1564113 | 0.00000377 |
| ASHGA5P016519 | noncoding | ENST00000450692 | AC009948.3 | 2.1468641 | 0.00000031 |
| ASHGA5P050525 | noncoding | ENST00000439192 | DGUOK-AS1 | 2.1426672 | 0.0001 |
| ASHGA5P045066 | noncoding | ENST00000439341 | RP5-858B6.3 | 2.1365539 | 0.00055 |
| ASHGA5P019939 | noncoding | NR_003679 | NBLA00301 | 2.1329951 | 0.000468 |
| ASHGA5P033837 | noncoding | TCONS_00003974 | XLOC_001788 | 2.1316642 | 0.000236 |
| ASHGA5P023153 | noncoding | NR_027683 | RPAIN | 2.1282465 | 8.28E-10 |
| ASHGA5P021729 | noncoding | NR_027682 | RPAIN | 2.1281429 | 0.000000386 |
| ASHGA5P056652 | noncoding | TCONS_00006918 | XLOC_002730 | 2.1273922 | 0.00501 |
| ASHGA5P036075 | noncoding | ENST00000455309 | AC017002.1 | 2.124567 | 0.0000165 |
| ASHGA5P015871 | noncoding | ENST00000424308 | RP4-794H19.4 | 2.1210376 | 0.00000236 |
| ASHGA5P028354 | noncoding | ENST00000456414 | RP5-1086K13.1 | 2.1205425 | 8.89E-08 |
| ASHGA5P037055 | noncoding | uc021wdt.1 | AK023614 | 2.1108077 | 0.0000238 |
| ASHGA5P028974 | noncoding | uc010ahe.1 | BC041856 | 2.1103512 | 0.00754 |
| ASHGA5P041087 | noncoding | uc003jox.1 | AK097288 | 2.1099607 | 0.00396 |
| ASHGA5P051151 | noncoding | uc002zdj.1 | DQ577420 | 2.1067509 | 0.000000585 |
| ASHGA5P038623 | noncoding | NR_033944 | LOC647323 | 2.1054752 | 0.000575 |
| ASHGA5P006177 | noncoding | NR_034086 | LOC648987 | 2.1046319 | 0.000000194 |
| ASHGA5P019785 | noncoding | ENST00000503152 | CTD-2201E18.3 | 2.1029357 | 0.000000338 |
| ASHGA5P026287 | noncoding | ENST00000459866 | WT1-AS | 2.099608 | 0.013 |
| ASHGA5P015438 | noncoding | ENST00000420221 | AC108025.2 | 2.093006 | 0.00043 |
| ASHGA5P014540 | noncoding | ENST00000409898 | LINC00152 | 2.0835183 | 0.000497 |
| ASHGA5P016501 | noncoding | uc010lgv.1 | PMS2P1 | 2.082825 | 1.22E-10 |
| ASHGA5P047444 | noncoding | NR_027182 | LOC84989 | 2.0822759 | 0.0000121 |
| ASHGA5P048417 | noncoding | ENST00000452503 | SKINTL | 2.0794745 | 0.00068 |
| ASHGA5P016541 | noncoding | ENST00000431464 | RP11-308D16.4 | 2.0734087 | 0.0000345 |
| ASHGA5P033616 | noncoding | uc002lfu.1 | AK093940 | 2.073077 | 0.000351 |
| ASHGA5P030737 | noncoding | ENST00000567484 | RP11-429B14.4 | 2.0711027 | 0.000399 |
| ASHGA5P020267 | noncoding | ENST00000510667 | RP11-438E5.1 | 2.0703142 | 0.00247 |
| ASHGA5P050391 | noncoding | ENST00000414661 | AC064875.2 | 2.0693552 | 0.00243 |
| ASHGA5P047160 | noncoding | ENST00000420845 | RP11-4C20.3 | 2.0664293 | 0.00387 |
| ASHGA5P018109 | noncoding | ENST00000449527 | BX571672.5 | 2.0643268 | 0.00108 |
| ASHGA5P037855 | noncoding | ENST00000444093 | KB-318B8.7 | 2.0624241 | 0.00743 |
| ASHGA5P027535 | noncoding | ENST00000551299 | RP11-341G23.4 | 2.0619069 | 0.0000532 |
| ASHGA5P018940 | noncoding | ENST00000475441 | SNHG12 | 2.0611149 | 2.69E-10 |
| ASHGA5P035114 | noncoding | ENST00000413452 | DGUOK-AS1 | 2.0601748 | 0.000157 |
| ASHGA5P042167 | noncoding | ENST00000564697 | RP1-155D22.2 | 2.0552642 | 0.000118 |
| ASHGA5P053878 | noncoding | uc004cib.1 | AX747706 | 2.0505574 | 0.00000605 |
| ASHGA5P018847 | noncoding | ENST00000458253 | AC018730.1 | 2.0480716 | 0.000384 |
| ASHGA5P039084 | noncoding | NR_036538 | LOC646903 | 2.0474468 | 0.0000535 |
| ASHGA5P045717 | noncoding | uc004aaa.3 | BC014180 | 2.0469269 | 0.00000172 |
| ASHGA5P020091 | noncoding | ENST00000508015 | TMEM161B-AS1 | 2.0436962 | 0.00351 |
| ASHGA5P018160 | noncoding | ENST00000450133 | RP9P | 2.0408881 | 0.000691 |
| ASHGA5P053487 | noncoding | ENST00000519854 | RP11-37B2.1 | 2.0374369 | 0.000000691 |
| ASHGA5P032388 | noncoding | ENST00000581080 | CTD-2267D19.2 | 2.0349698 | 0.000000379 |
| ASHGA5P042449 | noncoding | ENST00000573382 | RP1-302G2.5 | 2.0329311 | 0.000137 |
| ASHGA5P019840 | noncoding | ENST00000503938 | AC006445.8 | 2.0325966 | 0.00678 |
| ASHGA5P041077 | noncoding | ENST00000511712 | RP11-53O19.2 | 2.0290813 | 0.00413 |
| ASHGA5P038628 | noncoding | ENST00000415869 | RP11-513G11.4 | 2.0257364 | 0.000233 |
| ASHGA5P043780 | noncoding | ENST00000418395 | RP11-115N4.1 | 2.0252331 | 0.000264 |
| ASHGA5P048557 | noncoding | uc001ytj.3 | HERC2P7 | 2.0239835 | 0.00000153 |
| ASHGA5P019385 | noncoding | ENST00000481220 | SNHG12 | 2.023584 | 9.94E-10 |
| ASHGA5P058466 | noncoding | uc003txt.3 | BC018166 | 2.0224909 | 7.41E-08 |
| ASHGA5P020172 | noncoding | NR_038342 | LOC100505875 | 2.0221929 | 0.0186 |
| ASHGA5P045633 | noncoding | ENST00000450445 | RP11-62F24.2 | 2.0213216 | 0.00111 |
| ASHGA5P030441 | noncoding | ENST00000557883 | RP11-1008C21.1 | 2.0207593 | 0.0000464 |
| ASHGA5P017716 | noncoding | ENST00000445003 | RP11-290F20.3 | 2.0175914 | 0.0000071 |
| ASHGA5P018002 | noncoding | NR_026937 | OXSM | 2.0163749 | 0.00135 |
| ASHGA5P040917 | noncoding | TCONS_00021963 | XLOC_010561 | -2.0161946 | 0.00000454 |
| ASHGA5P058780 | noncoding | uc.432+ | uc.432 | -2.016905 | 0.00000721 |
| ASHGA5P028021 | noncoding | ENST00000566942 | RP11-284N8.3 | -2.0183534 | 0.0102 |
| ASHGA5P032459 | noncoding | ENST00000452741 | AC002117.1 | -2.0195208 | 4.07E-08 |
| ASHGA5P035795 | noncoding | NR_027099 | LINC00486 | -2.0201275 | 0.000096 |
| ASHGA5P027445 | noncoding | ENST00000552290 | RP11-114H23.1 | -2.0228337 | 0.000000841 |
| ASHGA5P040420 | noncoding | uc003jms.1 | AK000840 | -2.0237796 | 0.000407 |
| ASHGA5P019786 | noncoding | ENST00000503167 | RP11-168A11.1 | -2.0276641 | 0.00000158 |
| ASHGA5P026895 | noncoding | ENST00000583165 | RP11-680F20.11 | -2.0280434 | 0.00472 |
| ASHGA5P056876 | noncoding | TCONS_00010124 | XLOC_004590 | -2.0283095 | 0.000487 |
| ASHGA5P041549 | noncoding | ENST00000518605 | CTB-164N12.1 | -2.0294327 | 0.000218 |
| ASHGA5P035059 | noncoding | uc002sbr.3 | BC071802 | -2.0326208 | 4.22E-08 |
| ASHGA5P044023 | noncoding | NR_038926 | PRKAG2-AS1 | -2.033736 | 0.00000346 |
| ASHGA5P050292 | noncoding | NR_024330 | LINC00085 | -2.0338783 | 0.000000274 |
| ASHGA5P015840 | noncoding | uc021sbw.1 | MEG3 | -2.0343116 | 0.000246 |
| ASHGA5P050661 | noncoding | NR_036499 | LOC389043 | -2.0360111 | 4.44E-08 |
| ASHGA5P044889 | noncoding | ENST00000520603 | RP11-91P17.1 | -2.0368961 | 0.00000011 |
| ASHGA5P006333 | noncoding | NR_027148 | MIR7-3HG | -2.0370099 | 0.000232 |
| ASHGA5P016719 | noncoding | ENST00000433357 | RP11-255A11.21 | -2.0378848 | 0.0000218 |
| ASHGA5P037109 | noncoding | ENST00000413070 | RP4-715N11.2 | -2.0400925 | 0.0000573 |
| ASHGA5P047014 | noncoding | ENST00000451364 | RP11-429G19.2 | -2.0418682 | 0.000021 |
| ASHGA5P041822 | noncoding | ENST00000373171 | RP11-552E20.3 | -2.0435498 | 0.00000767 |
| ASHGA5P051015 | noncoding | uc021weo.1 | BC100777 | -2.0439959 | 0.000000195 |
| ASHGA5P034649 | noncoding | ENST00000425982 | AC005392.13 | -2.0441423 | 0.0000662 |
| ASHGA5P057244 | noncoding | TCONS_00014916 | XLOC_006983 | -2.0464541 | 0.0000411 |
| ASHGA5P035720 | noncoding | ENST00000432665 | LINC00570 | -2.0464974 | 0.00000536 |
| ASHGA5P028410 | noncoding | TCONS_00010556 | XLOC_005083 | -2.0478793 | 0.000173 |
| ASHGA5P015881 | noncoding | ENST00000424371 | EMX2OS | -2.0480889 | 0.00279 |
| ASHGA5P029094 | noncoding | ENST00000556024 | RP11-356O9.2 | -2.0490513 | 0.0000176 |
| ASHGA5P045688 | noncoding | TCONS_00001854 | XLOC_001242 | -2.0498323 | 0.0000055 |
| ASHGA5P047443 | noncoding | ENST00000428581 | RP11-444I9.3 | -2.0505892 | 0.00000524 |
| ASHGA5P050388 | noncoding | ENST00000453100 | LINC00570 | -2.0535499 | 0.00000234 |
| ASHGA5P047013 | noncoding | NR_038444 | LOC728558 | -2.0535612 | 4.63E-09 |
| ASHGA5P000364 | noncoding | chr2:60308225-60325275+ | chr2:60308225-60325275 | -2.0548086 | 0.00000968 |
| ASHGA5P057687 | noncoding | TCONS_00020732 | XLOC_010031 | -2.0554102 | 0.0000235 |
| ASHGA5P022539 | noncoding | ENST00000558556 | CTD-2008A1.2 | -2.0563358 | 0.0000572 |
| ASHGA5P028752 | noncoding | ENST00000453470 | LINC00297 | -2.0573971 | 0.000000977 |
| ASHGA5P046704 | noncoding | ENST00000423819 | RP11-297A16.2 | -2.0592018 | 0.000000634 |
| ASHGA5P018461 | noncoding | ENST00000453832 | MIR4500HG | -2.0594656 | 3.31E-09 |
| ASHGA5P037060 | noncoding | ENST00000430025 | RP1-269M15.3 | -2.0618936 | 0.00000342 |
| ASHGA5P033336 | noncoding | ENST00000577573 | RP11-1124B17.1 | -2.0623622 | 0.00000164 |
| ASHGA5P018171 | noncoding | ENST00000450217 | TSSC2 | -2.0630347 | 0.000000752 |
| ASHGA5P030066 | noncoding | TCONS_00008011 | XLOC_003475 | -2.066947 | 0.000405 |
| ASHGA5P020920 | noncoding | ENST00000521975 | AB015752.3 | -2.0675198 | 2.53E-09 |
| ASHGA5P029282 | noncoding | ENST00000380722 | RP11-497E19.1 | -2.069831 | 0.00000469 |
| ASHGA5P055077 | noncoding | ENST00000577699 | RP11-201M22.1 | -2.0712737 | 0.000000968 |
| ASHGA5P056427 | noncoding | TCONS_00004172 | XLOC_002012 | -2.0714727 | 0.00197 |
| ASHGA5P018248 | noncoding | ENST00000451176 | RP4-756G23.5 | -2.0737481 | 0.000000569 |
| ASHGA5P021065 | noncoding | ENST00000524359 | CTD-2272D18.1 | -2.0741764 | 8.49E-09 |
| ASHGA5P038847 | noncoding | uc003dlo.3 | LOC285401 | -2.0741995 | 0.000296 |
| ASHGA5P037011 | noncoding | ENST00000562532 | RP1-310O13.12 | -2.0744724 | 0.0108 |
| ASHGA5P043292 | noncoding | ENST00000421965 | AC006159.4 | -2.0746914 | 0.00014 |
| ASHGA5P015001 | noncoding | ENST00000415573 | RP1-163G9.1 | -2.074795 | 0.000000209 |
| ASHGA5P045981 | noncoding | TCONS_00001581 | XLOC_000915 | -2.0783648 | 0.0000124 |
| ASHGA5P027341 | noncoding | ENST00000554022 | RP11-973D8.4 | -2.0786369 | 4.09E-08 |
| ASHGA5P033299 | noncoding | uc002jcf.3 | TCAM1P | -2.0795284 | 0.000000321 |
| ASHGA5P051446 | noncoding | NR_036540 | LINC00622 | -2.0815761 | 0.000000859 |
| ASHGA5P057484 | noncoding | TCONS_00018219 | XLOC_008498 | -2.0819681 | 0.00242 |
| ASHGA5P037398 | noncoding | ENST00000416002 | AF240627.2 | -2.0822068 | 0.0000203 |
| ASHGA5P023795 | noncoding | HMlincRNA447- | HMlincRNA447 | -2.0824004 | 0.0000414 |
| ASHGA5P014872 | noncoding | ENST00000414198 | AC144521.1 | -2.0825986 | 0.000766 |
| ASHGA5P038164 | noncoding | ENST00000424886 | AC105749.5 | -2.0846784 | 0.0000155 |
| ASHGA5P019575 | noncoding | ENST00000493239 | RP11-61L23.2 | -2.0869539 | 1.79E-08 |
| ASHGA5P033034 | noncoding | ENST00000584721 | RP11-227G15.8 | -2.0879325 | 0.000493 |
| ASHGA5P016257 | noncoding | ENST00000428649 | FAM75C2 | -2.090219 | 0.00000154 |
| ASHGA5P028638 | noncoding | ENST00000449881 | UGGT2-IT1 | -2.0912306 | 0.000214 |
| ASHGA5P042940 | noncoding | ENST00000439839 | AC004485.3 | -2.0936876 | 0.000000532 |
| ASHGA5P052179 | noncoding | uc010ith.1 | LOC728613 | -2.0941251 | 0.00000003 |
| ASHGA5P029177 | noncoding | ENST00000555156 | RP11-355I22.4 | -2.095259 | 4.76E-08 |
| ASHGA5P027686 | noncoding | ENST00000545739 | RP11-955H22.3 | -2.0963478 | 0.00000906 |
| ASHGA5P035244 | noncoding | ENST00000446590 | AC016683.5 | -2.0965489 | 0.00000085 |
| ASHGA5P021015 | noncoding | ENST00000523422 | RP11-1C8.4 | -2.0969889 | 0.000000488 |
| ASHGA5P016422 | noncoding | ENST00000430247 | AF131217.1 | -2.0971875 | 0.00051 |
| ASHGA5P033007 | noncoding | ENST00000577420 | RP11-354P11.2 | -2.097201 | 0.00042 |
| ASHGA5P019974 | noncoding | ENST00000506106 | CTD-2023N9.1 | -2.098193 | 0.000000624 |
| ASHGA5P048442 | noncoding | uc001pnk.1 | AY364433 | -2.0986648 | 0.00000018 |
| ASHGA5P034868 | noncoding | ENST00000442956 | LINC00299 | -2.0995015 | 0.0000482 |
| ASHGA5P032053 | noncoding | TCONS_00029847 | XLOC_014288 | -2.0998382 | 0.00000114 |
| ASHGA5P015957 | noncoding | ENST00000425194 | RP13-614K11.2 | -2.0999719 | 0.000052 |
| ASHGA5P037752 | noncoding | ENST00000421538 | RP11-398F12.1 | -2.1003277 | 4.94E-09 |
| ASHGA5P046340 | noncoding | ENST00000445415 | AC004383.4 | -2.1045826 | 1.21E-10 |
| ASHGA5P036604 | noncoding | TCONS_00026418 | XLOC_012763 | -2.1047333 | 0.00000203 |
| ASHGA5P045825 | noncoding | ENST00000460854 | ANKRD19P | -2.1070705 | 2.38E-10 |
| ASHGA5P048157 | noncoding | ENST00000543907 | RP11-881M11.4 | -2.1071582 | 0.0000667 |
| ASHGA5P046474 | noncoding | TCONS_00001417 | XLOC_000736 | -2.1102745 | 0.00000386 |
| ASHGA5P056363 | noncoding | TCONS_00003731 | XLOC_001526 | -2.1111195 | 0.000000285 |
| ASHGA5P015839 | noncoding | NR_046465 | MEG3 | -2.1123738 | 0.00271 |
| ASHGA5P043244 | noncoding | ENST00000451953 | RP5-1059M17.1 | -2.1140286 | 0.000393 |
| ASHGA5P046355 | noncoding | NR_026935 | LOC158696 | -2.1160429 | 0.00000968 |
| ASHGA5P044385 | noncoding | ENST00000517884 | RP11-662G23.1 | -2.1182469 | 0.000157 |
| ASHGA5P039847 | noncoding | TCONS_00022822 | XLOC_011094 | -2.1186657 | 0.000000222 |
| ASHGA5P058414 | noncoding | uc003adh.3 | BC015159 | -2.120202 | 0.0000406 |
| ASHGA5P057977 | noncoding | TCONS_00024600 | XLOC_011893 | -2.123197 | 0.00000405 |
| ASHGA5P034986 | noncoding | NR_028386 | LOC375196 | -2.124211 | 0.000000151 |
| ASHGA5P028658 | noncoding | ENST00000416009 | CLYBL-AS1 | -2.1244477 | 0.00000349 |
| ASHGA5P017795 | noncoding | ENST00000445836 | TMEM191A | -2.126571 | 0.000000118 |
| ASHGA5P035815 | noncoding | ENST00000431999 | CYP1B1-AS1 | -2.1297786 | 0.000000642 |
| ASHGA5P035593 | noncoding | TCONS_00015979 | XLOC_007354 | -2.1326287 | 0.000024 |
| ASHGA5P030362 | noncoding | uc010ayj.2 | HBT8 | -2.1334898 | 0.0000124 |
| ASHGA5P031530 | noncoding | ENST00000569218 | RP11-895K13.2 | -2.1342548 | 0.00000869 |
| ASHGA5P045834 | noncoding | ENST00000425090 | RP11-526D8.7 | -2.1386752 | 0.00000248 |
| ASHGA5P031106 | noncoding | ENST00000565692 | CTD-2014E2.5 | -2.1396812 | 0.000122 |
| ASHGA5P032482 | noncoding | ENST00000570379 | RP11-156P1.3 | -2.1413397 | 0.0000015 |
| ASHGA5P058614 | noncoding | uc011blf.2 | COL6A4P2 | -2.1419148 | 0.0000254 |
| ASHGA5P019440 | noncoding | uc003dwf.4 | LOC100302640 | -2.1438194 | 0.000083 |
| ASHGA5P052545 | noncoding | uc003mmk.4 | LOC729678 | -2.1452769 | 0.0000821 |
| ASHGA5P041900 | noncoding | ENST00000413945 | LINC00472 | -2.1458604 | 2.21E-10 |
| ASHGA5P028440 | noncoding | ENST00000413501 | RP11-165I9.4 | -2.146802 | 0.000762 |
| ASHGA5P049160 | noncoding | ENST00000566144 | RP11-455F5.5 | -2.1494398 | 0.00000541 |
| ASHGA5P030644 | noncoding | ENST00000418602 | RP11-536C5.7 | -2.1527652 | 0.00000777 |
| ASHGA5P044486 | noncoding | uc022baa.1 | AL832163 | -2.1528622 | 0.000435 |
| ASHGA5P039001 | noncoding | ENST00000383461 | H1FX-AS1 | -2.1573321 | 0.00000118 |
| ASHGA5P050753 | noncoding | ENST00000416200 | AC017096.1 | -2.1580346 | 0.00000344 |
| ASHGA5P029965 | noncoding | TCONS_00007945 | XLOC_003411 | -2.1583688 | 0.000127 |
| ASHGA5P022000 | noncoding | ENST00000546767 | RP11-983P16.4 | -2.158522 | 0.00689 |
| ASHGA5P042445 | noncoding | NR_024478 | LOC100132354 | -2.1605153 | 0.00472 |
| ASHGA5P049204 | noncoding | ENST00000563487 | RP11-80F22.2 | -2.1636484 | 0.000000176 |
| ASHGA5P047726 | noncoding | ENST00000445647 | RP11-328K15.1 | -2.1640514 | 0.0000229 |
| ASHGA5P032415 | noncoding | TCONS_00029899 | XLOC_014351 | -2.1671698 | 0.000237 |
| ASHGA5P023785 | noncoding | HMlincRNA420- | HMlincRNA420 | -2.1673557 | 0.00000102 |
| ASHGA5P038943 | noncoding | ENST00000496067 | RP11-180K7.1 | -2.1680255 | 0.0000114 |
| ASHGA5P058284 | noncoding | TCONS_00029180 | XLOC_014119 | -2.1721645 | 1.14E-09 |
| ASHGA5P015650 | noncoding | ENST00000422212 | RP13-492C18.2 | -2.1753934 | 0.00000104 |
| ASHGA5P052206 | noncoding | uc003jgw.2 | LOC643401 | -2.1791923 | 0.00000959 |
| ASHGA5P022294 | noncoding | ENST00000554029 | CTD-2058B24.3 | -2.1832905 | 0.00761 |
| ASHGA5P056698 | noncoding | TCONS_00007943 | XLOC_003411 | -2.1849161 | 0.000000211 |
| ASHGA5P056886 | noncoding | TCONS_00010247 | XLOC_004709 | -2.1851584 | 0.00000405 |
| ASHGA5P020389 | noncoding | ENST00000512650 | CTC-564N23.2 | -2.1880577 | 0.0000202 |
| ASHGA5P046650 | noncoding | TCONS_00017159 | XLOC_007967 | -2.1965177 | 0.00000105 |
| ASHGA5P030476 | noncoding | ENST00000434098 | RP11-85G21.2 | -2.1971129 | 0.000121 |
| ASHGA5P044271 | noncoding | TCONS_00001315 | XLOC_000595 | -2.1991463 | 0.0000202 |
| ASHGA5P014800 | noncoding | ENST00000413472 | RP1-163G9.1 | -2.199498 | 0.0000125 |
| ASHGA5P047416 | noncoding | ENST00000437213 | RP11-512N4.2 | -2.1995871 | 0.000342 |
| ASHGA5P040125 | noncoding | ENST00000506420 | RP11-793B23.1 | -2.2008638 | 0.000000121 |
| ASHGA5P028670 | noncoding | ENST00000412809 | MYO16-AS2 | -2.200966 | 0.0000128 |
| ASHGA5P035951 | noncoding | TCONS_00026984 | XLOC_013036 | -2.2026764 | 0.000000405 |
| ASHGA5P042697 | noncoding | TCONS_00019425 | XLOC_009243 | -2.2036312 | 5.92E-09 |
| ASHGA5P045352 | noncoding | TCONS_00000842 | XLOC_000057 | -2.2037303 | 0.0000123 |
| ASHGA5P020758 | noncoding | NR_024281 | LINC00599 | -2.2060752 | 0.00213 |
| ASHGA5P020062 | noncoding | ENST00000507566 | CTD-2024P10.1 | -2.2063708 | 0.00000113 |
| ASHGA5P041084 | noncoding | ENST00000508923 | CTD-2089N3.3 | -2.2069238 | 0.000000767 |
| ASHGA5P030815 | noncoding | ENST00000558838 | RP11-299G20.2 | -2.2085839 | 1.13E-08 |
| ASHGA5P046418 | noncoding | ENST00000420865 | RP11-309M23.1 | -2.21064 | 0.0411 |
| ASHGA5P047376 | noncoding | ENST00000451438 | RP11-168L22.2 | -2.2111582 | 2.72E-09 |
| ASHGA5P019704 | noncoding | ENST00000501050 | AC006445.8 | -2.2113782 | 1.63E-08 |
| ASHGA5P023692 | noncoding | HMlincRNA1534+ | HMlincRNA1534 | -2.2139327 | 0.000028 |
| ASHGA5P049886 | noncoding | TCONS_00002545 | XLOC_001134 | -2.2145501 | 0.000000158 |
| ASHGA5P031543 | noncoding | ENST00000565359 | RP11-552C15.1 | -2.2154789 | 0.0000893 |
| ASHGA5P044424 | noncoding | ENST00000569849 | KB-1000E4.2 | -2.2157174 | 1.19E-09 |
| ASHGA5P030358 | noncoding | ENST00000569908 | PWRN1 | -2.2159561 | 0.00014 |
| ASHGA5P053758 | noncoding | ENST00000422343 | RP11-180I4.2 | -2.2171616 | 0.000000476 |
| ASHGA5P035194 | noncoding | ENST00000428188 | AC007271.3 | -2.2173431 | 0.0000782 |
| ASHGA5P016385 | noncoding | ENST00000429949 | RP11-194G10.1 | -2.2181258 | 0.000514 |
| ASHGA5P040830 | noncoding | ENST00000509655 | CTB-109A12.1 | -2.2206692 | 0.0000286 |
| ASHGA5P020377 | noncoding | ENST00000512536 | LINC00616 | -2.2207856 | 0.000011 |
| ASHGA5P017524 | noncoding | ENST00000442712 | RP11-439E19.7 | -2.2207939 | 8.15E-09 |
| ASHGA5P022878 | noncoding | uc003luf.3 | BC034636 | -2.2211968 | 0.00000231 |
| ASHGA5P056918 | noncoding | TCONS_00010540 | XLOC_005067 | -2.2214477 | 0.000107 |
| ASHGA5P038282 | noncoding | NR_033384 | PDHB | -2.2226801 | 6.26E-11 |
| ASHGA5P042713 | noncoding | ENST00000434562 | RP1-249F5.3 | -2.2232383 | 9.86E-08 |
| ASHGA5P035287 | noncoding | TCONS_00004409 | XLOC_002283 | -2.2251237 | 2.62E-09 |
| ASHGA5P026075 | noncoding | ENST00000529417 | RP11-469N6.3 | -2.2253225 | 0.00000968 |
| ASHGA5P056348 | noncoding | TCONS_00003616 | XLOC_001406 | -2.2260103 | 0.000000314 |
| ASHGA5P052959 | noncoding | ENST00000442665 | ZNF815P | -2.22612 | 0.000000178 |
| ASHGA5P045251 | noncoding | ENST00000448485 | RP11-118H15.1 | -2.2262267 | 0.00000287 |
| ASHGA5P026803 | noncoding | ENST00000539222 | AP000797.4 | -2.2272311 | 7.18E-08 |
| ASHGA5P014861 | noncoding | ENST00000414107 | RP11-446F3.2 | -2.2297123 | 0.00000528 |
| ASHGA5P039856 | noncoding | ENST00000506514 | RP13-497K6.1 | -2.2333836 | 2.86E-08 |
| ASHGA5P047236 | noncoding | NR_036502 | LOC439949 | -2.2355598 | 0.00829 |
| ASHGA5P030807 | noncoding | ENST00000431060 | RP11-66B24.2 | -2.2381715 | 0.0000871 |
| ASHGA5P044771 | noncoding | ENST00000476186 | RP11-90P5.5 | -2.2387827 | 0.0000392 |
| ASHGA5P052119 | noncoding | ENST00000503478 | RP11-563E2.2 | -2.2425745 | 0.000111 |
| ASHGA5P036240 | noncoding | TCONS_00026870 | XLOC_012922 | -2.2469414 | 0.0000151 |
| ASHGA5P037073 | noncoding | ENST00000439786 | RP4-781B1.2 | -2.2492334 | 1.06E-09 |
| ASHGA5P047531 | noncoding | ENST00000432742 | RP11-90J7.3 | -2.2493216 | 0.00000001 |
| ASHGA5P020252 | noncoding | ENST00000510407 | RP11-39K24.10 | -2.2504905 | 1.57E-08 |
| ASHGA5P035579 | noncoding | TCONS_00015978 | XLOC_007352 | -2.2507278 | 2.09E-12 |
| ASHGA5P026240 | noncoding | ENST00000429099 | RP11-117D22.1 | -2.256189 | 0.0000126 |
| ASHGA5P036989 | noncoding | ENST00000432834 | RP5-860P4.2 | -2.2568193 | 0.00000747 |
| ASHGA5P057306 | noncoding | TCONS_00016551 | XLOC_007314 | -2.2605474 | 0.000334 |
| ASHGA5P027021 | noncoding | ENST00000430748 | RP5-837I24.4 | -2.2611044 | 0.0000065 |
| ASHGA5P030357 | noncoding | ENST00000565512 | PWRN1 | -2.266668 | 0.00143 |
| ASHGA5P039478 | noncoding | TCONS_00022645 | XLOC_010949 | -2.2711511 | 0.00000671 |
| ASHGA5P022134 | noncoding | ENST00000550042 | RP1-34H18.1 | -2.2715016 | 0.000000058 |
| ASHGA5P049981 | noncoding | ENST00000583267 | RP11-799B12.4 | -2.2722941 | 4.94E-09 |
| ASHGA5P042731 | noncoding | ENST00000568025 | RP1-168L15.5 | -2.2731667 | 0.000101 |
| ASHGA5P046511 | noncoding | TCONS_00001441 | XLOC_000757 | -2.2741676 | 2.32E-09 |
| ASHGA5P028800 | noncoding | ENST00000426509 | RP11-442J21.1 | -2.2769724 | 0.000000226 |
| ASHGA5P058273 | noncoding | TCONS_00029062 | XLOC_013983 | -2.2820793 | 0.00000121 |
| ASHGA5P040166 | noncoding | ENST00000504874 | RP11-503L19.1 | -2.2837157 | 0.00162 |
| ASHGA5P019155 | noncoding | NR_047553 | UBE2V1 | -2.2861852 | 0.0000473 |
| ASHGA5P038195 | noncoding | ENST00000419531 | RP4-794H19.2 | -2.2868213 | 0.0000729 |
| ASHGA5P032249 | noncoding | ENST00000573133 | RP11-28B23.1 | -2.2912607 | 0.0000115 |
| ASHGA5P023025 | noncoding | ENST00000568019 | PWRN1 | -2.2921544 | 0.000577 |
| ASHGA5P038484 | noncoding | ENST00000470739 | RP11-78O22.1 | -2.2956136 | 0.0000144 |
| ASHGA5P036414 | noncoding | NR_047698 | VWC2L-IT1 | -2.295927 | 0.000000864 |
| ASHGA5P054602 | noncoding | uc001ldf.3 | EMX2OS | -2.2967394 | 1.29E-10 |
| ASHGA5P030351 | noncoding | ENST00000558417 | RP11-566K19.5 | -2.2973798 | 6.06E-08 |
| ASHGA5P045296 | noncoding | ENST00000448475 | RP11-403N16.2 | -2.2987595 | 2.16E-11 |
| ASHGA5P032641 | noncoding | ENST00000580776 | CTD-2532D12.5 | -2.3021551 | 0.0000254 |
| ASHGA5P031419 | noncoding | ENST00000563515 | RP11-356C4.5 | -2.3043913 | 1.06E-09 |
| ASHGA5P028487 | noncoding | ENST00000568811 | RP11-307O13.1 | -2.304891 | 0.000654 |
| ASHGA5P031509 | noncoding | TCONS_00006632 | XLOC_003234 | -2.3139343 | 1.28E-08 |
| ASHGA5P035016 | noncoding | ENST00000444077 | AC017006.2 | -2.3181367 | 0.000226 |
| ASHGA5P042496 | noncoding | ENST00000445310 | KCNQ5-IT1 | -2.3183602 | 0.00167 |
| ASHGA5P037102 | noncoding | ENST00000425497 | RP11-290F20.3 | -2.3218716 | 7.69E-13 |
| ASHGA5P052812 | noncoding | NR_033557 | LOC100422737 | -2.3225257 | 1.58E-08 |
| ASHGA5P058226 | noncoding | TCONS_00028323 | XLOC_013664 | -2.3240318 | 0.00000323 |
| ASHGA5P033655 | noncoding | ENST00000578740 | RP11-126K15.1 | -2.3300203 | 0.000126 |
| ASHGA5P017582 | noncoding | ENST00000443364 | RP11-48O20.4 | -2.3316654 | 5.09E-11 |
| ASHGA5P010034 | noncoding | NR_033664 | NPSR1-AS1 | -2.3319858 | 0.000433 |
| ASHGA5P038868 | noncoding | uc003dnf.1 | AX747367 | -2.3323082 | 0.0000462 |
| ASHGA5P016059 | noncoding | ENST00000426329 | CARM1P1 | -2.3328075 | 0.00000837 |
| ASHGA5P035319 | noncoding | ENST00000419362 | AJ239322.1 | -2.3347013 | 0.00000131 |
| ASHGA5P037120 | noncoding | ENST00000434176 | RP5-843L14.1 | -2.3357418 | 0.00000059 |
| ASHGA5P036884 | noncoding | NR_003245 | HAR1B | -2.3378601 | 0.000128 |
| ASHGA5P018819 | noncoding | ENST00000457975 | RP11-168O22.1 | -2.3381692 | 0.0000172 |
| ASHGA5P045707 | noncoding | NR_024006 | FP588 | -2.3383434 | 0.0000136 |
| ASHGA5P042639 | noncoding | NR_038217 | LOC154092 | -2.3406213 | 0.0000185 |
| ASHGA5P031686 | noncoding | ENST00000576745 | RP11-388M20.9 | -2.3408698 | 0.0000198 |
| ASHGA5P026792 | noncoding | ENST00000425754 | RP11-181B18.1 | -2.3493336 | 0.00000621 |
| ASHGA5P044818 | noncoding | ENST00000521930 | RP11-400K9.3 | -2.353883 | 0.00000395 |
| ASHGA5P037100 | noncoding | ENST00000411453 | RP11-112L6.4 | -2.3541349 | 0.00000728 |
| ASHGA5P037376 | noncoding | TCONS_00024467 | XLOC_011789 | -2.3578885 | 0.0000181 |
| ASHGA5P022671 | noncoding | ENST00000560655 | RP11-279F6.3 | -2.3593472 | 0.0000268 |
| ASHGA5P019246 | noncoding | ENST00000475196 | PLCH1-AS1 | -2.3606094 | 0.00000462 |
| ASHGA5P015574 | noncoding | ENST00000451257 | KB-1183D5.11 | -2.3638529 | 0.0000293 |
| ASHGA5P015768 | noncoding | ENST00000423332 | CYP4Z2P | -2.3641092 | 0.0000186 |
| ASHGA5P038246 | noncoding | ENST00000421735 | U73167.7 | -2.3642234 | 0.000139 |
| ASHGA5P033373 | noncoding | TCONS_00028471 | XLOC_013844 | -2.3655272 | 0.000102 |
| ASHGA5P032259 | noncoding | ENST00000579897 | CTB-187M2.2 | -2.3655637 | 0.00000212 |
| ASHGA5P056798 | noncoding | TCONS_00008738 | XLOC_003422 | -2.3662917 | 0.0000115 |
| ASHGA5P020671 | noncoding | ENST00000518232 | CTD-3064M3.1 | -2.3667746 | 0.0000925 |
| ASHGA5P034876 | noncoding | ENST00000569008 | RP11-434B12.1 | -2.3685375 | 4.38E-08 |
| ASHGA5P022403 | noncoding | ENST00000556168 | RP11-1152H15.1 | -2.3694925 | 0.000039 |
| ASHGA5P037784 | noncoding | uc002zls.1 | TPTEP1 | -2.3718062 | 0.00532 |
| ASHGA5P042646 | noncoding | ENST00000536296 | LINC00271 | -2.3729187 | 0.00000229 |
| ASHGA5P015420 | noncoding | ENST00000420130 | AC010745.4 | -2.3773351 | 0.000192 |
| ASHGA5P035894 | noncoding | ENST00000433396 | AC007392.3 | -2.3809635 | 0.0000192 |
| ASHGA5P039807 | noncoding | NR_026892 | AFAP1-AS1 | -2.3839636 | 0.000000378 |
| ASHGA5P043225 | noncoding | ENST00000434983 | RP11-309H21.2 | -2.3864138 | 9.68E-08 |
| ASHGA5P031275 | noncoding | ENST00000572770 | RP11-510M2.4 | -2.3878906 | 0.0000666 |
| ASHGA5P017009 | noncoding | uc010fjy.3 | LOC151009 | -2.387947 | 0.00000127 |
| ASHGA5P027157 | noncoding | ENST00000537327 | RP11-977P2.1 | -2.3931923 | 0.000000156 |
| ASHGA5P040060 | noncoding | NR_029373 | LEF1-AS1 | -2.3990987 | 0.00000418 |
| ASHGA5P031160 | noncoding | ENST00000567728 | RP11-327F22.2 | -2.3994159 | 6.98E-08 |
| ASHGA5P055302 | noncoding | ENST00000539178 | RP11-711K1.7 | -2.4031846 | 3.21E-09 |
| ASHGA5P031684 | noncoding | uc002ebp.1 | TRIM72 | -2.4039599 | 0.00000012 |
| ASHGA5P029854 | noncoding | ENST00000500487 | RP11-32B5.7 | -2.4055514 | 0.000443 |
| ASHGA5P030057 | noncoding | TCONS_00007487 | XLOC_003471 | -2.4079441 | 3.82E-09 |
| ASHGA5P057317 | noncoding | TCONS_00016047 | XLOC_007440 | -2.4106646 | 0.0000039 |
| ASHGA5P036010 | noncoding | TCONS_00027064 | XLOC_013103 | -2.4112619 | 2.28E-09 |
| ASHGA5P022198 | noncoding | NR_038242 | VEZT | -2.4124408 | 0.0000268 |
| ASHGA5P045828 | noncoding | ENST00000411450 | RP11-526D8.7 | -2.4155272 | 0.00000144 |
| ASHGA5P056799 | noncoding | TCONS_00008739 | XLOC_003422 | -2.4170609 | 0.0000237 |
| ASHGA5P053545 | noncoding | ENST00000574086 | RP11-760H22.2 | -2.4171152 | 0.0000102 |
| ASHGA5P051192 | noncoding | ENST00000540720 | DGCR5 | -2.417347 | 0.0000291 |
| ASHGA5P015753 | noncoding | NR_027088 | LOC284661 | -2.4197072 | 0.00557 |
| ASHGA5P056664 | noncoding | TCONS_00007011 | XLOC_002884 | -2.4234246 | 4.15E-08 |
| ASHGA5P035989 | noncoding | ENST00000421951 | ANKRD36BP2 | -2.4238983 | 0.00000164 |
| ASHGA5P055426 | noncoding | ENST00000549388 | RP11-983P16.4 | -2.4266259 | 0.000041 |
| ASHGA5P037975 | noncoding | ENST00000425802 | RP11-91A18.4 | -2.4295995 | 0.000204 |
| ASHGA5P016945 | noncoding | ENST00000435921 | ABC12-49244600F4.3 | -2.432671 | 0.00000317 |
| ASHGA5P019200 | noncoding | ENST00000472596 | RP11-496B10.3 | -2.4334247 | 0.000000194 |
| ASHGA5P022185 | noncoding | ENST00000551726 | RP11-81H3.2 | -2.4341673 | 0.000000143 |
| ASHGA5P057707 | noncoding | TCONS_00020947 | XLOC_010216 | -2.4352439 | 0.000000109 |
| ASHGA5P021913 | noncoding | ENST00000544306 | AC062028.1 | -2.4357696 | 3.63E-08 |
| ASHGA5P049031 | noncoding | ENST00000570949 | RP11-473M20.7 | -2.4383601 | 3.52E-09 |
| ASHGA5P052503 | noncoding | NR_026682 | LOC100268168 | -2.4400332 | 0.00000165 |
| ASHGA5P042112 | noncoding | NR_034115 | STXBP5-AS1 | -2.4425494 | 3.38E-08 |
| ASHGA5P056448 | noncoding | TCONS_00004332 | XLOC_002206 | -2.4431863 | 0.0000326 |
| ASHGA5P029889 | noncoding | ENST00000560740 | RP11-680F8.4 | -2.4442606 | 0.00000072 |
| ASHGA5P036311 | noncoding | ENST00000447478 | RP4-534N18.2 | -2.4460366 | 2.15E-08 |
| ASHGA5P041667 | noncoding | ENST00000472178 | LINC00518 | -2.446108 | 1.44E-08 |
| ASHGA5P036994 | noncoding | ENST00000376445 | RP13-401N8.1 | -2.4484211 | 0.0000411 |
| ASHGA5P036133 | noncoding | ENST00000454503 | AC010976.2 | -2.4494682 | 0.00000568 |
| ASHGA5P006332 | noncoding | ENST00000540211 | MIR7-3HG | -2.4507165 | 0.00207 |
| ASHGA5P042113 | noncoding | ENST00000367477 | RP11-497D6.4 | -2.4510224 | 0.000000268 |
| ASHGA5P035498 | noncoding | ENST00000436642 | RP5-930J4.2 | -2.4568837 | 0.000000111 |
| ASHGA5P031392 | noncoding | ENST00000566351 | RP11-863P13.4 | -2.458894 | 0.000376 |
| ASHGA5P042408 | noncoding | TCONS_00020667 | XLOC_009971 | -2.4635257 | 0.000000256 |
| ASHGA5P038013 | noncoding | NR_024355 | LINC00634 | -2.4640689 | 0.00000121 |
| ASHGA5P040783 | noncoding | ENST00000434047 | RP11-277L2.2 | -2.4653697 | 1.24E-14 |
| ASHGA5P046086 | noncoding | TCONS_00001635 | XLOC_000972 | -2.4669671 | 0.000000915 |
| ASHGA5P015050 | noncoding | ENST00000416028 | RP11-119F7.4 | -2.4701891 | 0.00000316 |
| ASHGA5P051211 | noncoding | ENST00000414022 | TMEM191A | -2.4706401 | 0.000000341 |
| ASHGA5P000166 | noncoding | chr1:38888750-38900850+ | chr1:38888750-38900850 | -2.4716494 | 0.0000649 |
| ASHGA5P023568 | noncoding | ENST00000584346 | RP11-21G15.1 | -2.4730888 | 0.0000428 |
| ASHGA5P028435 | noncoding | uc010tdg.1 | AK054988 | -2.4732362 | 0.00296 |
| ASHGA5P033495 | noncoding | ENST00000581798 | CTD-3096M3.2 | -2.473458 | 1.22E-11 |
| ASHGA5P034930 | noncoding | TCONS_00003589 | XLOC_001373 | -2.4757828 | 0.000000349 |
| ASHGA5P037146 | noncoding | ENST00000448923 | RP11-96L14.7 | -2.4803172 | 0.0000121 |
| ASHGA5P033289 | noncoding | TCONS_00028426 | XLOC_013778 | -2.4837824 | 0.0000987 |
| ASHGA5P041824 | noncoding | ENST00000414505 | RP11-121P10.1 | -2.4838313 | 0.00000391 |
| ASHGA5P035296 | noncoding | NR_036537 | LOC100131320 | -2.4838552 | 0.00005 |
| ASHGA5P033857 | noncoding | NR_040075 | LOC100287225 | -2.4844839 | 0.0000663 |
| ASHGA5P014599 | noncoding | ENST00000411710 | AC010095.5 | -2.4854697 | 0.0000163 |
| ASHGA5P038192 | noncoding | ENST00000438017 | RP4-613B23.1 | -2.4866014 | 0.00000186 |
| ASHGA5P034949 | noncoding | NR_038319 | LOC100505716 | -2.486912 | 0.00000803 |
| ASHGA5P014610 | noncoding | ENST00000411795 | BX248398.1 | -2.4871118 | 0.0000497 |
| ASHGA5P045719 | noncoding | ENST00000449585 | RP11-631M21.1 | -2.4897337 | 0.0000436 |
| ASHGA5P048190 | noncoding | ENST00000543817 | RP11-21A7A.3 | -2.4901851 | 0.000328 |
| ASHGA5P029843 | noncoding | ENST00000426501 | HERC2P3 | -2.4905901 | 0.0069 |
| ASHGA5P029844 | noncoding | ENST00000428453 | HERC2P3 | -2.4938735 | 0.00389 |
| ASHGA5P039462 | noncoding | uc003hkh.3 | DQ576800 | -2.4944748 | 1.85E-09 |
| ASHGA5P058802 | noncoding | uc.85+ | uc.85 | -2.495276 | 5.49E-08 |
| ASHGA5P050379 | noncoding | ENST00000424351 | AC017076.4 | -2.4956913 | 1.36E-10 |
| ASHGA5P039485 | noncoding | ENST00000564854 | RP13-514E23.1 | -2.5000293 | 0.00000196 |
| ASHGA5P047302 | noncoding | ENST00000434458 | RP13-16H11.1 | -2.5048915 | 0.00000024 |
| ASHGA5P028437 | noncoding | ENST00000420140 | LINC00357 | -2.5203489 | 0.000435 |
| ASHGA5P039188 | noncoding | ENST00000491676 | KLHL6-AS1 | -2.5246775 | 0.00000305 |
| ASHGA5P033978 | noncoding | NR_027064 | PLAC2 | -2.5266289 | 0.000016 |
| ASHGA5P042541 | noncoding | ENST00000424678 | RP11-524K14.1 | -2.528544 | 0.00000476 |
| ASHGA5P029712 | noncoding | ENST00000563574 | CTD-2547L24.4 | -2.5289452 | 0.00000458 |
| ASHGA5P016449 | noncoding | ENST00000430518 | AC018647.3 | -2.5297523 | 0.00014 |
| ASHGA5P033343 | noncoding | ENST00000569655 | RP11-143K11.1 | -2.5323307 | 0.0013 |
| ASHGA5P056903 | noncoding | TCONS_00010377 | XLOC_004885 | -2.5323846 | 0.0000279 |
| ASHGA5P037672 | noncoding | ENST00000450365 | LL22NC03-13G6.2 | -2.5333558 | 0.0000266 |
| ASHGA5P029028 | noncoding | ENST00000554526 | RP11-388E23.2 | -2.5348073 | 8.61E-08 |
| ASHGA5P000545 | noncoding | chr9:110109500-110126925- | chr9:110109500-110126925 | -2.5357472 | 1.38E-08 |
| ASHGA5P015646 | noncoding | ENST00000422194 | RP11-402P6.9 | -2.5369038 | 0.00000716 |
| ASHGA5P047173 | noncoding | ENST00000414106 | RP11-140A10.3 | -2.5460415 | 0.00000959 |
| ASHGA5P049573 | noncoding | ENST00000579693 | MTND1P15 | -2.5476908 | 4.17E-08 |
| ASHGA5P048684 | noncoding | ENST00000561094 | RP11-605F22.2 | -2.5595456 | 1.71E-08 |
| ASHGA5P035794 | noncoding | NR_027098 | LINC00486 | -2.5595783 | 0.000000325 |
| ASHGA5P048699 | noncoding | ENST00000559977 | RP11-707P17.2 | -2.5596384 | 0.00315 |
| ASHGA5P029726 | noncoding | TCONS_00008088 | XLOC_003550 | -2.5619292 | 0.000000163 |
| ASHGA5P032490 | noncoding | TCONS_00029734 | XLOC_014409 | -2.5621797 | 9.77E-09 |
| ASHGA5P036747 | noncoding | TCONS_00026539 | XLOC_012861 | -2.5621824 | 7.09E-11 |
| ASHGA5P026945 | noncoding | uc021qsp.1 | AK125040 | -2.5677262 | 0.000398 |
| ASHGA5P029862 | noncoding | NR_040057 | LOC283683 | -2.5685667 | 0.0000978 |
| ASHGA5P057605 | noncoding | TCONS_00019576 | XLOC_009375 | -2.5706143 | 5.92E-08 |
| ASHGA5P055809 | noncoding | ENST00000424524 | ATXN8OS | -2.5714344 | 1.33E-08 |
| ASHGA5P056204 | noncoding | TCONS_00000998 | XLOC_000263 | -2.5723731 | 2.91E-09 |
| ASHGA5P037482 | noncoding | ENST00000416842 | AP001042.1 | -2.5753657 | 5.18E-08 |
| ASHGA5P028743 | noncoding | TCONS_00011011 | XLOC_004945 | -2.5762272 | 0.0000394 |
| ASHGA5P038245 | noncoding | NR_045388 | LUST | -2.5762427 | 0.00000444 |
| ASHGA5P027759 | noncoding | ENST00000538202 | RP11-429A20.4 | -2.5786808 | 1.07E-08 |
| ASHGA5P021348 | noncoding | ENST00000530576 | RP11-266A24.1 | -2.5799077 | 0.00000345 |
| ASHGA5P029834 | noncoding | ENST00000546968 | RP11-44N21.1 | -2.5803334 | 0.00000053 |
| ASHGA5P021555 | noncoding | ENST00000534886 | RP11-364C11.3 | -2.5821442 | 0.00261 |
| ASHGA5P057514 | noncoding | TCONS_00018881 | XLOC_008729 | -2.5920732 | 1.21E-09 |
| ASHGA5P037838 | noncoding | TCONS_00024733 | XLOC_012014 | -2.5940409 | 0.000000388 |
| ASHGA5P057308 | noncoding | TCONS_00015980 | XLOC_007354 | -2.5960114 | 2.74E-08 |
| ASHGA5P036322 | noncoding | ENST00000438505 | AC018799.1 | -2.5965391 | 6.54E-09 |
| ASHGA5P016650 | noncoding | ENST00000432535 | RP11-456A18.1 | -2.5984883 | 0.000165 |
| ASHGA5P038151 | noncoding | ENST00000565519 | RP11-384L8.1 | -2.6056502 | 0.00000425 |
| ASHGA5P031078 | noncoding | ENST00000457405 | RP1-10C16.1 | -2.6141728 | 0.0000111 |
| ASHGA5P021290 | noncoding | ENST00000532352 | FOLH1B | -2.6181865 | 0.000116 |
| ASHGA5P016560 | noncoding | ENST00000431695 | RP11-245J24.1 | -2.6191692 | 2.21E-08 |
| ASHGA5P045753 | noncoding | ENST00000414223 | RP11-561O23.5 | -2.6271718 | 0.00013 |
| ASHGA5P014784 | noncoding | ENST00000413332 | RP13-15E13.1 | -2.6272118 | 0.00000217 |
| ASHGA5P047532 | noncoding | uc001jzw.1 | AK126491 | -2.6313378 | 0.000111 |
| ASHGA5P039042 | noncoding | ENST00000411798 | RP11-286B14.1 | -2.6365854 | 0.0000392 |
| ASHGA5P021339 | noncoding | ENST00000530344 | CTD-2003C8.1 | -2.6413951 | 2.86E-08 |
| ASHGA5P040130 | noncoding | ENST00000515860 | LINC00500 | -2.643531 | 8.18E-11 |
| ASHGA5P021669 | noncoding | ENST00000537720 | RP13-895J2.7 | -2.6457804 | 8.79E-10 |
| ASHGA5P058477 | noncoding | uc003zpr.3 | CR627240 | -2.6477185 | 0.0000697 |
| ASHGA5P035512 | noncoding | NR_037886 | LOC100507140 | -2.6488585 | 0.0000414 |
| ASHGA5P035707 | noncoding | ENST00000478468 | RP11-521D12.1 | -2.6555691 | 2.22E-09 |
| ASHGA5P033234 | noncoding | ENST00000441895 | RP11-1018N14.2 | -2.6568133 | 0.000000493 |
| ASHGA5P057437 | noncoding | TCONS_00017336 | XLOC_008183 | -2.6583593 | 0.000117 |
| ASHGA5P018794 | noncoding | ENST00000457716 | RP11-402P6.7 | -2.6603234 | 0.000343 |
| ASHGA5P040214 | noncoding | ENST00000508189 | RP11-234O6.2 | -2.6607809 | 0.000000815 |
| ASHGA5P056436 | noncoding | TCONS_00004267 | XLOC_002109 | -2.6641717 | 6.73E-08 |
| ASHGA5P031860 | noncoding | TCONS_00006572 | XLOC_003199 | -2.6681468 | 6.42E-08 |
| ASHGA5P031350 | noncoding | ENST00000563342 | RP11-298D21.2 | -2.6712204 | 0.000248 |
| ASHGA5P022843 | noncoding | NR_026799 | SH3GL3 | -2.6717738 | 0.000105 |
| ASHGA5P029969 | noncoding | TCONS_00008729 | XLOC_003411 | -2.6805615 | 0.000584 |
| ASHGA5P031404 | noncoding | ENST00000537498 | CTD-2555A7.2 | -2.6826882 | 0.00488 |
| ASHGA5P047778 | noncoding | ENST00000445868 | RP11-462G8.2 | -2.6828833 | 8.15E-10 |
| ASHGA5P045336 | noncoding | ENST00000450451 | RP11-193H5.1 | -2.6879874 | 0.00000242 |
| ASHGA5P044110 | noncoding | uc022ars.1 | AK091593 | -2.6923538 | 0.000166 |
| ASHGA5P040556 | noncoding | uc011cub.2 | LINC00461 | -2.6948878 | 6.17E-10 |
| ASHGA5P044499 | noncoding | TCONS_00018433 | XLOC_008729 | -2.6975053 | 2.71E-08 |
| ASHGA5P029600 | noncoding | ENST00000568344 | RP11-355I22.7 | -2.6989054 | 0.0000634 |
| ASHGA5P036323 | noncoding | uc002utf.1 | BC038548 | -2.7010425 | 4.86E-10 |
| ASHGA5P037385 | noncoding | ENST00000470891 | AF130249.5 | -2.7024803 | 0.0000013 |
| ASHGA5P040709 | noncoding | NR_024418 | LOC389332 | -2.7112901 | 0.00364 |
| ASHGA5P045849 | noncoding | ENST00000417149 | RP11-298E9.6 | -2.7139867 | 1.21E-09 |
| ASHGA5P009382 | noncoding | ENST00000354752 | ANKRD18CP | -2.7196228 | 7.19E-09 |
| ASHGA5P023324 | noncoding | NR_038418 | BZRAP1-AS1 | -2.7199272 | 0.00000121 |
| ASHGA5P038276 | noncoding | NR_024615 | ERC2-IT1 | -2.720401 | 0.0000415 |
| ASHGA5P058380 | noncoding | uc002jtz.3 | BC040189 | -2.7220107 | 0.0000702 |
| ASHGA5P047583 | noncoding | uc001kgy.3 | BC037970 | -2.7272639 | 4.07E-08 |
| ASHGA5P046473 | noncoding | ENST00000424251 | RP1-146A15.1 | -2.7305503 | 0.0133 |
| ASHGA5P016925 | noncoding | ENST00000435744 | ANKRD30BP2 | -2.731706 | 0.00000105 |
| ASHGA5P023132 | noncoding | NR_040011 | LOC100506388 | -2.7321541 | 0.000000122 |
| ASHGA5P048224 | noncoding | ENST00000533287 | RP11-867G23.2 | -2.737193 | 1.49E-09 |
| ASHGA5P037834 | noncoding | ENST00000422216 | RP1-18D14.7 | -2.7507002 | 2.81E-09 |
| ASHGA5P044075 | noncoding | NR_026817 | LOC148696 | -2.7527737 | 1.21E-08 |
| ASHGA5P034182 | noncoding | ENST00000532488 | CTD-2525J15.2 | -2.7628602 | 0.00000129 |
| ASHGA5P045579 | noncoding | TCONS_00001799 | XLOC_001188 | -2.764217 | 0.000000361 |
| ASHGA5P030684 | noncoding | ENST00000564933 | RP11-160C18.2 | -2.7646107 | 6.57E-10 |
| ASHGA5P050090 | noncoding | ENST00000518557 | LINC00599 | -2.7745479 | 0.00988 |
| ASHGA5P040051 | noncoding | ENST00000500179 | AC004053.1 | -2.7792982 | 8.12E-09 |
| ASHGA5P055499 | noncoding | ENST00000548410 | RP11-571M6.8 | -2.7845185 | 0.000238 |
| ASHGA5P042106 | noncoding | NR_002768 | HYMAI | -2.7849125 | 0.000000698 |
| ASHGA5P031772 | noncoding | ENST00000569713 | RP11-142G1.2 | -2.785248 | 0.000000335 |
| ASHGA5P044665 | noncoding | ENST00000527110 | RP11-3G21.1 | -2.7875939 | 8.89E-12 |
| ASHGA5P031395 | noncoding | ENST00000568587 | RP11-863P13.2 | -2.8072958 | 1.17E-10 |
| ASHGA5P055181 | noncoding | ENST00000453118 | RP1-163G9.1 | -2.8083713 | 0.000022 |
| ASHGA5P023928 | noncoding | HMlincRNA971+ | HMlincRNA971 | -2.8124753 | 0.0000296 |
| ASHGA5P021549 | noncoding | ENST00000534756 | RP11-64I17.1 | -2.8136331 | 0.00264 |
| ASHGA5P044876 | noncoding | ENST00000564832 | RP11-531A24.3 | -2.8168617 | 0.00554 |
| ASHGA5P016249 | noncoding | ENST00000428531 | RP11-261N11.8 | -2.8186982 | 0.000117 |
| ASHGA5P021381 | noncoding | ENST00000531315 | FOLH1B | -2.8195807 | 0.00000128 |
| ASHGA5P038995 | noncoding | uc001dqe.1 | AX746627 | -2.8205051 | 8.18E-10 |
| ASHGA5P039311 | noncoding | ENST00000511928 | AC141928.1 | -2.8231609 | 0.0000108 |
| ASHGA5P026714 | noncoding | ENST00000529567 | FOLH1B | -2.8301598 | 0.0000311 |
| ASHGA5P022818 | noncoding | NR_024399 | MGC23284 | -2.8391327 | 4.66E-10 |
| ASHGA5P016564 | noncoding | ENST00000431722 | RP5-1185I7.1 | -2.8437425 | 2.11E-11 |
| ASHGA5P045099 | noncoding | ENST00000435421 | RP11-143M1.3 | -2.8456723 | 0.000397 |
| ASHGA5P032530 | noncoding | TCONS_00029029 | XLOC_013950 | -2.8490002 | 4.22E-10 |
| ASHGA5P036836 | noncoding | uc002xuq.1 | AK055386 | -2.8536625 | 0.000029 |
| ASHGA5P030385 | noncoding | ENST00000567953 | AC144833.1 | -2.8539245 | 1.81E-10 |
| ASHGA5P051060 | noncoding | ENST00000370257 | RP11-261N11.8 | -2.8661055 | 0.000171 |
| ASHGA5P042515 | noncoding | ENST00000429444 | RP1-232L24.3 | -2.8719607 | 0.0000576 |
| ASHGA5P049595 | noncoding | ENST00000579187 | RP11-20B24.4 | -2.8762856 | 0.000141 |
| ASHGA5P039841 | noncoding | ENST00000573950 | RP11-783N5.1 | -2.8771653 | 0.0000204 |
| ASHGA5P028690 | noncoding | NR_028064 | LINC00552 | -2.8890647 | 4.86E-09 |
| ASHGA5P021356 | noncoding | ENST00000530792 | CTD-2562J17.4 | -2.8909178 | 3.87E-14 |
| ASHGA5P027846 | noncoding | ENST00000535528 | RP11-4N23.1 | -2.898759 | 1.36E-08 |
| ASHGA5P037621 | noncoding | ENST00000411511 | RP3-462D8.2 | -2.9115382 | 0.0000781 |
| ASHGA5P031039 | noncoding | ENST00000568895 | CTD-2515A14.1 | -2.9169987 | 6.52E-12 |
| ASHGA5P026679 | noncoding | ENST00000527440 | RP11-802F5.1 | -2.9211581 | 9.42E-09 |
| ASHGA5P033768 | noncoding | ENST00000578285 | CYP4F35P | -2.9236079 | 0.00000062 |
| ASHGA5P031957 | noncoding | ENST00000567109 | RP11-483P21.3 | -2.9274809 | 0.00000017 |
| ASHGA5P046437 | noncoding | ENST00000432442 | GS1-519E5.1 | -2.928099 | 2.7E-09 |
| ASHGA5P036393 | noncoding | TCONS_00026329 | XLOC_012676 | -2.9347982 | 0.000000466 |
| ASHGA5P038813 | noncoding | TCONS_00023287 | XLOC_011157 | -2.9391949 | 0.000184 |
| ASHGA5P029974 | noncoding | TCONS_00007953 | XLOC_003422 | -2.9403935 | 8.89E-11 |
| ASHGA5P041557 | noncoding | ENST00000507361 | RP11-267A15.1 | -2.9429498 | 0.000347 |
| ASHGA5P055538 | noncoding | uc001sxc.3 | LOC100507377 | -2.9466191 | 0.000000568 |
| ASHGA5P058484 | noncoding | uc004afh.2 | LOC440896 | -2.9512489 | 0.000108 |
| ASHGA5P025845 | noncoding | NR_038887 | FLJ41649 | -2.958927 | 0.000138 |
| ASHGA5P036283 | noncoding | ENST00000582038 | RP11-88L24.4 | -2.9603959 | 0.000334 |
| ASHGA5P053237 | noncoding | uc003uyp.4 | BC039094 | -2.9623325 | 0.000000906 |
| ASHGA5P018297 | noncoding | ENST00000451704 | AC015818.3 | -2.9836041 | 3.8E-13 |
| ASHGA5P057931 | noncoding | TCONS_00023869 | XLOC_011178 | -2.9857641 | 0.00000248 |
| ASHGA5P016997 | noncoding | ENST00000436430 | RP11-245J24.1 | -2.9893479 | 0.000000033 |
| ASHGA5P030689 | noncoding | TCONS_00006338 | XLOC_002953 | -2.9906034 | 0.00000105 |
| ASHGA5P026131 | noncoding | NR_024627 | KCNQ1DN | -2.9961621 | 0.00195 |
| ASHGA5P044662 | noncoding | ENST00000523495 | RP11-10C8.2 | -2.9985642 | 4.59E-10 |
| ASHGA5P028925 | noncoding | NR_034119 | LINC00460 | -3.0106966 | 0.00000401 |
| ASHGA5P043559 | noncoding | ENST00000430548 | AC004540.5 | -3.0113018 | 0.00000189 |
| ASHGA5P055070 | noncoding | ENST00000533785 | FOLH1B | -3.0216996 | 0.00000871 |
| ASHGA5P057996 | noncoding | TCONS_00024756 | XLOC_012040 | -3.0235405 | 0.000000275 |
| ASHGA5P044613 | noncoding | ENST00000577187 | ERICH1-AS1 | -3.0261848 | 0.0000657 |
| ASHGA5P028622 | noncoding | ENST00000450888 | LINC00438 | -3.026667 | 0.00000496 |
| ASHGA5P057932 | noncoding | TCONS_00023917 | XLOC_011215 | -3.031067 | 0.0000148 |
| ASHGA5P021193 | noncoding | ENST00000527345 | RP11-99C10.1 | -3.0513361 | 0.0000171 |
| ASHGA5P043527 | noncoding | ENST00000411542 | AC011288.2 | -3.0552497 | 0.000000599 |
| ASHGA5P028740 | noncoding | TCONS_00011012 | XLOC_004945 | -3.0571109 | 0.000000171 |
| ASHGA5P035193 | noncoding | TCONS_00004369 | XLOC_002251 | -3.0581869 | 1.09E-13 |
| ASHGA5P047203 | noncoding | NR_033387 | ADARB2-AS1 | -3.0590059 | 0.0000011 |
| ASHGA5P039857 | noncoding | ENST00000569621 | RP11-496D24.2 | -3.0750629 | 0.0000119 |
| ASHGA5P028108 | noncoding | ENST00000540024 | RP11-766N7.3 | -3.0814581 | 6.14E-11 |
| ASHGA5P015210 | noncoding | ENST00000417942 | XIST | -3.0821677 | 0.000569 |
| ASHGA5P043640 | noncoding | ENST00000441052 | AC017116.8 | -3.0842246 | 0.00000226 |
| ASHGA5P041326 | noncoding | ENST00000433406 | CTC-546K23.1 | -3.0891722 | 6.32E-09 |
| ASHGA5P027187 | noncoding | ENST00000545923 | RP11-313F23.4 | -3.1002321 | 1.29E-09 |
| ASHGA5P032172 | noncoding | ENST00000579367 | CTC-297N7.9 | -3.1097444 | 0.000000224 |
| ASHGA5P025783 | noncoding | NR_037635 | GJA9-MYCBP | -3.113487 | 0.000224 |
| ASHGA5P057884 | noncoding | TCONS_00023303 | XLOC_011172 | -3.118067 | 0.000000681 |
| ASHGA5P033756 | noncoding | NR_049896 | C18orf61 | -3.121406 | 7.06E-08 |
| ASHGA5P018710 | noncoding | ENST00000456683 | RP13-1039J1.3 | -3.1222484 | 0.00000671 |
| ASHGA5P020321 | noncoding | ENST00000511520 | RP11-655M19.1 | -3.1286874 | 2.34E-09 |
| ASHGA5P028109 | noncoding | NR_033988 | FLJ41278 | -3.1315269 | 3.42E-10 |
| ASHGA5P038836 | noncoding | TCONS_00023867 | XLOC_011177 | -3.1370511 | 0.0000018 |
| ASHGA5P041110 | noncoding | NR_024617 | PART1 | -3.1375953 | 0.000000116 |
| ASHGA5P056700 | noncoding | TCONS_00007947 | XLOC_003411 | -3.1382653 | 0.00000526 |
| ASHGA5P047509 | noncoding | ENST00000413901 | RP5-1180C18.1 | -3.141537 | 9.94E-08 |
| ASHGA5P021200 | noncoding | ENST00000534201 | RP11-707M1.1 | -3.1473583 | 0.0000308 |
| ASHGA5P045386 | noncoding | uc004bdv.3 | AL390170 | -3.1487552 | 0.0000503 |
| ASHGA5P016793 | noncoding | ENST00000434329 | RP11-446F17.3 | -3.1510152 | 8.98E-11 |
| ASHGA5P034955 | noncoding | ENST00000446073 | AC105398.3 | -3.1528554 | 0.000227 |
| ASHGA5P023155 | noncoding | ENST00000571590 | RP11-446F17.3 | -3.1561073 | 3.73E-10 |
| ASHGA5P028436 | noncoding | uc001upw.3 | TPTE2P1 | -3.1584301 | 0.00000735 |
| ASHGA5P023168 | noncoding | ENST00000572062 | AP005530.2 | -3.1686569 | 0.000105 |
| ASHGA5P039919 | noncoding | ENST00000562284 | RP11-731J8.2 | -3.1708704 | 0.00000224 |
| ASHGA5P029214 | noncoding | ENST00000554737 | RP11-109N23.4 | -3.1794932 | 1.55E-11 |
| ASHGA5P026908 | noncoding | uc001qeg.1 | AK095187 | -3.2021105 | 2.51E-10 |
| ASHGA5P025715 | noncoding | NR_027910 | CALB2 | -3.2127862 | 0.0000103 |
| ASHGA5P029775 | noncoding | ENST00000398460 | MEG3 | -3.2174846 | 0.000198 |
| ASHGA5P040313 | noncoding | ENST00000564741 | CTC-471C19.1 | -3.2282124 | 0.000000889 |
| ASHGA5P016537 | noncoding | ENST00000431442 | RP11-64P14.7 | -3.239295 | 5.24E-08 |
| ASHGA5P049805 | noncoding | ENST00000580942 | RP11-214C8.2 | -3.2427011 | 2.91E-10 |
| ASHGA5P015112 | noncoding | ENST00000419299 | LINC00320 | -3.2439612 | 0.0000489 |
| ASHGA5P041565 | noncoding | ENST00000512115 | RP11-375B1.3 | -3.2455179 | 0.0000259 |
| ASHGA5P026861 | noncoding | ENST00000526777 | RP11-716H6.2 | -3.2544611 | 0.000000464 |
| ASHGA5P029508 | noncoding | ENST00000553046 | RP11-317N8.3 | -3.2658482 | 0.000000464 |
| ASHGA5P016665 | noncoding | ENST00000432699 | RP11-464O2.2 | -3.2742259 | 0.000000448 |
| ASHGA5P051568 | noncoding | ENST00000482372 | RP11-147N17.1 | -3.5257371 | 0.0000789 |
| ASHGA5P027724 | noncoding | ENST00000539568 | RP11-283I3.2 | -3.5372473 | 0.00000143 |
| ASHGA5P044614 | noncoding | ENST00000524139 | ERICH1-AS1 | -3.5452168 | 0.000367 |
| ASHGA5P033458 | noncoding | ENST00000572608 | AP005530.2 | -3.556332 | 0.00000537 |
| ASHGA5P035041 | noncoding | ENST00000563570 | RP4-597A16.2 | -3.5795014 | 0.000000398 |
| ASHGA5P017761 | noncoding | ENST00000445452 | RP4-613B23.1 | -3.5881851 | 0.000000279 |
| ASHGA5P040311 | noncoding | uc003jdi.1 | AK094462 | -3.5927578 | 0.00000777 |
| ASHGA5P044860 | noncoding | uc003xvd.3 | LOC100130155 | -3.5962001 | 0.00118 |
| ASHGA5P058114 | noncoding | TCONS_00026612 | XLOC_012663 | -3.5995615 | 0.000000074 |
| ASHGA5P051075 | noncoding | ENST00000471407 | ANKRD30BP2 | -3.6020295 | 0.000000101 |
| ASHGA5P022951 | noncoding | ENST00000566268 | RP11-673C5.2 | -3.6051566 | 3.99E-08 |
| ASHGA5P018361 | noncoding | uc002yky.4 | LINC00320 | -3.6088601 | 0.0000541 |
| ASHGA5P027346 | noncoding | ENST00000553176 | RP11-977G19.5 | -3.6186423 | 0.000000498 |
| ASHGA5P036336 | noncoding | NR_026830 | SATB2-AS1 | -3.6233394 | 0.000000994 |
| ASHGA5P030497 | noncoding | ENST00000411560 | AC011330.12 | -3.6243543 | 0.000000942 |
| ASHGA5P041012 | noncoding | ENST00000507730 | RP11-321E2.3 | -3.632181 | 4.82E-12 |
| ASHGA5P049097 | noncoding | TCONS_00023857 | XLOC_011172 | -3.6371837 | 0.00000071 |
| ASHGA5P023060 | noncoding | ENST00000568831 | CTD-2049O4.1 | -3.6386217 | 0.00000106 |
| ASHGA5P035026 | noncoding | uc002rwa.2 | AK056077 | -3.6441349 | 0.00000233 |
| ASHGA5P028010 | noncoding | NR_027358 | LINC00592 | -3.6499979 | 0.0000104 |
| ASHGA5P017389 | noncoding | ENST00000440921 | RP4-753D10.3 | -3.6528364 | 2.1E-12 |
| ASHGA5P056908 | noncoding | TCONS_00010430 | XLOC_004945 | -3.6560066 | 0.0000011 |
| ASHGA5P053779 | noncoding | uc004bbl.1 | AK094413 | -3.6611153 | 0.00000339 |
| ASHGA5P045389 | noncoding | uc004bdx.1 | AX747119 | -3.6630289 | 3.52E-08 |
| ASHGA5P053586 | noncoding | NR_026913 | LOC100133669 | -3.664776 | 0.0000105 |
| ASHGA5P020449 | noncoding | NR_038402 | LOC100507387 | -3.6701185 | 8.89E-11 |
| ASHGA5P042072 | noncoding | ENST00000564248 | RP3-406A7.7 | -3.6931819 | 2.56E-08 |
| ASHGA5P039132 | noncoding | ENST00000498616 | RP11-85M11.2 | -3.6961113 | 0.000304 |
| ASHGA5P026059 | noncoding | ENST00000416725 | AP003039.3 | -3.6963549 | 9.15E-10 |
| ASHGA5P037302 | noncoding | uc002ywn.1 | AL109792 | -3.7050607 | 0.000045 |
| ASHGA5P016559 | noncoding | ENST00000436975 | RP11-245J24.1 | -3.7245383 | 8.89E-08 |
| ASHGA5P044219 | noncoding | uc003xng.4 | ADAM3A | -3.728283 | 0.000000063 |
| ASHGA5P042073 | noncoding | uc003qhb.3 | AB073649 | -3.7365749 | 0.00001 |
| ASHGA5P015357 | noncoding | ENST00000419604 | RP11-64P14.7 | -3.7448246 | 0.0000101 |
| ASHGA5P034863 | noncoding | NR_033997 | RNF144A-AS1 | -3.7574034 | 0.000000103 |
| ASHGA5P056163 | noncoding | uc001yif.1 | AK123878 | -3.8204039 | 0.0000502 |
| ASHGA5P030131 | noncoding | ENST00000561571 | RP11-673C5.2 | -3.823461 | 5.76E-09 |
| ASHGA5P026909 | noncoding | uc001qeh.3 | BC030092 | -3.8250767 | 0.00000024 |
| ASHGA5P031864 | noncoding | TCONS_00006579 | XLOC_003201 | -3.8722748 | 3.56E-12 |
| ASHGA5P036131 | noncoding | ENST00000564121 | RP11-521O16.2 | -3.9059348 | 4.39E-12 |
| ASHGA5P039796 | noncoding | ENST00000508601 | RP11-586D19.1 | -3.9125601 | 0.000000325 |
| ASHGA5P045259 | noncoding | ENST00000424682 | RP11-58A12.2 | -3.918555 | 0.00000291 |
| ASHGA5P036888 | noncoding | uc002yex.3 | KCNQ2 | -3.9197822 | 0.00000634 |
| ASHGA5P026981 | noncoding | uc001qmd.1 | AK125333 | -3.9232182 | 0.00000126 |
| ASHGA5P034551 | noncoding | NR_040036 | LOC100505835 | -3.9326798 | 0.0000911 |
| ASHGA5P057454 | noncoding | TCONS_00017618 | XLOC_008306 | -3.9737112 | 4.16E-14 |
| ASHGA5P032743 | noncoding | ENST00000536214 | DBIL5P | -4.0341276 | 0.000000554 |
| ASHGA5P041561 | noncoding | ENST00000510029 | RP11-826N14.4 | -4.0642157 | 2.42E-12 |
| ASHGA5P039402 | noncoding | ENST00000568817 | RP11-320H14.1 | -4.083756 | 0.00000239 |
| ASHGA5P032267 | noncoding | ENST00000580113 | KRT16P3 | -4.089001 | 1.26E-11 |
| ASHGA5P047732 | noncoding | ENST00000413810 | RP11-245J24.1 | -4.0916999 | 0.0000012 |
| ASHGA5P037796 | noncoding | NR_026651 | DGCR10 | -4.0935308 | 0.000278 |
| ASHGA5P054492 | noncoding | TCONS_00010429 | XLOC_004945 | -4.135443 | 4.26E-08 |
| ASHGA5P028418 | noncoding | ENST00000443554 | LINC00420 | -4.1438188 | 0.0000497 |
| ASHGA5P047683 | noncoding | uc001kyd.1 | AK094859 | -4.1491428 | 1.99E-09 |
| ASHGA5P040534 | noncoding | ENST00000505694 | CTC-459I6.1 | -4.1552378 | 1.73E-11 |
| ASHGA5P040348 | noncoding | TCONS_00021787 | XLOC_010385 | -4.1735442 | 8.8E-10 |
| ASHGA5P050927 | noncoding | ENST00000419734 | RP4-753D10.3 | -4.1782113 | 2.16E-11 |
| ASHGA5P045824 | noncoding | ENST00000465736 | ANKRD19P | -4.2297709 | 4.94E-10 |
| ASHGA5P041449 | noncoding | ENST00000566630 | CTB-85P21.2 | -4.237435 | 2.61E-08 |
| ASHGA5P044704 | noncoding | ENST00000511897 | RP11-87E22.2 | -4.2493948 | 7.34E-10 |
| ASHGA5P044511 | noncoding | ENST00000520031 | FER1L6-AS2 | -4.2695141 | 1.66E-09 |
| ASHGA5P036070 | noncoding | ENST00000458252 | AC123886.2 | -4.2835696 | 0.000000079 |
| ASHGA5P052561 | noncoding | NR_026970 | LY86-AS1 | -4.3012355 | 1.25E-15 |
| ASHGA5P057658 | noncoding | TCONS_00020497 | XLOC_009819 | -4.3511306 | 0.00000501 |
| ASHGA5P015074 | noncoding | ENST00000416330 | XIST | -4.4036622 | 0.0000702 |
| ASHGA5P038440 | noncoding | ENST00000441830 | RP4-677H15.2 | -4.469663 | 0.000000112 |
| ASHGA5P017787 | noncoding | ENST00000445739 | AC011322.1 | -4.5050987 | 9.59E-14 |
| ASHGA5P051567 | noncoding | ENST00000492031 | RP11-147N17.1 | -5.3589168 | 5.5E-12 |
| ASHGA5P037647 | noncoding | NR_002727 | RFPL1-AS1 | -5.4447976 | 4.09E-12 |
| ASHGA5P041105 | noncoding | ENST00000509844 | CTD-2023N9.3 | -5.6468642 | 1.12E-09 |
| ASHGA5P030859 | noncoding | ENST00000570022 | LA16c-329F2.1 | -5.6553464 | 1.05E-08 |
| ASHGA5P035259 | noncoding | NR_036580 | LOC389023 | -5.6868093 | 0.0000018 |
| ASHGA5P053261 | noncoding | NR_028137 | SLC26A4-AS1 | -5.7040063 | 3.61E-15 |
| ASHGA5P036871 | noncoding | ENST00000447909 | RP11-429E11.2 | -5.70762 | 2.38E-13 |
| ASHGA5P021024 | noncoding | ENST00000523557 | RP11-946L20.2 | -5.7736826 | 5.95E-09 |
| ASHGA5P057659 | noncoding | TCONS_00020498 | XLOC_009819 | -5.8105635 | 0.00000113 |
| ASHGA5P040862 | noncoding | ENST00000522956 | CTB-78F1.1 | -5.8194774 | 3.48E-12 |
| ASHGA5P032216 | noncoding | ENST00000562897 | RP11-416I2.1 | -5.8262715 | 7.37E-14 |
| ASHGA5P038088 | noncoding | ENST00000449158 | GRM7-AS3 | -5.893162 | 5.5E-11 |
| ASHGA5P037218 | noncoding | uc002ylb.4 | LINC00320 | -5.9199018 | 0.000000317 |
| ASHGA5P048008 | noncoding | ENST00000561816 | RP11-215H22.1 | -5.9307056 | 7.83E-10 |
| ASHGA5P027435 | noncoding | NR_026836 | TRHDE-AS1 | -5.9309603 | 2.71E-14 |
| ASHGA5P047177 | noncoding | ENST00000441365 | RP11-288G11.3 | -5.9503375 | 1.16E-08 |
| ASHGA5P041888 | noncoding | ENST00000511849 | RP1-240B8.3 | -5.9836229 | 8.21E-11 |
| ASHGA5P007912 | noncoding | ENST00000492083 | ANKRD19P | -6.0272044 | 1.43E-11 |
| ASHGA5P045179 | noncoding | uc003zqt.3 | BX538226 | -6.0401907 | 7.53E-08 |
| ASHGA5P054034 | noncoding | uc004ebm.1 | XIST | -6.0526159 | 0.000196 |
| ASHGA5P034138 | noncoding | NR_040029 | LOC284395 | -6.0582834 | 8.25E-10 |
| ASHGA5P032272 | noncoding | NR_027086 | LOC284578 | -6.0592056 | 6.71E-11 |
| ASHGA5P040462 | noncoding | ENST00000502882 | RP11-158J3.2 | -6.1020531 | 3.08E-10 |
| ASHGA5P045827 | noncoding | ENST00000464387 | ANKRD19P | -6.1665473 | 0.000000018 |
| ASHGA5P041656 | noncoding | ENST00000447858 | LY86-AS1 | -6.1949848 | 5.32E-12 |
| ASHGA5P015552 | noncoding | ENST00000421322 | XIST | -6.2182857 | 0.000202 |
| ASHGA5P047181 | noncoding | ENST00000443633 | RP13-137A17.4 | -6.2744636 | 2.74E-14 |
| ASHGA5P045063 | noncoding | ENST00000562989 | RP11-953B20.1 | -6.3493107 | 3.89E-11 |
| ASHGA5P038897 | noncoding | ENST00000562191 | RP11-389G6.3 | -6.4693656 | 9.77E-09 |
| ASHGA5P033941 | noncoding | ENST00000382445 | AC005329.6 | -6.4843396 | 2.08E-09 |
| ASHGA5P038438 | noncoding | ENST00000565615 | RP4-677H15.6 | -6.5671117 | 0.000000118 |
| ASHGA5P052176 | noncoding | ENST00000504989 | RP11-325I22.2 | -6.5719223 | 5.49E-08 |
| ASHGA5P044151 | noncoding | ENST00000518967 | RP11-369E15.3 | -6.6573873 | 2.79E-09 |
| ASHGA5P042704 | noncoding | ENST00000442328 | SYNJ2-IT1 | -7.3129924 | 8.59E-10 |
| ASHGA5P029774 | noncoding | NR_046473 | MEG3 | -7.7790554 | 0.00000276 |
| ASHGA5P046580 | noncoding | ENST00000454388 | RP11-493K23.4 | -8.1950812 | 5.61E-09 |
| ASHGA5P038755 | noncoding | NR_003090 | MOBP | -8.6298022 | 0.000000362 |
| ASHGA5P052201 | noncoding | ENST00000508677 | RP11-321E2.3 | -9.6855242 | 9.06E-12 |
| ASHGA5P016837 | noncoding | ENST00000434839 | XIST | -10.0874837 | 0.000162 |
| ASHGA5P047192 | noncoding | ENST00000451601 | RP11-809C18.3 | -11.6589882 | 1.51E-08 |
