# Supplementary material for: LINC01287 facilitates proliferation, migration, invasion and EMT of colon cancer cells via miR-4500/MAP3K13 pathway
Source: BMC Cancer. 2021 Jul 6;21:782. doi: 10.1186/s12885-021-08528-7 (PMC8259379; doi:10.1186/s12885-021-08528-7)
Supplement: Supplementary file 2 — Additional file 2: Supplementary Table 2. Differentially regulated mRNAs between colon cancer tissues (CC) vs paired normal samples. [file 12885_2021_8528_MOESM2_ESM.docx]

**Supplementary Table 2. Differentially regulated mRNAs between colon cancer tissues (CC) vs paired normal samples.**

| **ProbeName** | **type** | **seqname** | **Gene Symbol** | **Log2 fold change** | **p-value** |
| --- | --- | --- | --- | --- | --- |
| ASHGA5P026237 | protein_coding | NM_199161 | SAA1 | 11.5144108 | 0.000000362 |
| ASHGA5P002498 | protein_coding | NM_002423 | MMP7 | 9.9073263 | 6.723E-07 |
| ASHGA5P001861 | protein_coding | NM_014750 | DLGAP5 | 9.5120625 | 3.442E-17 |
| ASHGA5P052739 | protein_coding | NM_001025369 | VEGFA | 8.8310612 | 0.000001021 |
| ASHGA5P002637 | protein_coding | ENST00000261597 | NDC80 | 8.3132666 | 2.701E-17 |
| ASHGA5P004245 | protein_coding | NM_004701 | CCNB2 | 7.9919338 | 3.648E-13 |
| ASHGA5P005372 | protein_coding | NM_001034 | RRM2 | 7.8356898 | 9.833E-13 |
| ASHGA5P002292 | protein_coding | NM_030754 | SAA2 | 7.726094 | 0.000002605 |
| ASHGA5P042294 | protein_coding | ENST00000356350 | HIST1H2BH | 7.5316174 | 8.413E-09 |
| ASHGA5P012735 | protein_coding | NM_001029989 | KIAA0101 | 7.2900303 | 1.613E-13 |
| ASHGA5P054207 | protein_coding | NM_001008 | RPS4Y1 | 6.9323197 | 0.0002833 |
| ASHGA5P009162 | protein_coding | NM_181800 | UBE2C | 6.8694843 | 1.556E-12 |
| ASHGA5P011691 | protein_coding | NM_014467 | SRPX2 | 6.8684168 | 3.792E-07 |
| ASHGA5P014133 | protein_coding | NM_001166017 | SKA3 | 6.8608984 | 3.466E-14 |
| ASHGA5P010424 | protein_coding | ENST00000361365 | EIF1AY | 6.7409306 | 0.0007741 |
| ASHGA5P048637 | protein_coding | NM_001211 | BUB1B | 6.7301407 | 1.764E-11 |
| ASHGA5P004997 | protein_coding | NM_014736 | KIAA0101 | 6.714185 | 1.274E-10 |
| ASHGA5P002921 | protein_coding | ENST00000263398 | CD44 | 6.6688979 | 7.707E-12 |
| ASHGA5P046260 | protein_coding | NM_021992 | TMSB15A | 6.5258658 | 2.793E-07 |
| ASHGA5P040010 | protein_coding | NM_004967 | IBSP | 6.4840903 | 0.00001816 |
| ASHGA5P003968 | protein_coding | NM_024629 | MLF1IP | 6.4683303 | 1.055E-08 |
| ASHGA5P013631 | protein_coding | ENST00000395284 | CDK1 | 6.4590371 | 0.000009749 |
| ASHGA5P050616 | protein_coding | NM_004336 | BUB1 | 6.4538207 | 4.357E-11 |
| ASHGA5P049664 | protein_coding | NM_001067 | TOP2A | 6.4517777 | 5.578E-14 |
| ASHGA5P013793 | protein_coding | NM_203416 | CD163 | 6.4263367 | 3.143E-09 |
| ASHGA5P003851 | protein_coding | NM_001001392 | CD44 | 6.3875426 | 1.476E-09 |
| ASHGA5P001735 | protein_coding | NM_181803 | UBE2C | 6.302347 | 5.235E-13 |
| ASHGA5P001278 | protein_coding | NM_000088 | COL1A1 | 6.2584108 | 1.448E-07 |
| ASHGA5P008008 | protein_coding | NM_001032281 | TFPI | 6.2464081 | 2.385E-14 |
| ASHGA5P005133 | protein_coding | NM_001012271 | BIRC5 | 6.1366179 | 1.014E-14 |
| ASHGA5P010085 | protein_coding | NM_004244 | CD163 | 6.0855919 | 1.049E-07 |
| ASHGA5P001763 | protein_coding | NM_018643 | TREM1 | 6.0356596 | 4.808E-08 |
| ASHGA5P003991 | protein_coding | NM_020675 | SPC25 | 6.0144384 | 3.211E-14 |
| ASHGA5P001411 | protein_coding | NM_001025368 | VEGFA | 6.0099786 | 0.000006993 |
| ASHGA5P006283 | protein_coding | NM_033379 | CDK1 | 6.0068394 | 1.354E-07 |
| ASHGA5P045809 | protein_coding | ENST00000314355 | CKS2 | 6.0033815 | 1.219E-11 |
| ASHGA5P006058 | protein_coding | NM_006342 | TACC3 | 5.9772552 | 1.098E-10 |
| ASHGA5P005165 | protein_coding | NM_018492 | PBK | 5.9754308 | 1.425E-07 |
| ASHGA5P002024 | protein_coding | ENST00000407724 | CYorf15A | 5.944737 | 0.00007969 |
| ASHGA5P015748 | protein_coding | NM_001164270 | RAD51 | 5.9427762 | 2.397E-11 |
| ASHGA5P005699 | protein_coding | NM_019013 | FAM64A | 5.9293646 | 4.003E-07 |
| ASHGA5P001734 | protein_coding | NM_007019 | UBE2C | 5.923863 | 2.055E-08 |
| ASHGA5P048654 | protein_coding | ENST00000424227 | MAP3K13 | 5.8709255 | 6.288E-08 |
| ASHGA5P036436 | protein_coding | NM_000597 | IGFBP2 | 5.8165146 | 0.0007687 |
| ASHGA5P020039 | protein_coding | NM_000900 | MGP | 5.7957523 | 0.0000106 |
| ASHGA5P035630 | protein_coding | NM_057165 | COL6A3 | 5.7829553 | 0.000003321 |
| ASHGA5P004538 | protein_coding | NM_001006624 | PDPN | 5.74787 | 0.000000607 |
| ASHGA5P004987 | protein_coding | NM_006169 | NNMT | 5.7353529 | 0.00003107 |
| ASHGA5P001039 | protein_coding | NM_003254 | TIMP1 | 5.6454317 | 5.347E-08 |
| ASHGA5P007624 | protein_coding | NM_181799 | UBE2C | 5.6202053 | 1.417E-09 |
| ASHGA5P056043 | protein_coding | NM_016445 | PLEK2 | 5.5929619 | 4.67E-08 |
| ASHGA5P002214 | protein_coding | NM_001276 | CHI3L1 | 5.5619039 | 0.0059837 |
| ASHGA5P032717 | protein_coding | NM_005782 | ALYREF | 4.7521234 | 4.043E-07 |
| ASHGA5P016882 | protein_coding | NM_001131019 | GFAP | 4.6439393 | 0.000003565 |
| ASHGA5P048228 | protein_coding | ENST00000311330 | CD248 | 4.5415461 | 4.149E-07 |
| ASHGA5P004047 | protein_coding | ENST00000283006 | CENPH | 4.5329626 | 1.71E-09 |
| ASHGA5P002993 | protein_coding | NM_012110 | CHIC2 | 4.5213255 | 0.000007369 |
| ASHGA5P008479 | protein_coding | NM_198947 | FAM111B | 4.5132681 | 2.024E-08 |
| ASHGA5P055067 | protein_coding | NM_007173 | PRSS23 | 4.5076144 | 6.811E-07 |
| ASHGA5P036291 | protein_coding | NM_000885 | ITGA4 | 4.4678496 | 6.614E-09 |
| ASHGA5P001365 | protein_coding | NM_031299 | CDCA3 | 4.4518431 | 1.751E-08 |
| ASHGA5P029413 | protein_coding | ENST00000315947 | OR4N2 | 4.4387406 | 0.0040978 |
| ASHGA5P052740 | protein_coding | NM_001033756 | VEGFA | 4.4372597 | 0.0000735 |
| ASHGA5P013499 | protein_coding | NM_152637 | METTL7B | 4.4353773 | 0.00004849 |
| ASHGA5P047590 | protein_coding | NM_004523 | KIF11 | 4.4159619 | 1.5E-11 |
| ASHGA5P012657 | protein_coding | NM_001135935 | POSTN | 4.3914912 | 0.0183597 |
| ASHGA5P008007 | protein_coding | NM_001005376 | PLAUR | 4.3914602 | 8.801E-11 |
| ASHGA5P007413 | protein_coding | NM_001002858 | ANXA2 | 4.3868018 | 0.0011448 |
| ASHGA5P052051 | protein_coding | NM_002358 | MAD2L1 | 4.3852294 | 0.000001856 |
| ASHGA5P012153 | protein_coding | NM_001006625 | PDPN | 4.3736 | 1.198E-07 |
| ASHGA5P002293 | protein_coding | NM_013409 | FST | 4.3699437 | 0.000004891 |
| ASHGA5P010405 | protein_coding | ENST00000378714 | MCM10 | 4.3686031 | 0.000008952 |
| ASHGA5P014954 | protein_coding | NM_001001389 | CD44 | 4.3639976 | 0.0002635 |
| ASHGA5P055996 | protein_coding | NM_005192 | CDKN3 | 4.3600766 | 0.000005663 |
| ASHGA5P012017 | protein_coding | NM_001161728 | PLA2G2A | 4.3563192 | 0.0026275 |
| ASHGA5P002327 | protein_coding | NM_000700 | ANXA1 | 4.3558334 | 0.00000547 |
| ASHGA5P009023 | protein_coding | NM_001168 | BIRC5 | 4.3531582 | 1.634E-08 |
| ASHGA5P048006 | protein_coding | NM_031217 | KIF18A | 4.3499417 | 1.519E-08 |
| ASHGA5P019546 | protein_coding | NM_001100603 | KDELR2 | 4.3427539 | 3.701E-10 |
| ASHGA5P038692 | protein_coding | ENST00000383807 | FANCD2 | 4.3327493 | 3.26E-08 |
| ASHGA5P052484 | protein_coding | NM_004219 | PTTG1 | 4.3241766 | 1.334E-10 |
| ASHGA5P043816 | protein_coding | NM_002593 | PCOLCE | 4.319215 | 4.021E-08 |
| ASHGA5P008539 | protein_coding | NM_013445 | GAD1 | 4.3181079 | 0.00002009 |
| ASHGA5P036612 | protein_coding | NM_019609 | CPXM1 | 4.3123291 | 0.000008029 |
| ASHGA5P011822 | protein_coding | NM_002895 | RBL1 | 4.2966309 | 5.376E-09 |
| ASHGA5P001988 | protein_coding | NM_005570 | LMAN1 | 4.2874446 | 8.381E-07 |
| ASHGA5P002271 | protein_coding | NM_031966 | CCNB1 | 4.2857001 | 8.107E-13 |
| ASHGA5P012656 | protein_coding | NM_001135934 | POSTN | 4.2838118 | 0.0065184 |
| ASHGA5P041724 | protein_coding | ENST00000359611 | HIST1H2AM | 4.2800756 | 5.706E-08 |
| ASHGA5P013543 | protein_coding | NM_205858 | NMB | 4.2696948 | 1.7E-09 |
| ASHGA5P005915 | protein_coding | NM_000237 | LPL | 4.2661924 | 0.0058256 |
| ASHGA5P051917 | protein_coding | NM_006607 | PTTG2 | 4.2651724 | 1.568E-09 |
| ASHGA5P012397 | protein_coding | NM_024808 | BORA | 4.2617591 | 1.144E-13 |
| ASHGA5P042316 | protein_coding | ENST00000244623 | OR2B6 | 4.2559955 | 1.643E-08 |
| ASHGA5P009874 | protein_coding | NM_152524 | SGOL2 | 4.2392437 | 0.000001021 |
| ASHGA5P018185 | protein_coding | ENST00000450318 | NUSAP1 | 4.2339791 | 0.000006105 |
| ASHGA5P055050 | protein_coding | NM_145018 | C11orf82 | 4.2284742 | 6.583E-12 |
| ASHGA5P051736 | protein_coding | NM_001167916 | VEPH1 | 4.2231349 | 0.000005051 |
| ASHGA5P038797 | protein_coding | ENST00000296435 | CAMP | 4.2222824 | 0.00004376 |
| ASHGA5P012185 | protein_coding | NM_002668 | PLP2 | 4.2129749 | 1.979E-07 |
| ASHGA5P038221 | protein_coding | ENST00000434006 | SPINK8 | 4.2061657 | 0.000001045 |
| ASHGA5P002247 | protein_coding | NM_002677 | PMP2 | 4.2040751 | 0.00008361 |
| ASHGA5P038260 | protein_coding | ENST00000371116 | FOXD3 | 4.1970092 | 0.000005474 |
| ASHGA5P010274 | protein_coding | NM_021077 | NMB | 4.1894947 | 2.224E-09 |
| ASHGA5P052061 | protein_coding | NM_002966 | S100A10 | 4.1842863 | 7.567E-08 |
| ASHGA5P053280 | protein_coding | NM_001172895 | CAV1 | 4.1830046 | 0.00002518 |
| ASHGA5P046781 | protein_coding | NM_020420 | DAZ4 | 4.1773551 | 0.017922 |
| ASHGA5P004912 | protein_coding | NM_153687 | IKBIP | 4.1767363 | 0.00006622 |
| ASHGA5P004676 | protein_coding | NM_002090 | CXCL3 | 4.1556972 | 0.0004012 |
| ASHGA5P044787 | protein_coding | NM_020130 | C8orf4 | 4.1490967 | 0.000002537 |
| ASHGA5P038132 | protein_coding | NM_001012409 | SGOL1 | 4.138559 | 0.000001179 |
| ASHGA5P052291 | protein_coding | NM_004472 | FOXD1 | 4.1377687 | 0.000005477 |
| ASHGA5P042896 | protein_coding | NM_006854 | KDELR2 | 4.1356851 | 1.161E-08 |
| ASHGA5P013596 | protein_coding | NM_001161580 | POC1A | 4.1194916 | 9.711E-11 |
| ASHGA5P046829 | protein_coding | NM_001039567 | RPS4Y2 | 4.1031529 | 0.0006819 |
| ASHGA5P003211 | protein_coding | NM_003873 | NRP1 | 4.0939416 | 1.385E-07 |
| ASHGA5P052902 | protein_coding | NM_000636 | SOD2 | 4.0869736 | 3.914E-08 |
| ASHGA5P010739 | protein_coding | NM_001001552 | LEMD1 | 4.0817493 | 0.00004897 |
| ASHGA5P003543 | protein_coding | ENST00000270221 | EMP3 | 4.0706071 | 2.302E-07 |
| ASHGA5P011980 | protein_coding | NM_001024629 | NRP1 | 4.0661493 | 3.787E-07 |
| ASHGA5P000910 | protein_coding | NM_006855 | KDELR3 | 4.0643427 | 0.0002693 |
| ASHGA5P051979 | protein_coding | NM_001657 | AREG | 4.061491 | 0.00001306 |
| ASHGA5P028498 | protein_coding | NM_006475 | POSTN | 3.8750073 | 0.0138067 |
| ASHGA5P041719 | protein_coding | ENST00000357549 | HIST1H4K | 3.8546364 | 0.000002782 |
| ASHGA5P018395 | protein_coding | NM_001145208 | DEPDC1B | 3.8487583 | 0.0001835 |
| ASHGA5P005502 | protein_coding | NM_000943 | PPIC | 3.8479016 | 0.00002372 |
| ASHGA5P002325 | protein_coding | NM_001142477 | NREP | 3.84531 | 1.443E-07 |
| ASHGA5P007833 | protein_coding | NM_001024466 | SOD2 | 3.841395 | 0.00004556 |
| ASHGA5P050866 | protein_coding | ENST00000392003 | COL6A3 | 3.8279445 | 0.000003808 |
| ASHGA5P005888 | protein_coding | NM_001321 | CSRP2 | 3.8188743 | 0.0001669 |
| ASHGA5P037074 | protein_coding | NM_002638 | PI3 | 3.8042766 | 0.0001041 |
| ASHGA5P052132 | protein_coding | NM_001130689 | HMGB2 | 3.791707 | 0.007194 |
| ASHGA5P021073 | protein_coding | NM_148170 | CTSC | 3.7758312 | 9.589E-07 |
| ASHGA5P037348 | protein_coding | ENST00000291560 | HSF2BP | 3.7647464 | 6.075E-09 |
| ASHGA5P006793 | protein_coding | NM_152852 | MS4A6A | 3.7564196 | 1.934E-08 |
| ASHGA5P042313 | protein_coding | NM_021968 | HIST1H4J | 3.7550472 | 0.000005175 |
| ASHGA5P054415 | protein_coding | NM_001024628 | NRP1 | 3.7542691 | 2.615E-07 |
| ASHGA5P010947 | protein_coding | NM_001012507 | CENPW | 3.7459924 | 6.194E-09 |
| ASHGA5P039448 | protein_coding | NM_002704 | PPBP | 3.7448337 | 0.0004396 |
| ASHGA5P007594 | protein_coding | NM_206886 | CCDC18 | 3.738105 | 4.622E-08 |
| ASHGA5P005188 | protein_coding | NM_002998 | SDC2 | 3.7275319 | 0.001273 |
| ASHGA5P001237 | protein_coding | NM_003380 | VIM | 3.7247675 | 2.135E-08 |
| ASHGA5P046226 | protein_coding | NM_013346 | SNX12 | 3.7214729 | 1.464E-08 |
| ASHGA5P001712 | protein_coding | NM_000640 | IL13RA2 | 3.7189525 | 0.0013221 |
| ASHGA5P002277 | protein_coding | NM_003670 | BHLHE40 | 3.7136827 | 0.0001519 |
| ASHGA5P051129 | protein_coding | NM_170736 | KCNJ15 | 3.2580213 | 0.00002759 |
| ASHGA5P008376 | protein_coding | NM_201612 | IKBIP | 3.2526336 | 0.0007423 |
| ASHGA5P041323 | protein_coding | ENST00000368738 | S100A9 | 3.2525611 | 0.0005292 |
| ASHGA5P035937 | protein_coding | NM_001615 | ACTG2 | 3.2492691 | 0.0017555 |
| ASHGA5P007881 | protein_coding | NM_148975 | MS4A4A | 3.2489504 | 0.0003042 |
| ASHGA5P053087 | protein_coding | NM_201283 | EGFR | 3.242356 | 0.0029714 |
| ASHGA5P009067 | protein_coding | ENST00000350896 | MSR1 | 3.2419396 | 0.00001508 |
| ASHGA5P005575 | protein_coding | ENST00000307407 | IL8 | 3.1507851 | 0.012217 |
| ASHGA5P040826 | protein_coding | ENST00000331380 | HIST2H2AC | 3.148329 | 0.00001077 |
| ASHGA5P005387 | protein_coding | NM_001002033 | HN1 | 3.1412692 | 1.281E-10 |
| ASHGA5P055115 | protein_coding | NM_001191016 | CASP12 | 3.1201477 | 0.000003491 |
| ASHGA5P011240 | protein_coding | NM_001839 | CNN3 | 3.1136893 | 0.0058249 |
| ASHGA5P013744 | protein_coding | NM_001002843 | ZNF280D | 3.1064467 | 9.786E-13 |
| ASHGA5P027372 | protein_coding | NM_000075 | CDK4 | 3.1057479 | 0.000003153 |
| ASHGA5P040456 | protein_coding | NM_018369 | DEPDC1B | 3.0945357 | 0.00000515 |
| ASHGA5P001562 | protein_coding | NM_001002274 | FCGR2B | 3.0656784 | 0.000289 |
| ASHGA5P051971 | protein_coding | NM_144646 | IGJ | 3.0575317 | 0.0006046 |
| ASHGA5P008744 | protein_coding | NM_212474 | FN1 | 3.0568216 | 0.0171314 |
| ASHGA5P005063 | protein_coding | NM_003414 | ZNF267 | 3.0553054 | 5.975E-10 |
| ASHGA5P009596 | protein_coding | NM_001002032 | HN1 | 3.0542369 | 3.48E-10 |
| ASHGA5P052866 | protein_coding | NM_004833 | AIM2 | 3.050118 | 0.00000339 |
| ASHGA5P003252 | protein_coding | NM_015973 | GAL | 3.05005 | 0.0001304 |
| ASHGA5P010696 | protein_coding | NM_138796 | SPATA17 | 3.0491925 | 2.095E-10 |
| ASHGA5P003151 | protein_coding | NM_001029998 | SLC10A7 | 3.0360333 | 7.015E-10 |
| ASHGA5P039600 | protein_coding | NM_001114734 | PABPC4L | 3.0310932 | 0.000001456 |
| ASHGA5P012857 | protein_coding | NM_001122898 | CD99 | 3.0305571 | 0.0008778 |
| ASHGA5P052412 | protein_coding | NM_005733 | KIF20A | 3.023117 | 0.0008011 |
| ASHGA5P010288 | protein_coding | NM_001935 | DPP4 | 3.0215151 | 0.000296 |
| ASHGA5P004029 | protein_coding | NM_022173 | TIA1 | 3.004898 | 0.000004694 |
| ASHGA5P050689 | protein_coding | ENST00000457476 | TANK | 3.002432 | 8.678E-09 |
| ASHGA5P001113 | protein_coding | NM_001005377 | PLAUR | 3.0001062 | 0.000006501 |
| ASHGA5P054867 | protein_coding | NM_022349 | MS4A6A | 2.999989 | 0.00000601 |
| ASHGA5P001414 | protein_coding | NM_001105206 | LAMA4 | 2.9983918 | 1.335E-07 |
| ASHGA5P017952 | protein_coding | NM_001001548 | CD36 | 2.9888922 | 0.0001395 |
| ASHGA5P052608 | protein_coding | NM_021066 | HIST1H2AJ | 2.9832223 | 0.0004598 |
| ASHGA5P039666 | protein_coding | NM_001025199 | CHI3L2 | 2.9830125 | 0.0003251 |
| ASHGA5P001313 | protein_coding | NM_015368 | PANX1 | 2.9824496 | 4.506E-07 |
| ASHGA5P041986 | protein_coding | NM_001105209 | LAMA4 | 2.9764595 | 1.782E-07 |
| ASHGA5P051010 | protein_coding | NM_181802 | UBE2C | 2.9731731 | 6.495E-07 |
| ASHGA5P052593 | protein_coding | NM_003530 | HIST1H3D | 2.9703203 | 1.017E-07 |
| ASHGA5P020512 | protein_coding | NM_001146337 | AFAP1L1 | 2.9696006 | 0.000001689 |
| ASHGA5P001426 | protein_coding | NM_003118 | SPARC | 2.9665311 | 0.00006296 |
| ASHGA5P053190 | protein_coding | NM_024553 | CCDC132 | 2.9660318 | 1.923E-10 |
| ASHGA5P007938 | protein_coding | ENST00000338458 | ARHGEF3 | 2.9653062 | 0.000003125 |
| ASHGA5P038261 | protein_coding | NM_015426 | POC1A | 2.9645362 | 8.74E-11 |
| ASHGA5P007983 | protein_coding | NM_004095 | EIF4EBP1 | 2.9634001 | 0.000001412 |
| ASHGA5P007238 | protein_coding | NM_001172309 | NEXN | 2.9574669 | 0.00002325 |
| ASHGA5P004660 | protein_coding | NM_002852 | PTX3 | 2.9569027 | 0.0048851 |
| ASHGA5P001543 | protein_coding | NM_018284 | GBP3 | 2.955713 | 0.0001986 |
| ASHGA5P035551 | protein_coding | NM_054034 | FN1 | 2.9511833 | 1.795E-07 |
| ASHGA5P002891 | protein_coding | NM_003504 | CDC45 | 2.9509012 | 0.00001669 |
| ASHGA5P044083 | protein_coding | NM_001118887 | ANGPT2 | 2.9457506 | 0.0002823 |
| ASHGA5P000619 | protein_coding | NM_006072 | CCL26 | 2.9435581 | 7.218E-07 |
| ASHGA5P021223 | protein_coding | NM_001114173 | CTSC | 2.9432388 | 0.00001108 |
| ASHGA5P025225 | protein_coding | NM_001242480 | LOC389831 | 2.9420039 | 0.0076932 |
| ASHGA5P029432 | protein_coding | NM_002934 | RNASE2 | 2.9398293 | 0.00008719 |
| ASHGA5P021382 | protein_coding | NM_001143836 | NOX4 | 2.9344969 | 0.0009269 |
| ASHGA5P016496 | protein_coding | ENST00000430935 | TTC26 | 2.9288988 | 0.000001849 |
| ASHGA5P008006 | protein_coding | NM_002659 | PLAUR | 2.925638 | 0.000003675 |
| ASHGA5P012858 | protein_coding | NM_002414 | CD99 | 2.9242872 | 0.0001347 |
| ASHGA5P021640 | protein_coding | NM_001165884 | ZNF268 | 2.9239965 | 3.34E-08 |
| ASHGA5P001928 | protein_coding | NM_005824 | LRRC17 | 2.9211238 | 0.0000188 |
| ASHGA5P014563 | protein_coding | NM_001142704 | FAM111B | 2.9158979 | 0.000002166 |
| ASHGA5P036360 | protein_coding | ENST00000286201 | FZD7 | 2.9133843 | 2.889E-07 |
| ASHGA5P046763 | protein_coding | NM_153716 | HSFY2 | 2.9121377 | 0.0153202 |
| ASHGA5P042417 | protein_coding | NM_002155 | HSPA6 | 2.9117368 | 4.663E-08 |
| ASHGA5P013132 | protein_coding | NM_001113561 | RNF180 | 2.9036562 | 0.000002894 |
| ASHGA5P015491 | protein_coding | NM_152851 | MS4A6A | 2.8985572 | 5.376E-07 |
| ASHGA5P006216 | protein_coding | NM_001122674 | ABCD3 | 2.8934704 | 0.00001793 |
| ASHGA5P027754 | protein_coding | NM_001759 | CCND2 | 2.8883905 | 0.00007184 |
| ASHGA5P013259 | protein_coding | NM_001146191 | MPZL1 | 2.880323 | 2.606E-12 |
| ASHGA5P002511 | protein_coding | NM_138555 | KIF23 | 2.8778396 | 0.0002343 |
| ASHGA5P041094 | protein_coding | NM_002104 | GZMK | 2.87637 | 0.0001121 |
| ASHGA5P054228 | protein_coding | ENST00000382832 | CYorf15B | 2.8737945 | 0.0009132 |
| ASHGA5P003035 | protein_coding | ENST00000264221 | PAICS | 2.8714213 | 0.0025274 |
| ASHGA5P014327 | protein_coding | NM_001163147 | ETV1 | 2.8701097 | 2.357E-08 |
| ASHGA5P001563 | protein_coding | NM_001190828 | FCGR2B | 2.8654421 | 0.0002924 |
| ASHGA5P036020 | protein_coding | NM_004633 | IL1R2 | 2.8625709 | 0.000101 |
| ASHGA5P049267 | protein_coding | NM_001755 | CBFB | 2.8604164 | 1.139E-10 |
| ASHGA5P054127 | protein_coding | NM_001166599 | FAM122B | 2.8594575 | 1.674E-09 |
| ASHGA5P010952 | protein_coding | NM_001044392 | MUC1 | 2.8580093 | 0.00001108 |
| ASHGA5P007687 | protein_coding | NM_001166109 | PALLD | 2.8534213 | 7.341E-08 |
| ASHGA5P001654 | protein_coding | NM_032117 | MND1 | 2.8502106 | 0.00001229 |
| ASHGA5P053542 | protein_coding | NM_002546 | TNFRSF11B | 2.8489874 | 0.0062459 |
| ASHGA5P011002 | protein_coding | ENST00000368733 | S100A8 | 2.847943 | 0.000165 |
| ASHGA5P003738 | protein_coding | NM_033515 | ARHGAP18 | 2.8451487 | 5.903E-07 |
| ASHGA5P036497 | protein_coding | NM_001130046 | CCL20 | 2.8431248 | 0.005468 |
| ASHGA5P030727 | protein_coding | NM_001113378 | FANCI | 2.8419743 | 2.209E-09 |
| ASHGA5P052220 | protein_coding | NM_182801 | EGFLAM | 2.8411681 | 0.0001127 |
| ASHGA5P041711 | protein_coding | ENST00000446824 | HIST1H3F | 2.8381879 | 0.0004431 |
| ASHGA5P013539 | protein_coding | NM_172129 | CAMK2D | 2.8361882 | 0.00006875 |
| ASHGA5P011365 | protein_coding | NM_001924 | GADD45A | 2.8348447 | 0.000991 |
| ASHGA5P039983 | protein_coding | NM_002620 | PF4V1 | 2.8344732 | 0.00003428 |
| ASHGA5P001500 | protein_coding | NM_000599 | IGFBP5 | 2.8303093 | 0.000009524 |
| ASHGA5P020658 | protein_coding | NM_001164753 | ASPH | 2.8274008 | 0.0001184 |
| ASHGA5P013497 | protein_coding | NM_003981 | PRC1 | 2.819483 | 0.0059307 |
| ASHGA5P001376 | protein_coding | NM_003769 | SRSF9 | 2.8155047 | 0.012254 |
| ASHGA5P006789 | protein_coding | NM_002026 | FN1 | 2.8147179 | 0.0110936 |
| ASHGA5P009595 | protein_coding | NM_016185 | HN1 | 2.8127595 | 1.131E-09 |
| ASHGA5P018469 | protein_coding | NM_001136139 | TCF3 | 2.810332 | 2.118E-12 |
| ASHGA5P008509 | protein_coding | NM_024021 | MS4A4A | 2.8097339 | 0.0003378 |
| ASHGA5P001922 | protein_coding | NM_016200 | NAA38 | 2.8056983 | 8.242E-11 |
| ASHGA5P001511 | protein_coding | NM_017958 | PLEKHB2 | 2.8003022 | 6.086E-07 |
| ASHGA5P042274 | protein_coding | NM_003107 | SOX4 | 2.795628 | 0.0000522 |
| ASHGA5P012254 | protein_coding | NM_006743 | RBM3 | 2.7929584 | 0.001323 |
| ASHGA5P000621 | protein_coding | NM_005402 | RALA | 2.7902653 | 0.0003851 |
| ASHGA5P011183 | protein_coding | ENST00000369849 | INA | 2.7891365 | 0.005967 |
| ASHGA5P002990 | protein_coding | NM_005168 | RND3 | 2.7882583 | 0.0002263 |
| ASHGA5P011250 | protein_coding | NM_016040 | TMED5 | 2.7852748 | 0.0002602 |
| ASHGA5P049785 | protein_coding | NM_182620 | SKA2 | 2.7847558 | 1.689E-09 |
| ASHGA5P003573 | protein_coding | NM_014288 | ITGB3BP | 2.7816881 | 9.877E-11 |
| ASHGA5P013342 | protein_coding | NM_001040651 | CD3D | 2.779393 | 0.0004647 |
| ASHGA5P008278 | protein_coding | NM_017994 | TMEM248 | 2.7793743 | 0.00001878 |
| ASHGA5P005288 | protein_coding | NM_005252 | FOS | 2.7661642 | 0.0152963 |
| ASHGA5P043349 | protein_coding | NM_001128619 | LUZP6 | 2.7656324 | 0.00001349 |
| ASHGA5P014286 | protein_coding | NM_001002876 | CENPM | 2.7620688 | 1.964E-08 |
| ASHGA5P002727 | protein_coding | NM_020648 | TWSG1 | 2.7596864 | 0.000001311 |
| ASHGA5P000946 | protein_coding | ENST00000216294 | SNAPC1 | 2.7589623 | 0.000006017 |
| ASHGA5P001330 | protein_coding | NM_005127 | CLEC2B | 2.7579845 | 0.000002564 |
| ASHGA5P008964 | protein_coding | NM_201517 | H2AFV | 2.7450227 | 1.293E-10 |
| ASHGA5P033090 | protein_coding | NM_001254 | CDC6 | 2.7423258 | 0.0000561 |
| ASHGA5P044408 | protein_coding | NM_012415 | RAD54B | 2.741252 | 0.0004462 |
| ASHGA5P052183 | protein_coding | NM_033267 | IRX2 | 2.7371249 | 0.0028629 |
| ASHGA5P041428 | protein_coding | ENST00000239444 | PCDHB8 | 2.7366846 | 0.0007116 |
| ASHGA5P053191 | protein_coding | NM_006528 | TFPI2 | 2.7354978 | 0.022608 |
| ASHGA5P003767 | protein_coding | NM_001145410 | NONO | 2.7332177 | 0.00000067 |
| ASHGA5P018489 | protein_coding | NM_001085400 | RELL1 | 2.7296382 | 4.67E-08 |
| ASHGA5P012422 | protein_coding | NM_021111 | RECK | 2.7241677 | 0.00002321 |
| ASHGA5P034538 | protein_coding | ENST00000343769 | ZNF93 | 2.7235701 | 0.000002731 |
| ASHGA5P051398 | protein_coding | NM_033084 | FANCD2 | 2.7221854 | 0.000001544 |
| ASHGA5P007114 | protein_coding | NM_007194 | CHEK2 | 2.7214136 | 0.0031938 |
| ASHGA5P052898 | protein_coding | NM_006519 | DYNLT1 | 2.716309 | 0.0003931 |
| ASHGA5P051235 | protein_coding | NM_138957 | MAPK1 | 2.7119044 | 1.519E-11 |
| ASHGA5P012844 | protein_coding | NM_201516 | H2AFV | 2.7099289 | 8.753E-14 |
| ASHGA5P012531 | protein_coding | NM_001114357 | C4orf47 | 2.7076937 | 0.0005259 |
| ASHGA5P041708 | protein_coding | ENST00000244601 | HIST1H2BG | 2.7069936 | 0.00000638 |
| ASHGA5P002723 | protein_coding | NM_138715 | MSR1 | 2.7058656 | 0.0005128 |
| ASHGA5P009691 | protein_coding | NM_198334 | GANAB | 2.6987262 | 0.00005266 |
| ASHGA5P006892 | protein_coding | NM_001147 | ANGPT2 | 2.6984147 | 0.0015706 |
| ASHGA5P001192 | protein_coding | NM_005868 | BET1 | 2.6900033 | 9.15E-09 |
| ASHGA5P042314 | protein_coding | NM_003511 | HIST1H2AL | 2.6899157 | 0.00004067 |
| ASHGA5P054718 | protein_coding | NM_001165030 | TMEM41B | 2.6898513 | 1.482E-08 |
| ASHGA5P003088 | protein_coding | NM_000096 | CP | 2.684158 | 0.0013936 |
| ASHGA5P007255 | protein_coding | NM_138455 | CTHRC1 | 2.6823818 | 0.0004795 |
| ASHGA5P014445 | protein_coding | NM_001080545 | PPP1R1C | 2.6791072 | 0.0004031 |
| ASHGA5P003191 | protein_coding | NM_020357 | PCNP | 2.6775325 | 0.000001752 |
| ASHGA5P013552 | protein_coding | NM_017918 | CCDC109B | 2.6774767 | 0.0001353 |
| ASHGA5P016677 | protein_coding | NM_001170751 | SRPX | 2.676332 | 0.026953 |
| ASHGA5P034087 | protein_coding | NM_033417 | HAUS8 | 2.6758595 | 0.000002923 |
| ASHGA5P003276 | protein_coding | NM_022170 | EIF4H | 2.6745353 | 0.00001769 |
| ASHGA5P039701 | protein_coding | NM_181643 | C1orf88 | 2.6737994 | 0.0010452 |
| ASHGA5P007922 | protein_coding | NM_001118888 | ANGPT2 | 2.6734402 | 0.0005844 |
| ASHGA5P011679 | protein_coding | NM_006733 | CENPI | 2.6674551 | 6.829E-10 |
| ASHGA5P012148 | protein_coding | NM_024089 | KDELC1 | 2.6659744 | 0.0032646 |
| ASHGA5P054662 | protein_coding | ENST00000405957 | LSP1 | 2.6654644 | 0.0046615 |
| ASHGA5P012611 | protein_coding | NM_182966 | NEDD9 | 2.661173 | 0.0000041 |
| ASHGA5P012096 | protein_coding | NM_052889 | CARD16 | 2.6610984 | 7.831E-07 |
| ASHGA5P001304 | protein_coding | NM_001165415 | LDHA | 2.6591578 | 0.0138783 |
| ASHGA5P015011 | protein_coding | NM_001039651 | SAPCD1 | 2.6556641 | 0.00008183 |
| ASHGA5P004831 | protein_coding | ENST00000297967 | NLGN4Y | 2.6490106 | 0.0005404 |
| ASHGA5P044882 | protein_coding | NM_015364 | LY96 | 2.6445699 | 0.000003165 |
| ASHGA5P001589 | protein_coding | NM_206963 | RARRES1 | 2.6433187 | 0.0010246 |
| ASHGA5P001131 | protein_coding | ENST00000221486 | RNASEH2A | 2.6384654 | 7.043E-10 |
| ASHGA5P015543 | protein_coding | NM_212478 | FN1 | 2.6366727 | 0.0312382 |
| ASHGA5P015059 | protein_coding | NM_001142761 | C15orf23 | 2.6361954 | 0.0000094 |
| ASHGA5P048693 | protein_coding | NM_002041 | GABPB1 | 2.6359834 | 3.053E-10 |
| ASHGA5P004677 | protein_coding | NM_002089 | CXCL2 | 2.6355996 | 0.0104525 |
| ASHGA5P052041 | protein_coding | ENST00000296402 | CAMK2D | 2.635512 | 0.000001887 |
| ASHGA5P040234 | protein_coding | NM_003864 | SAP30 | 2.6350558 | 0.000000694 |
| ASHGA5P043555 | protein_coding | NM_001172428 | KLHL7 | 2.6349699 | 8.601E-10 |
| ASHGA5P045305 | protein_coding | NM_002048 | GAS1 | 2.6334735 | 0.00002098 |
| ASHGA5P034539 | protein_coding | NM_052852 | ZNF486 | 2.6332275 | 9.335E-08 |
| ASHGA5P048680 | protein_coding | NM_032413 | C15orf48 | 2.6302713 | 0.0003536 |
| ASHGA5P036990 | protein_coding | ENST00000480798 | CST7 | 2.6296489 | 0.000004477 |
| ASHGA5P012568 | protein_coding | NM_182649 | PCNA | 2.6276978 | 0.0003895 |
| ASHGA5P050794 | protein_coding | NM_212476 | FN1 | 2.6273824 | 0.0144267 |
| ASHGA5P003917 | protein_coding | NM_001024074 | HNMT | 2.624549 | 6.159E-08 |
| ASHGA5P053038 | protein_coding | NM_015483 | KBTBD2 | 2.6203832 | 0.000004648 |
| ASHGA5P003034 | protein_coding | ENST00000264218 | NMU | 2.6195578 | 0.00005483 |
| ASHGA5P042530 | protein_coding | NM_012381 | ORC3 | 2.619124 | 1.531E-08 |
| ASHGA5P048694 | protein_coding | NM_005254 | GABPB1 | 2.6186743 | 0.0000297 |
| ASHGA5P033744 | protein_coding | NM_032243 | TXNDC2 | 2.6164985 | 0.000003575 |
| ASHGA5P048317 | protein_coding | NM_015424 | CHRDL2 | 2.61615 | 0.0022372 |
| ASHGA5P001962 | protein_coding | NM_014441 | SIGLEC9 | 2.6160405 | 0.000001763 |
| ASHGA5P027480 | protein_coding | NM_002345 | LUM | 2.6076088 | 0.04153 |
| ASHGA5P012419 | protein_coding | NM_022343 | GLIPR2 | 2.6051903 | 3.635E-09 |
| ASHGA5P003990 | protein_coding | NM_138443 | HAUS1 | 2.6025085 | 4.747E-08 |
| ASHGA5P027206 | protein_coding | ENST00000395510 | TWF1 | 2.5963484 | 0.0002354 |
| ASHGA5P007197 | protein_coding | NM_213674 | TPM2 | 2.5957007 | 1.886E-07 |
| ASHGA5P009645 | protein_coding | NM_007047 | BTN3A2 | 2.5929228 | 0.000003308 |
| ASHGA5P014015 | protein_coding | ENST00000398665 | DOT1L | 2.5884209 | 2.365E-07 |
| ASHGA5P052998 | protein_coding | ENST00000307471 | CCDC126 | 2.5883043 | 7.737E-08 |
| ASHGA5P052789 | protein_coding | NM_006372 | SYNCRIP | 2.5875952 | 0.00002866 |
| ASHGA5P009062 | protein_coding | NM_201436 | H2AFV | 2.5847609 | 1.022E-08 |
| ASHGA5P028043 | protein_coding | NM_031157 | HNRNPA1 | 2.5774648 | 0.000004355 |
| ASHGA5P017698 | protein_coding | NM_001010931 | HGF | 2.5773411 | 0.00004954 |
| ASHGA5P006359 | protein_coding | NM_147190 | CERS5 | 2.5751716 | 0.00003803 |
| ASHGA5P012594 | protein_coding | NM_006406 | PRDX4 | 2.5732367 | 1.945E-11 |
| ASHGA5P039578 | protein_coding | NM_001237 | CCNA2 | 2.5710044 | 5.354E-07 |
| ASHGA5P018759 | protein_coding | NM_030755 | TMX1 | 2.5702444 | 0.0020786 |
| ASHGA5P050930 | protein_coding | NM_013248 | NXT1 | 2.5686441 | 0.00033 |
| ASHGA5P043746 | protein_coding | NM_003094 | SNRPE | 2.5623579 | 5.853E-08 |
| ASHGA5P001989 | protein_coding | NM_024057 | NUP37 | 2.5562853 | 0.000003065 |
| ASHGA5P005602 | protein_coding | NM_018659 | CYTL1 | 2.5552215 | 0.0116801 |
| ASHGA5P009392 | protein_coding | NM_001002292 | WLS | 2.5544318 | 0.00009213 |
| ASHGA5P004792 | protein_coding | NM_006213 | PHKG1 | 2.5543052 | 0.0000776 |
| ASHGA5P055184 | protein_coding | NM_002105 | H2AFX | 2.5452987 | 1.289E-07 |
| ASHGA5P002638 | protein_coding | NM_005131 | THOC1 | 2.5447933 | 0.0064303 |
| ASHGA5P007249 | protein_coding | NM_001311 | CRIP1 | 2.5445172 | 8.702E-07 |
| ASHGA5P050434 | protein_coding | NM_002709 | PPP1CB | 2.5413204 | 0.00005697 |
| ASHGA5P051045 | protein_coding | NM_016045 | SLMO2 | 2.5410463 | 3.071E-08 |
| ASHGA5P055545 | protein_coding | NM_024685 | BBS10 | 2.5408192 | 2.243E-07 |
| ASHGA5P054540 | protein_coding | NM_020992 | PDLIM1 | 2.539196 | 0.000005015 |
| ASHGA5P001926 | protein_coding | NM_001219 | CALU | 2.5378661 | 0.0063278 |
| ASHGA5P002155 | protein_coding | NM_001142350 | THOC6 | 2.5332765 | 0.000001266 |
| ASHGA5P003057 | protein_coding | NM_000055 | BCHE | 2.5315118 | 0.0153726 |
| ASHGA5P004318 | protein_coding | ENST00000289902 | FCER1G | 2.5297177 | 0.00002594 |
| ASHGA5P055113 | protein_coding | NM_002427 | MMP13 | 2.5282428 | 0.0032546 |
| ASHGA5P007152 | protein_coding | NM_182566 | VMO1 | 2.5257578 | 0.0024118 |
| ASHGA5P004689 | protein_coding | NM_022776 | OSBPL11 | 2.5218781 | 0.00002738 |
| ASHGA5P006658 | protein_coding | NM_181889 | UBE2D3 | 2.5218458 | 0.0004572 |
| ASHGA5P004147 | protein_coding | NM_152565 | ATP6V0D2 | 2.5190195 | 0.000145 |
| ASHGA5P006612 | protein_coding | ENST00000321149 | DYX1C1 | 2.5169561 | 0.00009005 |
| ASHGA5P033169 | protein_coding | ENST00000269025 | LRRC46 | 2.5152954 | 8.301E-08 |
| ASHGA5P000749 | protein_coding | NM_001031849 | MASP1 | 2.514566 | 0.00001901 |
| ASHGA5P001661 | protein_coding | NM_025146 | NAA50 | 2.5122842 | 0.000002328 |
| ASHGA5P013672 | protein_coding | NM_014584 | ERO1L | 2.5081756 | 0.0165242 |
| ASHGA5P017772 | protein_coding | NM_018407 | LAPTM4B | 2.5077476 | 0.0168918 |
| ASHGA5P003882 | protein_coding | NM_144765 | MPZL2 | 2.5074901 | 0.0001713 |
| ASHGA5P050086 | protein_coding | NM_001130823 | DNMT1 | 2.5068421 | 0.0001724 |
| ASHGA5P011970 | protein_coding | NM_007268 | VSIG4 | 2.5062139 | 0.0022601 |
| ASHGA5P043939 | protein_coding | NM_024926 | TTC26 | 2.5047921 | 0.00001858 |
| ASHGA5P012789 | protein_coding | NM_022875 | SMN2 | 2.5031435 | 0.0001142 |
| ASHGA5P004575 | protein_coding | NM_001463 | FRZB | 2.5029522 | 0.000134 |
| ASHGA5P017848 | protein_coding | NM_033293 | CASP1 | 2.501956 | 0.000002759 |
| ASHGA5P042293 | protein_coding | ENST00000360408 | HIST1H3E | 2.4998351 | 5.953E-07 |
| ASHGA5P053904 | protein_coding | NM_005044 | PRKX | 2.499112 | 0.00006382 |
| ASHGA5P055802 | protein_coding | NM_018204 | CKAP2 | 2.495596 | 8.053E-09 |
| ASHGA5P002563 | protein_coding | NM_002210 | ITGAV | 2.4938839 | 8.244E-07 |
| ASHGA5P012102 | protein_coding | NM_025259 | MSH5 | 2.4937517 | 7.557E-07 |
| ASHGA5P000845 | protein_coding | NM_002286 | LAG3 | 2.4934149 | 0.000008308 |
| ASHGA5P011173 | protein_coding | NM_004000 | CHI3L2 | 2.4906423 | 0.0018559 |
| ASHGA5P007251 | protein_coding | NM_001572 | IRF7 | 2.4903693 | 7.694E-10 |
| ASHGA5P005035 | protein_coding | NM_007006 | NUDT21 | 2.4895504 | 1.11E-11 |
| ASHGA5P038473 | protein_coding | NM_013363 | PCOLCE2 | 2.4848187 | 0.0137303 |
| ASHGA5P034754 | protein_coding | ENST00000391785 | ZNF701 | 2.4835702 | 5.856E-07 |
| ASHGA5P007690 | protein_coding | NM_001809 | CENPA | 2.4789943 | 0.00001793 |
| ASHGA5P004163 | protein_coding | NM_018186 | C1orf112 | 2.4765319 | 2.629E-10 |
| ASHGA5P053248 | protein_coding | NM_181747 | ORC5 | 2.4759738 | 2.09E-08 |
| ASHGA5P030483 | protein_coding | NM_016359 | NUSAP1 | 2.4748681 | 0.0002573 |
| ASHGA5P001210 | protein_coding | NM_003941 | WASL | 2.4732727 | 2.235E-08 |
| ASHGA5P010680 | protein_coding | NM_014184 | CNIH4 | 2.4683749 | 9.027E-14 |
| ASHGA5P037992 | protein_coding | NM_181773 | APOBEC3H | 2.4681018 | 0.0003997 |
| ASHGA5P010546 | protein_coding | ENST00000361919 | PRC1 | 2.4675189 | 0.0156614 |
| ASHGA5P041097 | protein_coding | NM_006144 | GZMA | 2.4674685 | 0.0001374 |
| ASHGA5P053075 | protein_coding | NM_201563 | FCGR2C | 2.4673873 | 0.000001467 |
| ASHGA5P010695 | protein_coding | ENST00000366932 | RRP15 | 2.4630805 | 0.00001454 |
| ASHGA5P003046 | protein_coding | ENST00000264265 | LXN | 2.4595766 | 0.00001434 |
| ASHGA5P005054 | protein_coding | NM_000732 | CD3D | 2.4525864 | 0.0002658 |
| ASHGA5P056062 | protein_coding | NM_013943 | CLIC4 | 2.4521623 | 0.00007332 |
| ASHGA5P029663 | protein_coding | NM_006399 | BATF | 2.4516775 | 0.0006368 |
| ASHGA5P011185 | protein_coding | NM_006496 | GNAI3 | 2.3556681 | 0.0143837 |
| ASHGA5P009128 | protein_coding | NM_001135 | ACAN | 2.3552944 | 0.0007373 |
| ASHGA5P007779 | protein_coding | NM_001135095 | FNDC3B | 2.3544491 | 0.0013508 |
| ASHGA5P003400 | protein_coding | NM_017661 | ZNF280D | 2.353199 | 2.783E-10 |
| ASHGA5P007150 | protein_coding | ENST00000328735 | BACE2 | 2.3512947 | 0.0004523 |
| ASHGA5P005621 | protein_coding | NM_182763 | MCL1 | 2.3505792 | 0.00001183 |
| ASHGA5P027101 | protein_coding | NM_024829 | PLBD1 | 2.3484712 | 0.0011216 |
| ASHGA5P014048 | protein_coding | NM_003965 | CCRL2 | 2.3473164 | 9.525E-07 |
| ASHGA5P011126 | protein_coding | NM_001779 | CD58 | 2.3450213 | 0.000001465 |
| ASHGA5P006957 | protein_coding | NM_024339 | THOC6 | 2.3449833 | 0.000001141 |
| ASHGA5P004641 | protein_coding | NM_005531 | IFI16 | 2.3432242 | 0.034662 |
| ASHGA5P041712 | protein_coding | ENST00000305910 | HIST1H3G | 2.3426971 | 0.0002674 |
| ASHGA5P008682 | protein_coding | NM_001127181 | CENPL | 2.3420512 | 0.000001568 |
| ASHGA5P006636 | protein_coding | NM_022754 | SFXN1 | 2.3417605 | 0.000001202 |
| ASHGA5P052741 | protein_coding | NM_001025370 | VEGFA | 2.3395188 | 0.0039781 |
| ASHGA5P010633 | protein_coding | NM_003679 | KMO | 2.3374997 | 0.001389 |
| ASHGA5P051425 | protein_coding | NM_001012413 | SGOL1 | 2.3374848 | 0.000001509 |
| ASHGA5P012791 | protein_coding | NM_017411 | SMN2 | 2.3357226 | 0.0001222 |
| ASHGA5P016830 | protein_coding | NM_032930 | C11orf70 | 2.3324905 | 0.0038084 |
| ASHGA5P012589 | protein_coding | NM_001037171 | ACOT9 | 2.3315835 | 0.0024485 |
| ASHGA5P002991 | protein_coding | ENST00000263904 | STAM2 | 2.3312562 | 0.00009902 |
| ASHGA5P010415 | protein_coding | NM_015696 | GPX7 | 2.3262586 | 5.847E-07 |
| ASHGA5P004254 | protein_coding | NM_001018006 | TPM1 | 2.320379 | 0.00001337 |
| ASHGA5P001673 | protein_coding | NM_003467 | CXCR4 | 2.3202743 | 0.0067441 |
| ASHGA5P040921 | protein_coding | NM_005110 | GFPT2 | 2.3164376 | 0.0233058 |
| ASHGA5P050621 | protein_coding | NM_152515 | CKAP2L | 2.3124204 | 0.00001295 |
| ASHGA5P008907 | protein_coding | ENST00000348518 | DYX1C1 | 2.302369 | 0.00007173 |
| ASHGA5P010500 | protein_coding | NM_022037 | TIA1 | 2.2933873 | 5.62E-08 |
| ASHGA5P001520 | protein_coding | NM_012476 | VAX2 | 2.2897028 | 0.000005932 |
| ASHGA5P048736 | protein_coding | NM_016530 | RAB8B | 2.2886763 | 0.0003062 |
| ASHGA5P008689 | protein_coding | NM_177974 | CASC4 | 2.2880365 | 0.000001381 |
| ASHGA5P009964 | protein_coding | NM_031266 | HNRNPAB | 2.2872926 | 0.000004112 |
| ASHGA5P050775 | protein_coding | NM_201264 | NRP2 | 2.2865154 | 2.509E-07 |
| ASHGA5P012990 | protein_coding | ENST00000382213 | IL32 | 2.2836646 | 0.0076535 |
| ASHGA5P051822 | protein_coding | NM_134470 | IL1RAP | 2.2836217 | 0.00005598 |
| ASHGA5P006104 | protein_coding | NM_001130090 | LRRC48 | 2.2832604 | 0.0003648 |
| ASHGA5P001705 | protein_coding | NM_014182 | ORMDL2 | 2.2830173 | 0.0048777 |
| ASHGA5P029718 | protein_coding | NM_001098621 | TMEM251 | 2.2812572 | 0.000007068 |
| ASHGA5P036974 | protein_coding | NM_018474 | PLK1S1 | 2.2807344 | 0.000001985 |
| ASHGA5P038876 | protein_coding | NM_174907 | PPP4R2 | 2.2787616 | 3.243E-09 |
| ASHGA5P013661 | protein_coding | NM_001012456 | SEC61G | 2.277552 | 0.00001389 |
| ASHGA5P033141 | protein_coding | NM_001466 | FZD2 | 2.2771269 | 0.00003114 |
| ASHGA5P012134 | protein_coding | NM_006573 | TNFSF13B | 2.2765949 | 0.00009487 |
| ASHGA5P002594 | protein_coding | NM_013269 | CLEC2D | 2.275092 | 7.143E-07 |
| ASHGA5P019342 | protein_coding | NM_001170752 | SRPX | 2.2688505 | 0.0001465 |
| ASHGA5P040742 | protein_coding | ENST00000401743 | CD14 | 2.2659869 | 0.0085299 |
| ASHGA5P043942 | protein_coding | ENST00000297534 | C7orf55 | 2.2646551 | 1.752E-07 |
| ASHGA5P055109 | protein_coding | NM_182962 | BIRC3 | 2.2616744 | 0.0019229 |
| ASHGA5P039800 | protein_coding | NM_005980 | S100P | 2.2609427 | 0.0020901 |
| ASHGA5P003906 | protein_coding | NM_001145426 | CSDA | 2.258725 | 0.0456994 |
| ASHGA5P010946 | protein_coding | NM_006912 | RIT1 | 2.2586453 | 0.0043156 |
| ASHGA5P001718 | protein_coding | NM_004688 | NMI | 2.257476 | 0.00004091 |
| ASHGA5P012263 | protein_coding | NM_152420 | C9orf41 | 2.2536775 | 0.005593 |
| ASHGA5P006620 | protein_coding | NM_015344 | LEPROTL1 | 2.2535697 | 2.707E-08 |
| ASHGA5P031803 | protein_coding | NM_005952 | MT1X | 2.2481427 | 0.00009088 |
| ASHGA5P045922 | protein_coding | NM_000607 | ORM1 | 2.2475094 | 0.0008035 |
| ASHGA5P008456 | protein_coding | NM_153358 | ZNF791 | 2.2468213 | 3.531E-07 |
| ASHGA5P055334 | protein_coding | NM_021821 | MRPS35 | 2.2459552 | 0.0003603 |
| ASHGA5P019485 | protein_coding | NM_003816 | ADAM9 | 2.243334 | 0.0066388 |
| ASHGA5P017048 | protein_coding | NM_001100595 | SKA2 | 2.24305 | 7.138E-11 |
| ASHGA5P019797 | protein_coding | NM_021928 | SPCS3 | 2.2427914 | 0.00001239 |
| ASHGA5P001514 | protein_coding | NM_005813 | PRKD3 | 2.2407513 | 3.911E-07 |
| ASHGA5P040879 | protein_coding | NM_152277 | UBTD2 | 2.2389076 | 0.0035107 |
| ASHGA5P002405 | protein_coding | NM_001098525 | CKAP2 | 2.2389074 | 0.0049513 |
| ASHGA5P046986 | protein_coding | NM_144590 | ANKRD22 | 2.2377246 | 0.0252942 |
| ASHGA5P048746 | protein_coding | NM_152304 | RAB42 | 2.2362942 | 0.00003336 |
| ASHGA5P017631 | protein_coding | NM_001145277 | NECAP2 | 2.2346349 | 0.0005252 |
| ASHGA5P008826 | protein_coding | NM_000855 | GUCY1A2 | 2.2342943 | 0.0022901 |
| ASHGA5P006074 | protein_coding | ENST00000335934 | TP53I3 | 2.2299332 | 0.0022846 |
| ASHGA5P014734 | protein_coding | NM_001100431 | VSIG4 | 2.2295534 | 0.0150751 |
| ASHGA5P004503 | protein_coding | NM_001105578 | SYCE2 | 2.2292462 | 0.004677 |
| ASHGA5P002728 | protein_coding | NM_031216 | SEH1L | 2.2248463 | 0.0020154 |
| ASHGA5P005047 | protein_coding | NM_001849 | COL6A2 | 2.2243892 | 0.0027908 |
| ASHGA5P016457 | protein_coding | NM_001145645 | TNFSF13B | 2.2236792 | 0.0000957 |
| ASHGA5P010516 | protein_coding | NM_002589 | PCDH7 | 2.2221313 | 0.00003222 |
| ASHGA5P055485 | protein_coding | NM_000946 | PRIM1 | 2.2168673 | 0.000001144 |
| ASHGA5P053091 | protein_coding | ENST00000335503 | CCT6A | 2.2144727 | 0.0113585 |
| ASHGA5P014548 | protein_coding | NM_012322 | LSM5 | 2.2143672 | 0.000005139 |
| ASHGA5P053721 | protein_coding | NM_206948 | TRPM3 | 2.2115345 | 0.0035422 |
| ASHGA5P004850 | protein_coding | ENST00000373758 | REEP3 | 2.2107524 | 1.748E-09 |
| ASHGA5P007641 | protein_coding | NM_001101376 | FAM183A | 2.2095627 | 0.0023724 |
| ASHGA5P007906 | protein_coding | NM_145212 | MRPL30 | 2.2091274 | 1.285E-07 |
| ASHGA5P004810 | protein_coding | NM_015420 | DCAF13 | 2.2090482 | 2.223E-07 |
| ASHGA5P053664 | protein_coding | NM_052966 | FAM129A | 2.2054605 | 0.0016985 |
| ASHGA5P008727 | protein_coding | NM_181762 | UBE2A | 2.2027305 | 1.601E-10 |
| ASHGA5P053924 | protein_coding | NM_014176 | UBE2T | 2.2025903 | 0.000006867 |
| ASHGA5P011340 | protein_coding | NM_001170704 | MBNL3 | 2.2017956 | 9.115E-08 |
| ASHGA5P050280 | protein_coding | NM_199249 | C19orf48 | 2.1999996 | 2.695E-07 |
| ASHGA5P050734 | protein_coding | NM_006287 | TFPI | 2.1977822 | 0.00004671 |
| ASHGA5P044663 | protein_coding | NM_001007090 | C8orf48 | 2.1962843 | 0.000006687 |
| ASHGA5P056054 | protein_coding | NM_004450 | ERH | 2.1961318 | 0.0058935 |
| ASHGA5P004277 | protein_coding | NM_022061 | MRPL17 | 2.1942251 | 7.171E-07 |
| ASHGA5P007373 | protein_coding | NM_001614 | ACTG1 | 2.1930985 | 0.0444124 |
| ASHGA5P009981 | protein_coding | NM_201441 | TEAD4 | 2.1913877 | 0.0015967 |
| ASHGA5P007361 | protein_coding | NM_003364 | UPP1 | 2.1912478 | 0.00009475 |
| ASHGA5P005335 | protein_coding | NM_001130158 | MYO1B | 2.1886911 | 0.0100885 |
| ASHGA5P045495 | protein_coding | NM_052844 | WDR34 | 2.1880835 | 1.328E-07 |
| ASHGA5P004706 | protein_coding | NM_002106 | H2AFZ | 2.187697 | 0.0165822 |
| ASHGA5P020064 | protein_coding | NM_001130929 | C7orf73 | 2.1871545 | 0.000001727 |
| ASHGA5P001088 | protein_coding | NM_018163 | DNAJC17 | 2.1852885 | 6.924E-07 |
| ASHGA5P010471 | protein_coding | ENST00000361570 | CDKN2A | 2.1834624 | 0.0048928 |
| ASHGA5P012660 | protein_coding | NM_001145549 | TXNDC5 | 2.1833946 | 0.0078383 |
| ASHGA5P010377 | protein_coding | NM_001005413 | ZWINT | 2.1821662 | 0.0020245 |
| ASHGA5P026761 | protein_coding | ENST00000393127 | GRIA4 | 2.1816482 | 0.00005563 |
| ASHGA5P039580 | protein_coding | ENST00000334179 | UBL4B | 2.1816317 | 0.00001269 |
| ASHGA5P005640 | protein_coding | NM_012412 | H2AFV | 2.180315 | 8.907E-11 |
| ASHGA5P003406 | protein_coding | NM_001018100 | MYZAP | 2.1788442 | 0.0006701 |
| ASHGA5P001466 | protein_coding | NM_000373 | UMPS | 2.1755214 | 0.0029366 |
| ASHGA5P012370 | protein_coding | NM_001082537 | TCTN1 | 2.1736615 | 1.831E-10 |
| ASHGA5P050081 | protein_coding | NM_152289 | ZNF561 | 2.1727579 | 0.0042352 |
| ASHGA5P007258 | protein_coding | ENST00000330333 | BACE2 | 2.1722988 | 0.0004851 |
| ASHGA5P043344 | protein_coding | NM_181775 | PLXNA4 | 2.1712252 | 0.00086 |
| ASHGA5P016216 | protein_coding | ENST00000428041 | ARHGAP11B | 2.1712178 | 0.000716 |
| ASHGA5P050795 | protein_coding | NM_018000 | MREG | 2.1710354 | 0.0125387 |
| ASHGA5P053305 | protein_coding | NM_001098786 | HILPDA | 2.1708902 | 0.0021957 |
| ASHGA5P016968 | protein_coding | NM_003014 | SFRP4 | 2.1691987 | 0.0222197 |
| ASHGA5P007014 | protein_coding | NM_153686 | LCORL | 2.1689412 | 1.155E-08 |
| ASHGA5P004537 | protein_coding | NM_198545 | C1orf187 | 2.1683752 | 0.0001009 |
| ASHGA5P009292 | protein_coding | NM_001144941 | VMO1 | 2.1667545 | 0.0005698 |
| ASHGA5P051608 | protein_coding | ENST00000383694 | FILIP1L | 2.1648355 | 0.00001441 |
| ASHGA5P011984 | protein_coding | NM_002800 | PSMB9 | 2.1642378 | 1.758E-07 |
| ASHGA5P014543 | protein_coding | NM_001130710 | LSM5 | 2.1622256 | 0.00001097 |
| ASHGA5P052640 | protein_coding | NM_005572 | LMNA | 2.1622001 | 0.000001422 |
| ASHGA5P003748 | protein_coding | NM_000596 | IGFBP1 | 2.1611226 | 0.0063077 |
| ASHGA5P002384 | protein_coding | NM_030769 | NPL | 2.1601877 | 0.0000295 |
| ASHGA5P043326 | protein_coding | NM_199349 | KCP | 2.1586689 | 0.0003453 |
| ASHGA5P002681 | protein_coding | NM_032802 | SPPL2A | 2.158451 | 5.507E-09 |
| ASHGA5P041940 | protein_coding | NM_016021 | UBE2J1 | 2.1576324 | 0.0021334 |
| ASHGA5P007747 | protein_coding | NM_001033583 | ACOT9 | 2.1568357 | 0.0028969 |
| ASHGA5P033954 | protein_coding | NM_016199 | LSM7 | 2.1564634 | 7.618E-10 |
| ASHGA5P004616 | protein_coding | NM_173654 | EOGT | 2.1559831 | 0.0001193 |
| ASHGA5P044496 | protein_coding | NM_024094 | DSCC1 | 2.154779 | 0.0020827 |
| ASHGA5P003803 | protein_coding | NM_032171 | CEP78 | 2.1543596 | 0.0063109 |
| ASHGA5P034748 | protein_coding | NM_144684 | ZNF480 | 2.1527798 | 2.195E-07 |
| ASHGA5P004995 | protein_coding | NM_018193 | FANCI | 2.1526251 | 0.0027058 |
| ASHGA5P049454 | protein_coding | NM_032731 | TXNDC17 | 2.1523676 | 1.032E-09 |
| ASHGA5P046737 | protein_coding | NM_145177 | DHRSX | 2.1523198 | 1.881E-07 |
| ASHGA5P013824 | protein_coding | NM_001012633 | IL32 | 2.1516801 | 0.000749 |
| ASHGA5P022047 | protein_coding | NM_139201 | GIT2 | 2.1510463 | 1.277E-09 |
| ASHGA5P011326 | protein_coding | NM_003902 | FUBP1 | 2.1502849 | 0.000146 |
| ASHGA5P003410 | protein_coding | NM_004049 | BCL2A1 | 2.1497592 | 0.000002737 |
| ASHGA5P009313 | protein_coding | NM_019554 | S100A4 | 2.1497193 | 0.000003086 |
| ASHGA5P027099 | protein_coding | NM_005274 | GNG5 | 2.1484999 | 9.696E-10 |
| ASHGA5P029595 | protein_coding | ENST00000335674 | LCE3A | 2.1470348 | 0.0001685 |
| ASHGA5P052663 | protein_coding | ENST00000375650 | HSPA1B | 2.1463917 | 0.0006581 |
| ASHGA5P008061 | protein_coding | NM_012223 | MYO1B | 2.1455252 | 0.0397056 |
| ASHGA5P002396 | protein_coding | NM_004846 | EIF4E2 | 2.1453251 | 2.088E-12 |
| ASHGA5P005928 | protein_coding | ENST00000311469 | COQ2 | 2.1420803 | 2.108E-08 |
| ASHGA5P005436 | protein_coding | NM_012145 | DTYMK | 2.1419312 | 0.0025951 |
| ASHGA5P006917 | protein_coding | NM_004221 | IL32 | 2.1412785 | 0.0126844 |
| ASHGA5P013917 | protein_coding | NM_000245 | MET | 2.1398752 | 0.011761 |
| ASHGA5P000686 | protein_coding | ENST00000054666 | VAMP3 | 2.138997 | 0.00006571 |
| ASHGA5P014326 | protein_coding | NM_004956 | ETV1 | 2.1377303 | 0.0001342 |
| ASHGA5P022819 | protein_coding | NM_183234 | RAB27A | 2.1370087 | 0.0013896 |
| ASHGA5P054088 | protein_coding | NM_018301 | RBM41 | 2.133073 | 0.0009232 |
| ASHGA5P003481 | protein_coding | NM_005637 | SS18 | 2.1326602 | 0.0057914 |
| ASHGA5P042312 | protein_coding | ENST00000359465 | HIST1H2BM | 2.1324321 | 0.00003011 |
| ASHGA5P047908 | protein_coding | ENST00000315204 | STK33 | 2.1306764 | 0.000001783 |
| ASHGA5P007033 | protein_coding | NM_002894 | RBBP8 | 2.1291137 | 0.0351073 |
| ASHGA5P048713 | protein_coding | ENST00000260453 | MNS1 | 2.1283667 | 0.0009213 |
| ASHGA5P004963 | protein_coding | NM_014358 | CLEC4E | 2.127951 | 0.0056469 |
| ASHGA5P036159 | protein_coding | NM_013310 | C2orf27A | 2.1278347 | 3.392E-07 |
| ASHGA5P008113 | protein_coding | NM_001184794 | PARD3 | 2.1273211 | 3.815E-08 |
| ASHGA5P054797 | protein_coding | NM_005898 | CAPRIN1 | 2.1262128 | 9.856E-14 |
| ASHGA5P055154 | protein_coding | NM_016090 | RBM7 | 2.123666 | 0.0002454 |
| ASHGA5P042592 | protein_coding | ENST00000368605 | FAM26F | 2.1230162 | 0.0001274 |
| ASHGA5P010850 | protein_coding | NM_012474 | UCK2 | 2.1226431 | 0.0017103 |
| ASHGA5P003719 | protein_coding | ENST00000274599 | ZNF300 | 2.1206886 | 0.00006353 |
| ASHGA5P011320 | protein_coding | NM_006417 | IFI44 | 2.120629 | 0.0001192 |
| ASHGA5P018427 | protein_coding | NM_001010933 | HGF | 2.1190912 | 0.0006716 |
| ASHGA5P001770 | protein_coding | NM_001134709 | DEK | 2.1185733 | 0.0162668 |
| ASHGA5P029042 | protein_coding | NM_033423 | GZMH | 2.1162016 | 0.0028216 |
| ASHGA5P035952 | protein_coding | NM_000189 | HK2 | 2.1160888 | 0.0049781 |
| ASHGA5P002353 | protein_coding | NM_001780 | CD63 | 2.1158719 | 0.015011 |
| ASHGA5P054006 | protein_coding | ENST00000425133 | FAM104B | 2.1156621 | 7.175E-13 |
| ASHGA5P032863 | protein_coding | NM_006041 | HS3ST3B1 | 2.1155782 | 0.0005893 |
| ASHGA5P033602 | protein_coding | ENST00000425639 | SKOR2 | 2.1143321 | 0.0181793 |
| ASHGA5P013126 | protein_coding | NM_015000 | STK38L | 2.1125928 | 0.0029568 |
| ASHGA5P001768 | protein_coding | NM_003144 | SSR1 | 2.1091048 | 0.00001449 |
| ASHGA5P003030 | protein_coding | NM_003937 | KYNU | 2.1089073 | 0.0032151 |
| ASHGA5P006684 | protein_coding | NM_001031710 | KLHL7 | 2.1067903 | 0.0033633 |
| ASHGA5P013715 | protein_coding | NM_018221 | MOB1A | 2.1063513 | 0.0001196 |
| ASHGA5P010893 | protein_coding | ENST00000368046 | CD48 | 2.1050102 | 2.814E-07 |
| ASHGA5P052780 | protein_coding | NM_003974 | DOK2 | 2.1019256 | 0.001107 |
| ASHGA5P049311 | protein_coding | NM_001126102 | HP | 2.1015746 | 0.0144983 |
| ASHGA5P009377 | protein_coding | NM_021644 | HNRNPH3 | 2.0999209 | 0.0013321 |
| ASHGA5P010580 | protein_coding | NM_016326 | CKLF | 2.0984912 | 8.465E-12 |
| ASHGA5P014814 | protein_coding | NM_001142403 | CD164 | 2.0983979 | 0.0315162 |
| ASHGA5P000783 | protein_coding | NM_013360 | ZNF222 | 2.0966199 | 0.0001873 |
| ASHGA5P002770 | protein_coding | NM_024911 | WLS | 2.0956824 | 0.000086 |
| ASHGA5P054310 | protein_coding | NM_152490 | B3GALNT2 | 2.0949598 | 0.000001736 |
| ASHGA5P004768 | protein_coding | NM_173674 | DCBLD1 | 2.0948559 | 0.000007306 |
| ASHGA5P050709 | protein_coding | ENST00000313173 | HOXD8 | 2.092626 | 0.001218 |
| ASHGA5P012701 | protein_coding | ENST00000396855 | TRIM5 | 2.0894306 | 0.0002153 |
| ASHGA5P048233 | protein_coding | NM_170739 | MRPL11 | 2.0887177 | 2.434E-07 |
| ASHGA5P036246 | protein_coding | NM_016653 | ZAK | 2.0878475 | 0.012092 |
| ASHGA5P002595 | protein_coding | NM_001004419 | CLEC2D | 2.0872713 | 0.0014649 |
| ASHGA5P026744 | protein_coding | NM_032021 | TMEM133 | 2.0862358 | 0.000007256 |
| ASHGA5P016659 | protein_coding | NM_020186 | ACN9 | 2.0862322 | 0.0141175 |
| ASHGA5P026359 | protein_coding | NM_006172 | NPPA | 2.0855796 | 0.0179307 |
| ASHGA5P051102 | protein_coding | NM_001162495 | C21orf62 | 2.0853966 | 0.0460768 |
| ASHGA5P008048 | protein_coding | NM_001031692 | LRRC17 | 2.0842664 | 0.0069301 |
| ASHGA5P003839 | protein_coding | ENST00000278200 | IMMP1L | 2.0797227 | 0.00001464 |
| ASHGA5P055396 | protein_coding | NM_213566 | DFFA | 2.0786001 | 0.000007444 |
| ASHGA5P051784 | protein_coding | NM_178042 | ACTL6A | 2.0782916 | 0.0398695 |
| ASHGA5P051467 | protein_coding | NM_001904 | CTNNB1 | 2.0782177 | 0.0087839 |
| ASHGA5P002344 | protein_coding | NM_181837 | ORC3 | 2.0781934 | 2.177E-08 |
| ASHGA5P051701 | protein_coding | NM_021105 | PLSCR1 | 2.0771869 | 0.0000677 |
| ASHGA5P007022 | protein_coding | NM_001134832 | AHI1 | 2.0739997 | 0.000003542 |
| ASHGA5P018462 | protein_coding | NM_001191009 | SRSF10 | 2.0733205 | 7.739E-10 |
| ASHGA5P005514 | protein_coding | NM_001565 | CXCL10 | 2.0724632 | 0.0332284 |
| ASHGA5P002248 | protein_coding | NM_001442 | FABP4 | 2.0694202 | 0.0005423 |
| ASHGA5P052250 | protein_coding | NM_001008397 | GPX8 | 2.0685054 | 0.015439 |
| ASHGA5P006599 | protein_coding | NM_018048 | MAGOHB | 2.067487 | 7.367E-09 |
| ASHGA5P005834 | protein_coding | NM_003747 | TNKS | 2.0674507 | 0.0007279 |
| ASHGA5P005965 | protein_coding | NM_182835 | SCFD1 | 2.0669819 | 0.0145307 |
| ASHGA5P001626 | protein_coding | NM_022716 | PRRX1 | 2.0632917 | 2.152E-07 |
| ASHGA5P044441 | protein_coding | NM_198401 | ANKRD46 | 2.0620992 | 0.0007878 |
| ASHGA5P004952 | protein_coding | NM_018455 | CENPN | 2.060833 | 0.0004209 |
| ASHGA5P008439 | protein_coding | NM_001144923 | TTC26 | 2.0598901 | 0.0063616 |
| ASHGA5P050327 | protein_coding | NM_207014 | WDR78 | 2.0598011 | 0.000001006 |
| ASHGA5P001363 | protein_coding | NM_002046 | GAPDH | 2.0566068 | 0.0368083 |
| ASHGA5P005893 | protein_coding | ENST00000311127 | HEG1 | 2.0563147 | 0.0007412 |
| ASHGA5P056097 | protein_coding | NM_003608 | GPR65 | 2.0553128 | 0.01079 |
| ASHGA5P004518 | protein_coding | NM_001142500 | FLYWCH2 | 2.0550864 | 0.0000276 |
| ASHGA5P054749 | protein_coding | NM_138421 | SAAL1 | 2.0534369 | 0.000001194 |
| ASHGA5P020808 | protein_coding | NM_080805 | COL13A1 | 2.0505935 | 0.0016195 |
| ASHGA5P016870 | protein_coding | NM_001142288 | EMC8 | 2.0488534 | 6.005E-09 |
| ASHGA5P046672 | protein_coding | ENST00000340077 | PARD3 | 2.0485247 | 1.753E-07 |
| ASHGA5P003344 | protein_coding | NM_006851 | GLIPR1 | 2.0483584 | 0.000003103 |
| ASHGA5P015071 | protein_coding | NM_001144940 | VMO1 | 2.0481085 | 0.0009102 |
| ASHGA5P052605 | protein_coding | NM_080596 | HIST1H2AH | 2.0475994 | 0.0049967 |
| ASHGA5P010270 | protein_coding | ENST00000360465 | ZNF528 | 2.0438434 | 0.00000333 |
| ASHGA5P055139 | protein_coding | NM_138789 | PIH1D2 | 2.0435464 | 1.937E-09 |
| ASHGA5P010790 | protein_coding | NM_007212 | RNF2 | 2.0420211 | 0.0018678 |
| ASHGA5P003482 | protein_coding | NM_001007559 | SS18 | 2.0415917 | 0.0012116 |
| ASHGA5P049250 | protein_coding | NM_206999 | CNOT1 | 2.0404248 | 0.000000213 |
| ASHGA5P010795 | protein_coding | NM_002928 | RGS16 | 2.0403925 | 0.0001073 |
| ASHGA5P052506 | protein_coding | NM_003945 | ATP6V0E1 | 2.0395626 | 0.0025408 |
| ASHGA5P052451 | protein_coding | NM_001112724 | STK32A | 2.0387208 | 0.00001726 |
| ASHGA5P004688 | protein_coding | ENST00000296218 | DNALI1 | 2.0385387 | 0.00001084 |
| ASHGA5P001305 | protein_coding | NM_001165416 | LDHA | 2.038311 | 0.009244 |
| ASHGA5P051903 | protein_coding | NM_031950 | FGFBP2 | 2.0382267 | 0.0058078 |
| ASHGA5P043695 | protein_coding | NM_001013746 | ZNF107 | 2.037362 | 0.00004586 |
| ASHGA5P036304 | protein_coding | NM_000090 | COL3A1 | 2.0360237 | 0.0311824 |
| ASHGA5P000942 | protein_coding | NM_032632 | PAPOLA | 2.0351349 | 1.614E-07 |
| ASHGA5P051605 | protein_coding | NM_005105 | RBM8A | 2.034717 | 0.0002966 |
| ASHGA5P011374 | protein_coding | NM_001172420 | EFHC1 | 2.0346192 | 0.0243312 |
| ASHGA5P002280 | protein_coding | NM_080650 | ATPBD4 | 2.0342323 | 3.839E-07 |
| ASHGA5P050456 | protein_coding | NM_001101330 | LOC728819 | 2.033191 | 0.00001519 |
| ASHGA5P052403 | protein_coding | NM_032151 | PCBD2 | 2.032536 | 4.753E-07 |
| ASHGA5P010758 | protein_coding | NM_024573 | C6orf211 | 2.0316109 | 0.0001289 |
| ASHGA5P031146 | protein_coding | NM_024745 | SHCBP1 | 2.031361 | 0.0055938 |
| ASHGA5P006551 | protein_coding | NM_001178090 | ZNF454 | 2.03007 | 0.000009231 |
| ASHGA5P010551 | protein_coding | NM_018122 | DARS2 | 2.0299258 | 1.589E-09 |
| ASHGA5P013829 | protein_coding | NM_006350 | FST | 2.0299187 | 0.0037057 |
| ASHGA5P015070 | protein_coding | NM_178539 | FAM19A2 | -2.0315331 | 0.00001257 |
| ASHGA5P053776 | protein_coding | NM_000035 | ALDOB | -2.0317907 | 0.000005562 |
| ASHGA5P027280 | protein_coding | NM_001145475 | FAM186A | -2.0320894 | 2.505E-08 |
| ASHGA5P040171 | protein_coding | NM_006439 | MAB21L2 | -2.0322492 | 0.000001266 |
| ASHGA5P010916 | protein_coding | NM_001185113 | CD1E | -2.0342091 | 0.0001249 |
| ASHGA5P038917 | protein_coding | NM_020351 | COL8A1 | -2.0366711 | 0.000008207 |
| ASHGA5P017916 | protein_coding | ENST00000447098 | PTCH2 | -2.0387846 | 0.00002714 |
| ASHGA5P055583 | protein_coding | NM_001204081 | ANKS1B | -2.0390668 | 0.0046104 |
| ASHGA5P010548 | protein_coding | NM_080600 | MAG | -2.0393215 | 0.0000698 |
| ASHGA5P013925 | protein_coding | NM_014878 | KIAA0020 | -2.0413269 | 2.641E-08 |
| ASHGA5P008669 | protein_coding | NM_005704 | PTPRU | -2.0432179 | 0.000006384 |
| ASHGA5P007057 | protein_coding | NM_175931 | CBFA2T3 | -2.0451986 | 1.898E-09 |
| ASHGA5P020901 | protein_coding | NM_001100393 | RALYL | -2.0484691 | 6.276E-07 |
| ASHGA5P049794 | protein_coding | NM_001441 | FAAH | -2.0491596 | 4.682E-07 |
| ASHGA5P007419 | protein_coding | NM_001204079 | ANKS1B | -2.0518872 | 0.00002112 |
| ASHGA5P006290 | protein_coding | NM_139343 | BIN1 | -2.0520364 | 1.243E-07 |
| ASHGA5P005316 | protein_coding | NM_130463 | ATP6V1G2 | -2.0538044 | 0.008582 |
| ASHGA5P040506 | protein_coding | ENST00000322348 | GCNT4 | -2.0558755 | 0.00004293 |
| ASHGA5P006270 | protein_coding | NM_014232 | VAMP2 | -2.0567818 | 3.005E-07 |
| ASHGA5P000722 | protein_coding | NM_001160300 | PANX2 | -2.0621806 | 9.35E-09 |
| ASHGA5P012448 | protein_coding | ENST00000430948 | KIAA0319 | -2.0637041 | 0.0008455 |
| ASHGA5P040907 | protein_coding | ENST00000308304 | PROP1 | -2.0645392 | 0.0013996 |
| ASHGA5P055255 | protein_coding | NM_006422 | AKAP3 | -2.0664821 | 1.334E-07 |
| ASHGA5P009654 | protein_coding | NM_001025108 | AFF3 | -2.0673367 | 0.0002273 |
| ASHGA5P005225 | protein_coding | NM_015460 | MYRIP | -2.0674594 | 4.015E-08 |
| ASHGA5P027086 | protein_coding | ENST00000533467 | TAS2R46 | -2.0677394 | 2.129E-07 |
| ASHGA5P014914 | protein_coding | NM_001128602 | RASGRP1 | -2.0677493 | 0.000001724 |
| ASHGA5P048990 | protein_coding | ENST00000307394 | EME2 | -2.0687312 | 0.000001994 |
| ASHGA5P010815 | protein_coding | NM_001170723 | TEX35 | -2.0698362 | 0.000003156 |
| ASHGA5P051007 | protein_coding | NM_014276 | RBPJL | -2.0726731 | 0.00009345 |
| ASHGA5P006517 | protein_coding | NM_018030 | OSBPL1A | -2.0743395 | 0.0003079 |
| ASHGA5P026879 | protein_coding | ENST00000412681 | NRGN | -2.0753746 | 0.00254 |
| ASHGA5P005999 | protein_coding | NM_001134406 | RUNDC3B | -2.0778853 | 0.023925 |
| ASHGA5P054186 | protein_coding | NM_005334 | HCFC1 | -2.0796351 | 5.859E-11 |
| ASHGA5P008717 | protein_coding | NM_139347 | BIN1 | -2.0833281 | 1.734E-07 |
| ASHGA5P007892 | protein_coding | NM_139021 | MAPK15 | -2.091095 | 1.844E-07 |
| ASHGA5P006595 | protein_coding | NM_014905 | GLS | -2.0938347 | 0.001565 |
| ASHGA5P009555 | protein_coding | NM_001130016 | ART3 | -2.0939423 | 0.000001708 |
| ASHGA5P044270 | protein_coding | NM_001146227 | RPS20 | -2.095109 | 7.482E-07 |
| ASHGA5P000623 | protein_coding | NM_006539 | CACNG3 | -2.100043 | 0.000216 |
| ASHGA5P051994 | protein_coding | NM_139076 | FAM175A | -2.1007988 | 2.218E-09 |
| ASHGA5P013076 | protein_coding | NM_015175 | NBEAL2 | -2.1013281 | 0.0002822 |
| ASHGA5P006321 | protein_coding | NM_005070 | SLC4A3 | -2.1014311 | 0.000002038 |
| ASHGA5P030637 | protein_coding | NM_018652 | GOLGA6B | -2.1017583 | 0.000000129 |
| ASHGA5P004948 | protein_coding | NM_016229 | CYB5R2 | -2.1024066 | 0.0016667 |
| ASHGA5P004009 | protein_coding | NM_006887 | ZFP36L2 | -2.105457 | 0.000001019 |
| ASHGA5P021659 | protein_coding | NM_001169107 | FAM21C | -2.1070816 | 0.000001969 |
| ASHGA5P049629 | protein_coding | NM_001001435 | CCL4L1 | -2.109159 | 0.0018307 |
| ASHGA5P010082 | protein_coding | NM_001001323 | ATP2B1 | -2.1096196 | 0.00002493 |
| ASHGA5P006100 | protein_coding | NM_025129 | FUZ | -2.1097141 | 0.0002691 |
| ASHGA5P006396 | protein_coding | ENST00000317991 | GRAMD1A | -2.1098362 | 4.634E-08 |
| ASHGA5P010291 | protein_coding | NM_206860 | TACC2 | -2.1099058 | 0.00000527 |
| ASHGA5P002357 | protein_coding | NM_032130 | FAM186B | -2.1127063 | 0.000001134 |
| ASHGA5P050963 | protein_coding | NM_000557 | GDF5 | -2.1135734 | 0.0001007 |
| ASHGA5P044400 | protein_coding | NM_001242668 | C8orf87 | -2.1160985 | 2.045E-07 |
| ASHGA5P045326 | protein_coding | NM_005014 | OMD | -2.1182923 | 0.0093188 |
| ASHGA5P008523 | protein_coding | NM_001129829 | CACNA1C | -2.1222828 | 0.000001312 |
| ASHGA5P049076 | protein_coding | ENST00000438167 | PLA2G10 | -2.1224805 | 0.00003407 |
| ASHGA5P046522 | protein_coding | NM_005635 | SSX1 | -2.1231586 | 0.00003079 |
| ASHGA5P004649 | protein_coding | NM_001263 | CDS1 | -2.1244685 | 0.0096867 |
| ASHGA5P034302 | protein_coding | NM_001024656 | ASPDH | -2.1250344 | 0.00001868 |
| ASHGA5P053998 | protein_coding | NM_001164417 | SSX2B | -2.1255614 | 8.974E-10 |
| ASHGA5P012860 | protein_coding | NM_152622 | MIER3 | -2.1261441 | 0.000008707 |
| ASHGA5P001684 | protein_coding | NM_000905 | NPY | -2.1264667 | 0.0073034 |
| ASHGA5P007987 | protein_coding | ENST00000338875 | HELT | -2.1302208 | 0.0000469 |
| ASHGA5P051682 | protein_coding | NM_004189 | SOX14 | -2.13177 | 7.267E-07 |
| ASHGA5P002451 | protein_coding | NM_139348 | BIN1 | -2.1342016 | 6.488E-07 |
| ASHGA5P049661 | protein_coding | ENST00000436615 | RAPGEFL1 | -2.1354976 | 0.00001072 |
| ASHGA5P038562 | protein_coding | NM_002662 | PLD1 | -2.1398424 | 0.000005994 |
| ASHGA5P007345 | protein_coding | NM_001711 | BGN | -2.1403732 | 4.364E-08 |
| ASHGA5P012115 | protein_coding | NM_138273 | C6orf25 | -2.1413293 | 1.689E-12 |
| ASHGA5P013074 | protein_coding | NM_003716 | CADPS | -2.1417416 | 0.000000176 |
| ASHGA5P036395 | protein_coding | NM_002374 | MAP2 | -2.1427039 | 0.000001237 |
| ASHGA5P002965 | protein_coding | NM_001012410 | SGOL1 | -2.1454681 | 0.000002247 |
| ASHGA5P013134 | protein_coding | NM_175611 | GRIK1 | -2.1477184 | 0.0036732 |
| ASHGA5P055162 | protein_coding | NM_024429 | PRKAG2 | -2.1498051 | 2.337E-07 |
| ASHGA5P033806 | protein_coding | NM_001134453 | DSG4 | -2.1514415 | 0.0003289 |
| ASHGA5P011732 | protein_coding | ENST00000373176 | AK1 | -2.1524469 | 2.585E-10 |
| ASHGA5P053129 | protein_coding | NM_001003795 | GTF2IRD2B | -2.1526248 | 9.999E-07 |
| ASHGA5P012622 | protein_coding | NM_014332 | SMPX | -2.1530784 | 8.173E-07 |
| ASHGA5P004303 | protein_coding | NM_001145211 | SLCO2B1 | -2.1542553 | 0.00001208 |
| ASHGA5P014058 | protein_coding | NM_001130160 | STOX1 | -2.1553595 | 0.0069661 |
| ASHGA5P003250 | protein_coding | NM_018312 | PPP6R3 | -2.1559913 | 2.245E-08 |
| ASHGA5P050703 | protein_coding | NM_004405 | DLX2 | -2.1565737 | 0.0001156 |
| ASHGA5P013452 | protein_coding | NM_001040102 | OPALIN | -2.1571391 | 0.00001977 |
| ASHGA5P000725 | protein_coding | NM_016188 | ACTL6B | -2.1611277 | 0.0085341 |
| ASHGA5P042039 | protein_coding | NM_001004473 | OR10K1 | -2.1620811 | 1.853E-09 |
| ASHGA5P026461 | protein_coding | NM_001145077 | LRRC10B | -2.1623237 | 0.000001183 |
| ASHGA5P011865 | protein_coding | NM_001171162 | ZMYM3 | -2.1640676 | 0.000008547 |
| ASHGA5P009723 | protein_coding | NM_018014 | BCL11A | -2.1664634 | 5.398E-09 |
| ASHGA5P055311 | protein_coding | NM_175054 | HIST4H4 | -2.1741835 | 0.000008972 |
| ASHGA5P004536 | protein_coding | NM_052960 | RBP7 | -2.1776389 | 0.0001399 |
| ASHGA5P052121 | protein_coding | ENST00000338566 | NPY5R | -2.178187 | 0.0044268 |
| ASHGA5P044916 | protein_coding | NM_022351 | NECAB1 | -2.1826541 | 5.812E-07 |
| ASHGA5P054473 | protein_coding | NM_001080449 | DNA2 | -2.1837257 | 6.523E-08 |
| ASHGA5P010694 | protein_coding | NM_003238 | TGFB2 | -2.1878512 | 9.882E-07 |
| ASHGA5P012356 | protein_coding | NM_181575 | AUP1 | -2.1905114 | 1.61E-08 |
| ASHGA5P050952 | protein_coding | NM_001083537 | FAM86B1 | -2.190635 | 2.616E-09 |
| ASHGA5P006704 | protein_coding | NM_172169 | CAMK2G | -2.1906805 | 0.0047044 |
| ASHGA5P012276 | protein_coding | NM_001008229 | MOG | -2.1929453 | 0.000002082 |
| ASHGA5P029408 | protein_coding | NM_007274 | ACOT7 | -2.1950781 | 9.685E-10 |
| ASHGA5P026260 | protein_coding | NM_031418 | ANO3 | -2.197956 | 0.0021898 |
| ASHGA5P012567 | protein_coding | NM_147169 | C9orf24 | -2.1993078 | 0.0001447 |
| ASHGA5P002738 | protein_coding | NM_172057 | KCNH2 | -2.1995663 | 4.286E-07 |
| ASHGA5P035312 | protein_coding | NM_032545 | CFC1 | -2.2062486 | 1.482E-07 |
| ASHGA5P051837 | protein_coding | ENST00000343267 | APOD | -2.2103934 | 0.0000518 |
| ASHGA5P002927 | protein_coding | NM_020802 | KIAA1377 | -2.2114993 | 0.0001098 |
| ASHGA5P034410 | protein_coding | ENST00000222033 | ZNRF4 | -2.2130835 | 0.0002423 |
| ASHGA5P039463 | protein_coding | ENST00000334306 | SOWAHB | -2.2134658 | 5.342E-09 |
| ASHGA5P043716 | protein_coding | ENST00000344575 | FZD9 | -2.2158088 | 0.00003876 |
| ASHGA5P049354 | protein_coding | NM_172347 | KCNG4 | -2.2165874 | 0.000006377 |
| ASHGA5P036194 | protein_coding | NM_004522 | KIF5C | -2.2178211 | 0.002388 |
| ASHGA5P012742 | protein_coding | NM_001166058 | RXFP2 | -2.2191475 | 0.00001963 |
| ASHGA5P008910 | protein_coding | NM_080921 | PTPRC | -2.2193471 | 0.00001355 |
| ASHGA5P013550 | protein_coding | NM_001040666 | STEAP2 | -2.220146 | 0.0181968 |
| ASHGA5P005390 | protein_coding | NM_003081 | SNAP25 | -2.2230276 | 0.0197628 |
| ASHGA5P006466 | protein_coding | NM_152341 | PAQR4 | -2.2251122 | 0.000003052 |
| ASHGA5P031422 | protein_coding | NM_022457 | RFWD2 | -2.2260563 | 2.868E-07 |
| ASHGA5P007556 | protein_coding | NM_001163788 | PTBP3 | -2.2266881 | 0.00001709 |
| ASHGA5P047716 | protein_coding | ENST00000369295 | ADRB1 | -2.2283158 | 0.0008489 |
| ASHGA5P017212 | protein_coding | NM_001103149 | PNMAL1 | -2.2302219 | 0.0000112 |
| ASHGA5P052011 | protein_coding | NM_001968 | EIF4E | -2.2304898 | 6.349E-07 |
| ASHGA5P003140 | protein_coding | NM_005139 | ANXA3 | -2.2318659 | 0.0066693 |
| ASHGA5P012274 | protein_coding | ENST00000376917 | MOG | -2.2347274 | 0.00001241 |
| ASHGA5P002306 | protein_coding | NM_001014986 | FOLH1 | -2.2365082 | 0.0005961 |
| ASHGA5P007328 | protein_coding | NM_153497 | TAB1 | -2.2373863 | 2.749E-10 |
| ASHGA5P027760 | protein_coding | NM_002234 | KCNA5 | -2.2402554 | 0.000241 |
| ASHGA5P000934 | protein_coding | NM_002854 | PVALB | -2.2480876 | 0.00002121 |
| ASHGA5P018897 | protein_coding | NM_003818 | CDS2 | -2.2525124 | 0.000005072 |
| ASHGA5P054493 | protein_coding | NM_172171 | CAMK2G | -2.2544761 | 7.329E-08 |
| ASHGA5P005312 | protein_coding | NM_004726 | REPS2 | -2.2576152 | 0.000004124 |
| ASHGA5P015460 | protein_coding | NM_001012512 | GRP | -2.2592361 | 0.0035449 |
| ASHGA5P007210 | protein_coding | NM_173573 | C11orf35 | -2.2596712 | 9.635E-07 |
| ASHGA5P054781 | protein_coding | NM_002233 | KCNA4 | -2.2677329 | 7.578E-08 |
| ASHGA5P005393 | protein_coding | NM_001130858 | PPIP5K1 | -2.2679586 | 0.000003489 |
| ASHGA5P009955 | protein_coding | NM_033396 | TNKS1BP1 | -2.2718252 | 3.137E-08 |
| ASHGA5P035736 | protein_coding | NM_001105569 | MSGN1 | -2.2783369 | 0.00000157 |
| ASHGA5P010039 | protein_coding | NM_032649 | CNDP1 | -2.2800648 | 0.0002685 |
| ASHGA5P054857 | protein_coding | NM_005838 | GLYAT | -2.2858734 | 0.000001743 |
| ASHGA5P042532 | protein_coding | NM_006813 | PNRC1 | -2.2893157 | 3.7E-12 |
| ASHGA5P007815 | protein_coding | NM_153499 | CAMKK2 | -2.290753 | 0.0003543 |
| ASHGA5P034713 | protein_coding | NM_001195256 | LOC100507003 | -2.2941549 | 0.0001528 |
| ASHGA5P037275 | protein_coding | ENST00000382822 | KRTAP19-8 | -2.2971728 | 0.00003136 |
| ASHGA5P041004 | protein_coding | ENST00000504595 | FBXL7 | -2.2975288 | 1.703E-07 |
| ASHGA5P000987 | protein_coding | ENST00000217043 | R3HDML | -2.2985609 | 0.00001915 |
| ASHGA5P005232 | protein_coding | NM_022788 | P2RY12 | -2.3031079 | 0.0010745 |
| ASHGA5P030034 | protein_coding | NM_015263 | DMXL2 | -2.3038985 | 0.0004699 |
| ASHGA5P029528 | protein_coding | NM_152447 | LRFN5 | -2.307649 | 0.0214531 |
| ASHGA5P004407 | protein_coding | ENST00000291670 | FTCD | -2.3080151 | 0.00001401 |
| ASHGA5P006623 | protein_coding | NM_017450 | BAIAP2 | -2.3085723 | 0.0009854 |
| ASHGA5P001284 | protein_coding | NM_000727 | CACNG1 | -2.3115117 | 6.835E-07 |
| ASHGA5P013172 | protein_coding | NM_031913 | ESYT3 | -2.3119528 | 1.75E-08 |
| ASHGA5P006876 | protein_coding | NM_153187 | SLC22A1 | -2.317347 | 0.00001041 |
| ASHGA5P012160 | protein_coding | ENST00000438075 | LST1 | -2.3190595 | 2.402E-09 |
| ASHGA5P016670 | protein_coding | NM_207491 | FAM190A | -2.3195482 | 9.774E-09 |
| ASHGA5P001970 | protein_coding | NM_144568 | TMEM55B | -2.3239382 | 2.836E-08 |
| ASHGA5P010349 | protein_coding | ENST00000372717 | DBNDD2 | -2.3264886 | 0.0119371 |
| ASHGA5P051635 | protein_coding | NM_014980 | STXBP5L | -2.3266636 | 0.0005603 |
| ASHGA5P007723 | protein_coding | NM_019859 | HTR7 | -2.3419392 | 0.000000597 |
| ASHGA5P049231 | protein_coding | NM_020988 | GNAO1 | -2.3422842 | 5.95E-11 |
| ASHGA5P044425 | protein_coding | NM_033512 | TSPYL5 | -2.3477043 | 6.288E-07 |
| ASHGA5P008564 | protein_coding | NM_001013031 | SORCS1 | -2.3485504 | 0.0002282 |
| ASHGA5P013602 | protein_coding | NM_002378 | MATK | -2.3507721 | 1.275E-07 |
| ASHGA5P052920 | protein_coding | NM_052868 | IGSF8 | -2.3519098 | 3.814E-11 |
| ASHGA5P043159 | protein_coding | NM_080744 | SRCRB4D | -2.3533021 | 2.473E-07 |
| ASHGA5P045644 | protein_coding | ENST00000355728 | OR2T6 | -2.3581495 | 0.00003456 |
| ASHGA5P019800 | protein_coding | NM_001178130 | EGF | -2.3588629 | 0.000002028 |
| ASHGA5P010855 | protein_coding | NM_001113381 | RGS4 | -2.360769 | 2.355E-08 |
| ASHGA5P004917 | protein_coding | NM_017726 | PPP1R14D | -2.3632908 | 0.00003506 |
| ASHGA5P052629 | protein_coding | ENST00000428701 | HLA-G | -2.364175 | 1.156E-07 |
| ASHGA5P008400 | protein_coding | NM_017525 | CDC42BPG | -2.3649015 | 0.000009075 |
| ASHGA5P014059 | protein_coding | NM_001130159 | STOX1 | -2.3657947 | 0.0045051 |
| ASHGA5P009743 | protein_coding | NM_207196 | ADAM15 | -2.3710094 | 6.468E-09 |
| ASHGA5P046579 | protein_coding | ENST00000373521 | PABPC1L2B | -2.3716267 | 8.834E-08 |
| ASHGA5P004268 | protein_coding | NM_033148 | DTNB | -2.3720841 | 0.00001783 |
| ASHGA5P043022 | protein_coding | NM_000168 | GLI3 | -2.3747359 | 0.00001449 |
| ASHGA5P013576 | protein_coding | ENST00000394836 | RAB3IL1 | -2.3750548 | 2.222E-09 |
| ASHGA5P006887 | protein_coding | NM_001039654 | ZNF550 | -2.3815203 | 6.197E-07 |
| ASHGA5P003427 | protein_coding | NM_002569 | FURIN | -2.3820358 | 3.156E-09 |
| ASHGA5P020719 | protein_coding | NM_001025253 | TPD52 | -2.3842208 | 4.574E-07 |
| ASHGA5P008586 | protein_coding | NM_001128324 | ZFAND4 | -2.3855368 | 0.00001022 |
| ASHGA5P005325 | protein_coding | NM_014914 | AGAP1 | -2.3869077 | 0.0003033 |
| ASHGA5P006540 | protein_coding | NM_182612 | PDDC1 | -2.3893657 | 0.00001144 |
| ASHGA5P006837 | protein_coding | NM_003027 | SH3GL3 | -2.3901554 | 0.0048195 |
| ASHGA5P018608 | protein_coding | ENST00000455515 | FXYD1 | -2.3944049 | 0.00005571 |
| ASHGA5P049751 | protein_coding | NM_198379 | CACNA1G | -2.3947671 | 0.000002537 |
| ASHGA5P046862 | protein_coding | NM_001042359 | PTPN20B | -2.3992062 | 0.0188618 |
| ASHGA5P013769 | protein_coding | NM_000103 | CYP19A1 | -2.4033058 | 0.000006576 |
| ASHGA5P029507 | protein_coding | ENST00000307169 | INSM2 | -2.4061209 | 0.0247344 |
| ASHGA5P007788 | protein_coding | NM_001146055 | SNCA | -2.4066492 | 0.0177285 |
| ASHGA5P045764 | protein_coding | ENST00000316157 | LARP4B | -2.4216687 | 0.000003941 |
| ASHGA5P012751 | protein_coding | NM_001035223 | RGL3 | -2.4248055 | 0.000009778 |
| ASHGA5P005582 | protein_coding | NM_023036 | DNAI2 | -2.4276354 | 0.00001263 |
| ASHGA5P015409 | protein_coding | NM_170686 | ZNF398 | -2.4334252 | 3.46E-11 |
| ASHGA5P052864 | protein_coding | ENST00000531224 | BCLAF1 | -2.4346103 | 2.151E-08 |
| ASHGA5P008783 | protein_coding | NM_172215 | CAMKK2 | -2.4416435 | 0.00006421 |
| ASHGA5P010956 | protein_coding | NM_001003396 | TPD52L1 | -2.4433176 | 0.0003618 |
| ASHGA5P004816 | protein_coding | ENST00000379133 | C9orf24 | -2.4499973 | 0.00008099 |
| ASHGA5P019835 | protein_coding | NM_001006932 | RPS6KA2 | -2.4522715 | 0.000005844 |
| ASHGA5P011441 | protein_coding | ENST00000371483 | O3FAR1 | -2.4567265 | 0.00004198 |
| ASHGA5P000706 | protein_coding | NM_032607 | CREB3L3 | -2.4581612 | 0.00003203 |
| ASHGA5P012491 | protein_coding | NM_178452 | DNAAF1 | -2.459594 | 0.0004079 |
| ASHGA5P007480 | protein_coding | NM_001220474 | PPFIA2 | -2.4676867 | 0.0027036 |
| ASHGA5P009278 | protein_coding | NM_022439 | MAL | -2.468316 | 6.727E-10 |
| ASHGA5P011579 | protein_coding | NM_003019 | SFTPD | -2.4688407 | 0.000003721 |
| ASHGA5P055678 | protein_coding | NM_024667 | VPS37B | -2.4722646 | 6.416E-13 |
| ASHGA5P034824 | protein_coding | NM_001002919 | FAM150B | -2.4751305 | 0.00001228 |
| ASHGA5P051095 | protein_coding | NM_000830 | GRIK1 | -2.4763034 | 0.0015525 |
| ASHGA5P009999 | protein_coding | NM_032781 | PTPN5 | -2.4825889 | 5.337E-08 |
| ASHGA5P010106 | protein_coding | NM_207195 | ADAM15 | -2.4874753 | 2.657E-09 |
| ASHGA5P012571 | protein_coding | ENST00000379179 | SERP2 | -2.4876721 | 0.00001798 |
| ASHGA5P026917 | protein_coding | NM_001012277 | PRAMEF7 | -2.4889778 | 1.074E-07 |
| ASHGA5P023464 | protein_coding | NM_004110 | FDXR | -2.4906359 | 2.485E-07 |
| ASHGA5P006487 | protein_coding | ENST00000319129 | FBF1 | -2.4916441 | 0.000003242 |
| ASHGA5P028842 | protein_coding | NM_001040429 | PCDH17 | -2.4954651 | 8.873E-09 |
| ASHGA5P018490 | protein_coding | NM_001122825 | ESRP1 | -2.4965534 | 2.049E-08 |
| ASHGA5P037940 | protein_coding | NM_152267 | RNF185 | -2.4980605 | 0.000001422 |
| ASHGA5P027566 | protein_coding | NM_018711 | SVOP | -2.4983673 | 0.00001387 |
| ASHGA5P049121 | protein_coding | ENST00000343070 | SCNN1B | -2.5016024 | 7.72E-13 |
| ASHGA5P036639 | protein_coding | NM_020341 | PAK7 | -2.5146608 | 0.0031908 |
| ASHGA5P055645 | protein_coding | ENST00000426426 | TMEM233 | -2.521332 | 3.372E-10 |
| ASHGA5P032398 | protein_coding | NM_030966 | KRTAP1-3 | -2.5214369 | 0.000003873 |
| ASHGA5P012342 | protein_coding | NM_152725 | SLC39A12 | -2.5232815 | 0.0021455 |
| ASHGA5P037665 | protein_coding | ENST00000248980 | RFPL2 | -2.5298434 | 8.344E-08 |
| ASHGA5P040268 | protein_coding | NM_001006655 | FAM149A | -2.5299634 | 0.0025883 |
| ASHGA5P041398 | protein_coding | NM_001964 | EGR1 | -2.5306499 | 0.0001439 |
| ASHGA5P004579 | protein_coding | NM_003385 | VSNL1 | -2.5325804 | 0.000005001 |
| ASHGA5P021355 | protein_coding | NM_001077441 | BCLAF1 | -2.535396 | 1.98E-08 |
| ASHGA5P009768 | protein_coding | NM_016950 | SPOCK3 | -2.5445914 | 0.0001343 |
| ASHGA5P047679 | protein_coding | NM_004210 | NEURL | -2.5480681 | 0.00001244 |
| ASHGA5P013922 | protein_coding | NM_001037671 | C12orf74 | -2.5498443 | 1.264E-08 |
| ASHGA5P015599 | protein_coding | NM_016532 | INPP5K | -2.555057 | 0.00001535 |
| ASHGA5P009833 | protein_coding | NM_001040159 | SPOCK3 | -2.5551575 | 0.00002566 |
| ASHGA5P003418 | protein_coding | NM_000781 | CYP11A1 | -2.565399 | 0.0001598 |
| ASHGA5P051457 | protein_coding | NM_004256 | SLC22A13 | -2.5791231 | 1.065E-07 |
| ASHGA5P043970 | protein_coding | NM_001001667 | OR6V1 | -2.5851929 | 1.862E-07 |
| ASHGA5P018204 | protein_coding | NM_001191013 | GSTO2 | -2.587878 | 0.000004025 |
| ASHGA5P000969 | protein_coding | NM_001130417 | SLC8A3 | -2.5951135 | 0.0068852 |
| ASHGA5P047280 | protein_coding | NM_201570 | CACNB2 | -2.611448 | 8.736E-11 |
| ASHGA5P014433 | protein_coding | NM_017951 | SMPD4 | -2.6203538 | 7.645E-07 |
| ASHGA5P001636 | protein_coding | NM_001135940 | MYOT | -2.6205094 | 0.0075934 |
| ASHGA5P015028 | protein_coding | NM_001082575 | RBFOX3 | -2.6236002 | 3.367E-07 |
| ASHGA5P034189 | protein_coding | NM_033256 | PPP1R14A | -2.6446567 | 5.327E-08 |
| ASHGA5P012615 | protein_coding | NM_001164756 | ASPH | -2.6590612 | 0.0001497 |
| ASHGA5P008435 | protein_coding | ENST00000343131 | SYCE1 | -2.6613089 | 2.666E-10 |
| ASHGA5P008239 | protein_coding | NM_001005336 | DNM1 | -2.6652733 | 0.000003734 |
| ASHGA5P003958 | protein_coding | NM_000320 | QDPR | -2.666475 | 0.0095191 |
| ASHGA5P010023 | protein_coding | NM_172081 | CAMK2B | -2.6695246 | 0.000002195 |
| ASHGA5P011709 | protein_coding | NM_022756 | MEAF6 | -2.6821312 | 0.001719 |
| ASHGA5P011860 | protein_coding | NM_001127371 | CDCA7L | -2.6822715 | 0.00002685 |
| ASHGA5P013090 | protein_coding | NM_153635 | CPNE9 | -2.6875066 | 6.282E-09 |
| ASHGA5P003549 | protein_coding | ENST00000270310 | FXYD7 | -2.6938808 | 0.0005775 |
| ASHGA5P047556 | protein_coding | NM_006829 | C10orf116 | -2.7025087 | 1.353E-07 |
| ASHGA5P049451 | protein_coding | ENST00000439858 | TMEM125 | -2.711054 | 1.116E-07 |
| ASHGA5P019737 | protein_coding | NM_198383 | CACNA1G | -2.7146807 | 9.143E-08 |
| ASHGA5P051886 | protein_coding | NM_144720 | JAKMIP1 | -2.7154007 | 0.00063 |
| ASHGA5P014414 | protein_coding | NM_014677 | RIMS2 | -2.7190861 | 0.00004131 |
| ASHGA5P001406 | protein_coding | NM_002821 | PTK7 | -2.7250393 | 0.000001283 |
| ASHGA5P037825 | protein_coding | NM_178134 | CYP4Z1 | -2.7257079 | 5.906E-09 |
| ASHGA5P018320 | protein_coding | NM_014741 | ATG13 | -2.7327286 | 9.538E-10 |
| ASHGA5P050556 | protein_coding | NM_003896 | ST3GAL5 | -2.7449338 | 0.000005603 |
| ASHGA5P026380 | protein_coding | NM_001206626 | TRIM49B | -2.7475968 | 2.495E-07 |
| ASHGA5P004859 | protein_coding | ENST00000298296 | MAGEC3 | -2.7568186 | 0.00009792 |
| ASHGA5P005398 | protein_coding | NM_016533 | NINJ2 | -2.756842 | 0.00002277 |
| ASHGA5P002411 | protein_coding | ENST00000457475 | CAMK2B | -2.7606339 | 0.0011821 |
| ASHGA5P004644 | protein_coding | NM_001443 | FABP1 | -2.762845 | 0.0015358 |
| ASHGA5P054066 | protein_coding | NM_001159560 | BEX5 | -2.7678898 | 0.000002656 |
| ASHGA5P032295 | protein_coding | NM_005165 | ALDOC | -2.7729834 | 0.0344202 |
| ASHGA5P006495 | protein_coding | NM_003182 | TAC1 | -2.7786573 | 0.0070587 |
| ASHGA5P018196 | protein_coding | NM_001165921 | SERPINF2 | -2.7792413 | 1.633E-07 |
| ASHGA5P007956 | protein_coding | NM_001191014 | GSTO2 | -2.7850444 | 0.000004339 |
| ASHGA5P051405 | protein_coding | NM_003178 | SYN2 | -2.7960151 | 1.256E-09 |
| ASHGA5P022401 | protein_coding | NM_001162997 | C17orf110 | -2.8107145 | 0.000009011 |
| ASHGA5P011301 | protein_coding | NM_001010917 | GOLGA7B | -2.8116061 | 1.763E-07 |
| ASHGA5P005671 | protein_coding | NM_001619 | ADRBK1 | -2.8135356 | 0.000002488 |
| ASHGA5P013467 | protein_coding | NM_001445 | FABP6 | -2.8143704 | 0.0003524 |
| ASHGA5P001467 | protein_coding | NM_172027 | ABTB1 | -2.8149751 | 2.633E-07 |
| ASHGA5P005286 | protein_coding | NM_002738 | PRKCB | -2.8182842 | 0.0061693 |
| ASHGA5P005791 | protein_coding | NM_002371 | MAL | -2.8184789 | 2.899E-11 |
| ASHGA5P050030 | protein_coding | NM_001025100 | MBP | -2.8247786 | 0.0014269 |
| ASHGA5P015209 | protein_coding | ENST00000538906 | ZNF668 | -2.8463867 | 0.00005399 |
| ASHGA5P009032 | protein_coding | NM_153748 | KCNC2 | -2.8496889 | 0.0290272 |
| ASHGA5P013472 | protein_coding | NM_152349 | KRT222 | -2.8553063 | 0.000003248 |
| ASHGA5P005066 | protein_coding | NM_000717 | CA4 | -2.8660811 | 0.00009103 |
| ASHGA5P052516 | protein_coding | ENST00000253490 | FAM153B | -2.8767843 | 4.544E-08 |
| ASHGA5P002493 | protein_coding | NM_001145057 | IL18BP | -2.8886038 | 2.951E-07 |
| ASHGA5P000872 | protein_coding | NM_005156 | PTBP3 | -2.8908343 | 0.00001093 |
| ASHGA5P001102 | protein_coding | NM_007029 | STMN2 | -2.892264 | 0.0022129 |
| ASHGA5P005389 | protein_coding | NM_001003395 | TPD52L1 | -2.8946377 | 0.0001049 |
| ASHGA5P053997 | protein_coding | NM_001097592 | XAGE1A | -2.9196058 | 4.01E-11 |
| ASHGA5P002220 | protein_coding | NM_001105243 | PCDH19 | -2.9208774 | 0.000000608 |
| ASHGA5P044718 | protein_coding | NM_002717 | PPP2R2A | -2.9263721 | 0.000000111 |
| ASHGA5P010262 | protein_coding | NM_153277 | SLC22A6 | -2.9305576 | 0.0010035 |
| ASHGA5P014248 | protein_coding | NM_001160132 | KCNQ5 | -2.9408937 | 0.000000422 |
| ASHGA5P048083 | protein_coding | NM_001193471 | FOLH1 | -2.9414021 | 0.000002642 |
| ASHGA5P008796 | protein_coding | NM_172082 | CAMK2B | -2.9539543 | 3.387E-07 |
| ASHGA5P005850 | protein_coding | NM_080862 | SPSB4 | -2.9611374 | 4.635E-11 |
| ASHGA5P044571 | protein_coding | ENST00000430474 | LY6H | -2.9694133 | 0.0007839 |
| ASHGA5P042608 | protein_coding | NM_003287 | TPD52L1 | -2.9704922 | 0.00004907 |
| ASHGA5P003290 | protein_coding | NM_015949 | GET4 | -3.0094156 | 3.171E-07 |
| ASHGA5P008779 | protein_coding | NM_172084 | CAMK2B | -3.0139695 | 0.00002247 |
| ASHGA5P053987 | protein_coding | NM_001098407 | GAGE2D | -3.0147682 | 1.281E-12 |
| ASHGA5P004430 | protein_coding | ENST00000292169 | S100A1 | -3.0228678 | 8.659E-07 |
| ASHGA5P048660 | protein_coding | NM_173090 | CAPN3 | -3.0258529 | 0.0001269 |
| ASHGA5P050923 | protein_coding | NM_001033087 | MACROD2 | -3.0303656 | 0.00009998 |
| ASHGA5P007587 | protein_coding | NM_001013838 | RLTPR | -3.0318443 | 1.188E-07 |
| ASHGA5P053985 | protein_coding | NM_001098406 | GAGE12J | -3.0411149 | 1.904E-12 |
| ASHGA5P055508 | protein_coding | NM_007191 | WIF1 | -3.0561474 | 0.0000218 |
| ASHGA5P012278 | protein_coding | NM_206810 | MOG | -3.0604241 | 0.000003293 |
| ASHGA5P009711 | protein_coding | NM_001126133 | TNNT1 | -3.0777262 | 0.0003234 |
| ASHGA5P054316 | protein_coding | NM_000740 | CHRM3 | -3.1094915 | 0.00003241 |
| ASHGA5P008627 | protein_coding | ENST00000420646 | SNCA | -3.1146317 | 0.0001163 |
| ASHGA5P027615 | protein_coding | ENST00000425217 | KSR2 | -3.115018 | 1.946E-07 |
| ASHGA5P001767 | protein_coding | NM_020939 | CPNE5 | -3.1159791 | 0.00001341 |
| ASHGA5P033763 | protein_coding | ENST00000359446 | C18orf1 | -3.1209567 | 0.000004981 |
| ASHGA5P046919 | protein_coding | NM_145178 | ATOH7 | -3.1213272 | 2.884E-07 |
| ASHGA5P047925 | protein_coding | ENST00000536684 | MTRNR2L8 | -3.1519612 | 0.0005931 |
| ASHGA5P006451 | protein_coding | NM_003018 | SFTPC | -3.1557995 | 1.566E-09 |
| ASHGA5P003156 | protein_coding | NM_001063 | TF | -3.1606289 | 0.0265597 |
| ASHGA5P034718 | protein_coding | NM_020650 | RCN3 | -3.1654306 | 8.018E-07 |
| ASHGA5P013867 | protein_coding | NM_172168 | NOXO1 | -3.2181614 | 1.408E-10 |
| ASHGA5P009588 | protein_coding | NM_001025090 | MBP | -3.2189915 | 0.0002024 |
| ASHGA5P003937 | protein_coding | NM_020987 | ANK3 | -3.2242161 | 0.00006495 |
| ASHGA5P006856 | protein_coding | NM_153836 | CREG2 | -3.2285393 | 5.016E-09 |
| ASHGA5P007688 | protein_coding | NM_004796 | NRXN3 | -3.259049 | 0.00001126 |
| ASHGA5P049182 | protein_coding | NM_152901 | PYDC1 | -3.2593478 | 7.001E-12 |
| ASHGA5P004906 | protein_coding | NM_139136 | KCNC2 | -3.2615093 | 7.355E-09 |
| ASHGA5P051186 | protein_coding | NM_001193414 | TUBA8 | -3.2699347 | 2.108E-10 |
| ASHGA5P010634 | protein_coding | NM_002924 | RGS7 | -3.2703051 | 0.00008752 |
| ASHGA5P016127 | protein_coding | NM_001130863 | ENPP2 | -3.2882292 | 0.000007449 |
| ASHGA5P005574 | protein_coding | NM_021094 | SLCO1A2 | -3.2957227 | 0.000001684 |
| ASHGA5P008770 | protein_coding | NM_013997 | TAC1 | -3.3102497 | 0.003989 |
| ASHGA5P044887 | protein_coding | NM_004133 | HNF4G | -3.3108112 | 1.061E-10 |
| ASHGA5P020134 | protein_coding | NM_032957 | RTEL1 | -3.3171186 | 0.000005292 |
| ASHGA5P048370 | protein_coding | NM_206927 | SYTL2 | -3.3258324 | 3.351E-07 |
| ASHGA5P003707 | protein_coding | NM_001882 | CRHBP | -3.3280072 | 0.0001097 |
| ASHGA5P003811 | protein_coding | NM_006334 | OLFM1 | -3.3328219 | 3.651E-09 |
| ASHGA5P028621 | protein_coding | NM_052910 | SLITRK1 | -3.3644869 | 0.0008822 |
| ASHGA5P009246 | protein_coding | NM_172083 | CAMK2B | -3.4130643 | 0.0000232 |
| ASHGA5P013869 | protein_coding | NM_020711 | ERMN | -3.428451 | 0.0001271 |
| ASHGA5P010760 | protein_coding | NM_001001431 | TNNT2 | -3.4534819 | 0.00000124 |
| ASHGA5P010148 | protein_coding | NM_002385 | MBP | -3.457508 | 0.000166 |
| ASHGA5P046591 | protein_coding | NM_020932 | MAGEE1 | -3.4620691 | 0.00001162 |
| ASHGA5P050696 | protein_coding | ENST00000412271 | DHRS9 | -3.4723734 | 0.00001612 |
| ASHGA5P052625 | protein_coding | NM_206812 | MOG | -3.4917197 | 0.000006265 |
| ASHGA5P045683 | protein_coding | NM_002771 | PRSS3 | -3.5208957 | 2.208E-11 |
| ASHGA5P007073 | protein_coding | NM_001928 | CFD | -3.5469251 | 3.977E-10 |
| ASHGA5P009001 | protein_coding | NM_022440 | MAL | -3.5507314 | 2.183E-08 |
| ASHGA5P055743 | protein_coding | ENST00000241463 | RASL11A | -3.5611751 | 3.582E-11 |
| ASHGA5P032479 | protein_coding | NM_203400 | RPRML | -3.5811951 | 0.00000939 |
| ASHGA5P009043 | protein_coding | NM_013996 | TAC1 | -3.5816577 | 0.0027262 |
| ASHGA5P034199 | protein_coding | ENST00000311308 | TTC9B | -3.5846283 | 0.0003129 |
| ASHGA5P011468 | protein_coding | NM_000954 | PTGDS | -3.588464 | 1.663E-07 |
| ASHGA5P033923 | protein_coding | NM_001085474 | LYPD8 | -3.6048111 | 6.641E-10 |
| ASHGA5P006745 | protein_coding | NM_001043353 | TPM3 | -3.6340198 | 0.000001102 |
| ASHGA5P055073 | protein_coding | NM_001206627 | TRIM49DP | -3.6525619 | 1.367E-09 |
| ASHGA5P013904 | protein_coding | NM_001040712 | PTPRD | -3.6678938 | 7.141E-10 |
| ASHGA5P010971 | protein_coding | NM_020399 | GOPC | -3.6763633 | 4.141E-09 |
| ASHGA5P002647 | protein_coding | NM_015347 | RIMBP2 | -3.7306039 | 2.006E-08 |
| ASHGA5P048822 | protein_coding | NM_153815 | RASGRF1 | -3.7426802 | 0.00000129 |
| ASHGA5P013791 | protein_coding | NM_001076677 | CLTA | -3.7957846 | 2.524E-07 |
| ASHGA5P054309 | protein_coding | NM_001105541 | NEFM | -3.804354 | 3.096E-08 |
| ASHGA5P002092 | protein_coding | NM_015722 | CALY | -3.8442985 | 6.052E-09 |
| ASHGA5P026474 | protein_coding | NM_012202 | GNG3 | -3.844859 | 0.000007704 |
| ASHGA5P013777 | protein_coding | NM_001025069 | ARPP21 | -3.850215 | 2.464E-08 |
| ASHGA5P008795 | protein_coding | NM_172078 | CAMK2B | -3.851243 | 0.000001194 |
| ASHGA5P006732 | protein_coding | NM_005397 | PODXL | -3.8929489 | 8.011E-08 |
| ASHGA5P004062 | protein_coding | NM_018008 | FEZF2 | -3.8966992 | 6.462E-07 |
| ASHGA5P004558 | protein_coding | NM_024709 | C1orf115 | -3.9157507 | 6.434E-11 |
| ASHGA5P033917 | protein_coding | NM_001004734 | OR14I1 | -3.9207988 | 9.625E-13 |
| ASHGA5P053340 | protein_coding | NM_002769 | PRSS1 | -3.9409721 | 3.026E-09 |
| ASHGA5P005832 | protein_coding | NM_007097 | CLTB | -3.9570814 | 0.00001485 |
| ASHGA5P046237 | protein_coding | NM_001012977 | PABPC1L2A | -4.006466 | 4.876E-09 |
| ASHGA5P050683 | protein_coding | NM_001009959 | ERMN | -4.0641371 | 0.00001919 |
| ASHGA5P002200 | protein_coding | NM_130811 | SNAP25 | -4.0998029 | 0.00002489 |
| ASHGA5P037952 | protein_coding | ENST00000397468 | RFPL3 | -4.1126368 | 2.678E-09 |
| ASHGA5P002037 | protein_coding | NM_015264 | KIAA0930 | -4.133161 | 7.802E-10 |
| ASHGA5P039824 | protein_coding | NM_000798 | DRD5 | -4.1556584 | 3.449E-09 |
| ASHGA5P055043 | protein_coding | NM_000260 | MYO7A | -4.5259812 | 3.874E-10 |
| ASHGA5P001690 | protein_coding | NM_007096 | CLTA | -4.5384055 | 1.894E-07 |
| ASHGA5P006651 | protein_coding | NM_212535 | PRKCB | -4.6206508 | 0.000001798 |
| ASHGA5P001394 | protein_coding | NM_006366 | CAP2 | -4.6405426 | 1.621E-07 |
| ASHGA5P055767 | protein_coding | NM_198404 | KCTD4 | -4.6771741 | 1.679E-08 |
| ASHGA5P014061 | protein_coding | NM_144704 | AIFM3 | -4.7197204 | 9.558E-09 |
| ASHGA5P013079 | protein_coding | NM_182935 | MOBP | -4.9955695 | 1.333E-08 |
| ASHGA5P001109 | protein_coding | NM_006158 | NEFL | -5.0171386 | 0.000003664 |
| ASHGA5P004214 | protein_coding | NM_001048 | SST | -5.0903609 | 7.674E-09 |
| ASHGA5P006858 | protein_coding | NM_172216 | CAMKK2 | -5.0991471 | 1.685E-12 |
| ASHGA5P016992 | protein_coding | NM_145892 | RBFOX1 | -5.6308088 | 1.243E-08 |
| ASHGA5P041370 | protein_coding | NM_175873 | SOWAHA | -5.7110132 | 1.837E-10 |
| ASHGA5P000975 | protein_coding | NM_006032 | CPNE6 | -5.7459669 | 9.129E-10 |
| ASHGA5P007490 | protein_coding | NM_001018060 | AIFM3 | -6.2418073 | 3.402E-09 |
| ASHGA5P012275 | protein_coding | NM_002433 | MOG | -6.3308212 | 0.00009986 |
| ASHGA5P053443 | protein_coding | NM_000756 | CRH | -6.3324623 | 0.00001165 |
| ASHGA5P052039 | protein_coding | NM_001148 | ANK2 | -6.3892014 | 5.459E-09 |
| ASHGA5P009198 | protein_coding | NM_022438 | MAL | -6.3960839 | 2.491E-07 |
| ASHGA5P035038 | protein_coding | NM_007008 | RTN4 | -6.4149795 | 0.00009134 |
| ASHGA5P005189 | protein_coding | NM_001043351 | TPM3 | -6.4783756 | 0.000002573 |
| ASHGA5P036812 | protein_coding | NM_014477 | TP53TG5 | -6.5595094 | 4.078E-07 |
| ASHGA5P051542 | protein_coding | NM_080865 | GPR62 | -6.6488054 | 4.502E-11 |
| ASHGA5P006859 | protein_coding | NM_006549 | CAMKK2 | -8.1151986 | 7.183E-11 |
| ASHGA5P049105 | protein_coding | NM_001014444 | CRYM | -8.1189078 | 0.00002163 |
| ASHGA5P027093 | protein_coding | NM_006248 | PRB2 | -8.3891947 | 1.218E-08 |
| ASHGA5P051995 | protein_coding | NM_006168 | NKX6-1 | -8.5387973 | 0.000000348 |
| ASHGA5P026716 | protein_coding | NM_001105522 | TRIM49L1 | -8.9437678 | 3.173E-11 |
| ASHGA5P009528 | protein_coding | NM_145893 | RBFOX1 | -10.471883 | 2.811E-10 |
| ASHGA5P055729 | protein_coding | NM_006783 | GJB6 | -12.078979 | 3.63E-08 |
