# Supplementary figures and images for: LINC01287 facilitates proliferation, migration, invasion and EMT of colon cancer cells via miR-4500/MAP3K13 pathway
Source: BMC Cancer. 2021 Jul 6;21:782. doi: 10.1186/s12885-021-08528-7 (PMC8259379; doi:10.1186/s12885-021-08528-7)

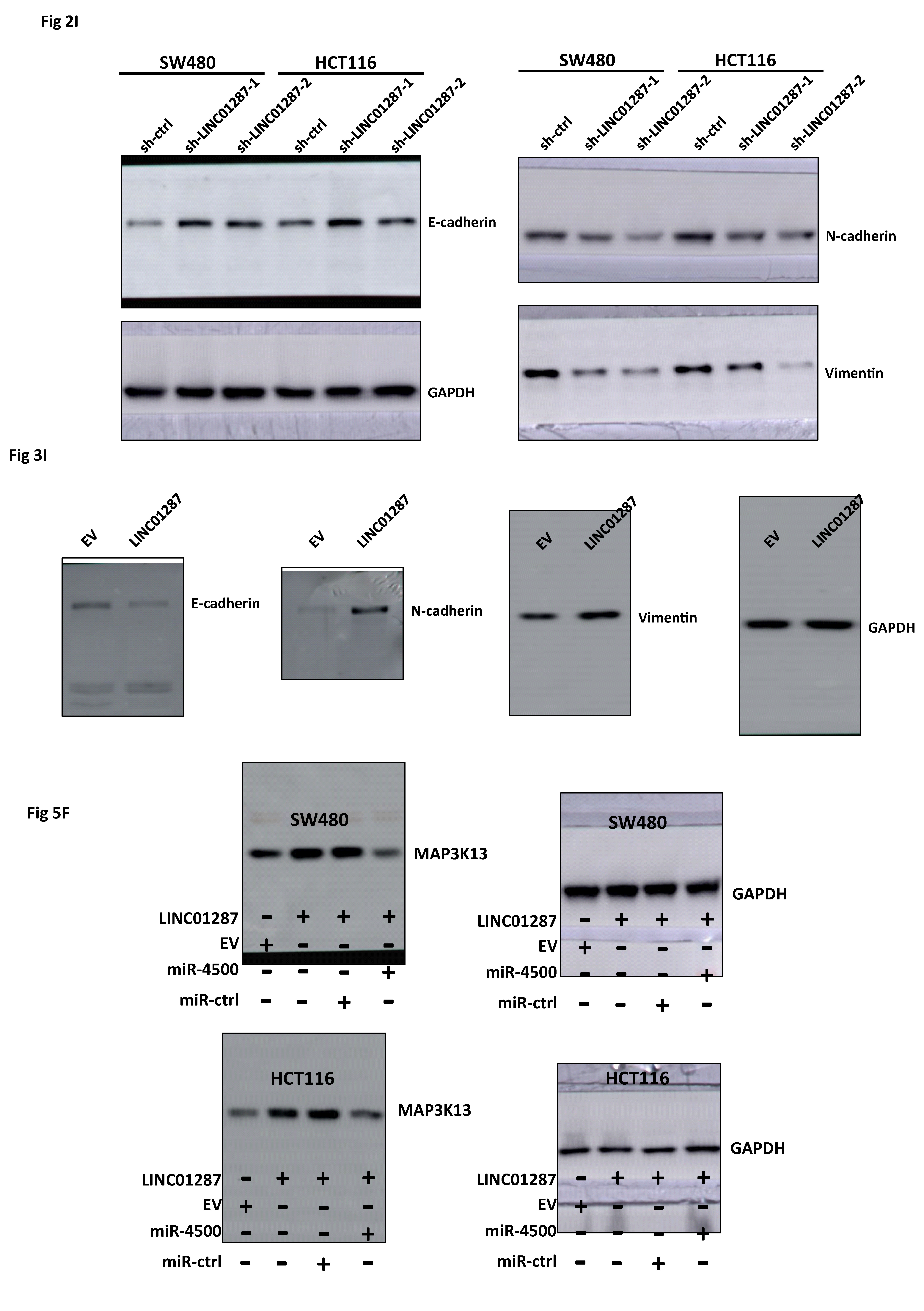

Supplement: Supplementary file 3 — Additional file 3. [file 12885_2021_8528_MOESM3_ESM.jpg]

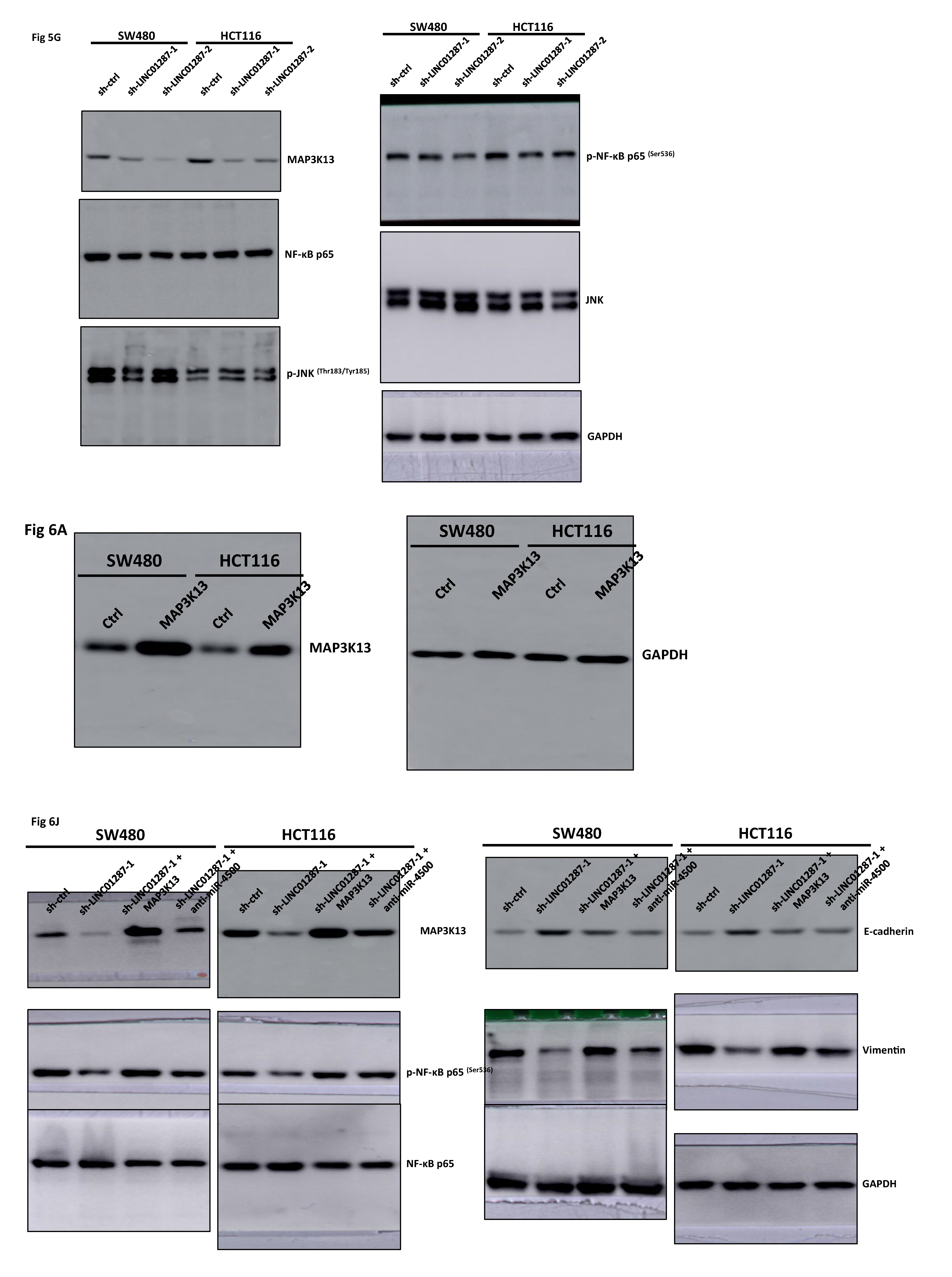

Supplement: Supplementary file 4 — Additional file 4. [file 12885_2021_8528_MOESM4_ESM.jpg]
